# A phase 1/2 study of DS-1594 menin inhibitor in relapsed/refractory acute leukemias

**DOI:** 10.1186/s13045-025-01757-4

**Published:** 2025-11-27

**Authors:** Jayastu Senapati, Marina Konopleva, Ghayas C. Issa, Elias Jabbour, Tapan Kadia, Courtney DiNardo, Gautam Borthakur, Naveen Pemmaraju, Nicholas J. Short, Musa Yilmaz, Indraneel Deshmukh, Joie Alvarez, Sanam Loghavi, Guilin Tang, Hussein A. Abbas, Michael Andreeff, Kapil Bhalla, Narasimha M. Midde, Nabil Said, Amy Noyalis, Derek E. Mires, Jing Ning, Lianchun Xiao, Farhad Ravandi, Guillermo Garcia-Manero, Hagop M. Kantarjian, Naval G. Daver

**Affiliations:** 1 Department of Leukemia, The University of Texas MD Anderson Cancer Center, 77030 Houston, TX USA; 2 Department of Hematopathology, The University of Texas MD Anderson Cancer Center, Houston, USA; 3 Daiichi Sankyo, Inc, Basking Ridge, NJ USA; 4 Department of Biostatistics, The University of Texas MD Anderson Cancer Center, Houston, USA; 5 Present Address: Department of Medical Oncology, Montefiore Einstein Cancer Center, New York, USA

**Keywords:** Acute myeloid leukemia, Menin inhibitor, KMT2A, NPM1, Differentiation syndrome

## Abstract

Several menin inhibitors are in development targeting menin dependent leukemias, however available preclinical results show variable level of activity. We report the phase 1 portion (to establish a recommended phase 2 dose [RP2D]) and pharmacokinetic analysis of a phase 1/2 first-in-human clinical trial of DS-1594b menin inhibitor. Eligible patients included adults (≥ 18 years of age) with relapsed/refractory (R/R) acute myeloid leukemia (AML) or acute lymphoblastic leukemia (ALL) including but not restricted to those with *KMT2A*-rearrangement (r) or *NPM1* mutation. Seventeen patients at a median of age 56 years (range, 19–82 years) were treated, 15 (88%) had R/R AML, and 2 (12%) had R/R B-ALL; 9 (53%) had a *KMT2A*-r but none had an *NPM1* mutation. The median prior lines of therapy was 3 (range 1–8) and 5 patients (29%) had received prior menin inhibitors. Five dose escalation cohorts were evaluated; no RP2D was established, and the trial was stopped at phase 1 due to a decision by supporting company due to lack of efficacy at studied dose levels and portfolio realignment. Differentiation syndrome (DS) was seen in 5 patients (29%); 2 in cohort 1 (70 mg twice daily, *n* = 4) 1 each had grade 1 and grade 4 DS, 3 patients in cohort 2 (50 mg twice daily/100 mg daily, *n* = 4) of whom 2 had grade 2 and 1 patient had grade 3 DS (considered as dose limiting toxicity). No DS was noted at cohort 3 (20 mg/day), and in subsequent dose-escalation cohorts (cohorts 4 and 5) a lead-in ramp-up dosing starting at 20 mg/day was instituted to improve tolerability. Other relevant treatment emergent adverse events of grade ≥ 3 included infections; pneumonia and febrile neutropenia in 7 patients each (41%), and sepsis in 6 patients (35%). No study drug related deaths were noted. No patient achieved a response, however 4 patients (23%) had > 25% bone marrow blast reduction. Pharmacokinetic analysis showed DS-1594b reached maximum concentration approximately in 2 h with total exposure increasing with escalating doses and reached stead-state by Cycle 1 Day 8. DS-1594b showed limited efficacy at the doses tested but appeared safe with a lead-in dosing approach.

**Supplementary Information:**

The online version contains supplementary material available at 10.1186/s13045-025-01757-4.

## Introduction

Genomically inspired therapies in acute myeloid leukemia (AML) have led to improvement in outcomes in subsets of AML with targetable genomic aberrations [1–3]. AML or acute lymphoblastic leukemia (ALL) with *KMT2A* gene rearrangement (r), AML with *NPM1* mutation (*NPM1*^mut^) and few other subtypes are dependent on downstream MEIS/HOXA activation for leukemogenesis and are amenable to menin inhibition [4]. Menin inhibitors prevent the interaction of menin with partner protein products of *KMT2A* and *NPM1* preventing activation of the downstream leukemogenic *MEIS/HOXA* genes [5, 6]. Recently revumenib, a first in class oral menin inhibitor was approved for patients >1 year of age with relapsed or refractory (R/R) *KMT2A*-r acute leukemias (AML, ALL or mixed phenotypic acute leukemia [MPAL]). In the registrational Phase 1b/2 AUGMENT 101 trial, revumenib led to an overall response rate (ORR) of 63% with a complete response + CR with incomplete hematological recovery (CR + CRh) rate of 23% in patients with R/R *KMT2A-*r ALL/AML/MPAL or R/R *NPM1*^mut^ AML [7].

Despite the promising response rates in the salvage setting with revumenib in these R/R leukemias, the median duration of response (DOR) for patients with an ORR was 4.3 months and CR + CRh 6.4 months [8]. The drug was reasonably well tolerated with notable grade 3 or higher toxicities including differentiation syndrome (DS) in 16% and QTc prolongation in 14% patients. Menin mutations while on treatment with revumenib were noted, likely playing a role in resistance to revumenib by altering the binding site of the menin inhibitor [9]. Therefore, It is important to develop newer agents that may prevent emergence or bypass resistance mediated by these on target MEN mutations. Recently a novel menin inhibitor, bleximenib, showed efficacy in cell lines of *KMT2A-*r leukemia harboring the menin mutations (*MEN1*-M327I or *MEN1*-T349M) [10]. Similarly other novel menin inhibitos ziftomenib and enzomenib are being evaluated in clinical trials for R/R AML and ALL with target aberrations [11, 12].

While *NPM1* and *KMT2A*-r leukemias are the commonly encountered genetic aberrations that are dependent on menin pathway, there are several other relatively smaller subgroups, particularly in AML (like AML with *NUP98* rearrangement, *UBTF1* mutation, others), which are also MEIS/HOX dependent and may be amenable to menin inhibition [4, 6, 13]. Additionally, patients with these leukemias have inferior outcomes and the majority of them relapse with short remission durations [14–16]. Thus, there remains a major unmet need to develop novel agents for these leukemias as well.

DS-1594b is an oral menin inhibitor which showed preclinical activity through promoting differentiation and reducing serial colony forming capabilities in cell lines and patient derived xenograft models of *KMT2A*-r and *NPM1*^mut^ AML [17]. In further pre-clinical studies, DS-1594b showed synergism with venetoclax in cell lines and primary patient derived samples of *KMT2A*-r and *NPM1*^mut^ AML [18]; differentiation of leukemic cells appeared to be the primary mechanism of action of the drug which showed activity even in venetoclax resistant cells. Given this preclinical evidence, we initiated an open-label phase 1b/2 study with DS-1594b in patients with R/R acute leukemias.

## Methods

This phase 1b/2 single-arm, open label, single center first-in-human study was designed to treat adult patients (≥18 years of age) with R/R acute leukemias. All patients signed an informed consent; the study was approved by the Institutional Reviewed Board at MDACC (ClinicalTrials.gov NCT04752163 and conducted as per the Declaration of Helsinki. The study was initiated in March 2021.

### Phase 1

This phase included patients with R/R AML or R/R ALL regardless of *KMT2A* or *NPM1* status and implemented a Bayesian optimal interval (BOIN) design to define the maximum tolerable dose (MTD) and establish the recommended phase 2 dose (RP2D). Details are in the supplement protocol. Although all R/R AML/ALL was allowed during the Phase 1 dose-escalation, the goal was to treat at least 3 subjects with *KMT2A*-r or *NPM1*^mut^ at each dose level that was anticipated to be biologically active. This would allow for optimal evaluation of safety and efficacy of that dose in the presumed target population to guide selection of the optimal RP2D.

The starting dose of DS1594b was 70 mg twice daily (BID) approximately every 12 h (Phase 1, Cohort 1) administered orally based on an estimated clinical half-life of 7–8 h. The study phases and dose escalation of phase 1 is shown in Supplemental Fig. 1 (Figure S1**).** The rationale for the initial dose selection was based on extrapolation of data from cell line efficacy studies and toxicology studies in rodents and primates, and also to mitigate risks of QTc prolongation as detailed in the Supplement study protocol. Dose limiting toxicity (DLT) is defined in Sect. 3.2.2 of study protocol in the supplement. The PK showed higher and earlier drug accumulation than preclinically predicted resulting in the opening of a parallel once daily (QD) cohort starting at cohort 2. DS was considered as an AE of special interest and special DS mitigating interventions were included in the study protocol. After a review by the scientific review committee in September 2021, the starting dose was reduced to 20 mg once daily (QD) (Phase 1, cohort 3) given 3 cases of grade 2/3 DS and ensuing dose interruption in patients treated on the 50 mg BID and 100 mg QD doses. A lead-in dosing with weekly ramp-up was employed to sensitize the subjects and to evaluate the role of step-wise lead-in dosing in reducing the risk of DS (Phase 1, cohort 4 onwards). The starting dose for the lead-in period was 20 mg (Figure S2, Table S1).

#### Intra-patient dose escalation

Intra-patient dose escalation of DS-1594b was permitted for patients enrolled in the phase 1 of the study, to a dose that had been deemed safe and tolerable in at least 3 DLT evaluable patients, patients who were considered to benefit from ongoing DS-1594b, and had not had a DLT at a lower dose, after approval of the scientific review committee. 

### Phase 2

The phase 2 portion of the study was planned to be a multicohort phase (4 cohorts) including DS-1594b monotherapy and combination therapy arms in patients with *KMT2A-*r AML/ALL or *NPM1*^mut^ AML. The study did not proceed to phase 2 and is not reported in the manuscript.

### Patients and eligibility

Adult patients (≥18 years of age) with R/R AML/ALL were eligible; eligibility for phase 1 was agnostic of *KMT2A* and *NPM1* status and for phase 2 patients had to have *KMT2A-*r or *NPM1*^mut^ as mentioned previously. Eligible patients had to have ECOG performance status 0–2 with adequate liver and kidney function (Sect. "Discussion" of study protocol). Patients with previous myelodysplastic syndrome (MDS) or chronic myelomonocytic leukemia (CMML) were eligible at the time of transformation to AML if they had received prior hypomethylating agent for MDS/CMML. Patients with controlled central nervous system (CNS) leukemia were eligible. Women of childbearing age-group were eligible if they were on adequate contraceptive therapy. Patients with uncontrolled or significant cardiovascular disease were not eligible and detailed in the study protocol. Patients who had undergone an allogeneic hematopoietic stem cell transplantation (HSCT) within 90 days before screening were not eligible as were patients with clinically significant (> grade 1) graft versus host disease [GVHD] (minor grade skin GVHD was allowed). All patients received antifungal prophylaxis during period of neutropenia with echinocandins or isavuconazole.

### Phase 1 study objectives

The primary objective of phase 1 was to establish the MTD and RP2D of DS-1594b monotherapy and the overall safety profile. Efficacy was measured as rate of CR/CRh within 3 months of therapy initiation as per European LeukemiaNet 2017 criteria [19]. Safety analysis was done in the safety population which included all patients who had received at least 1 dose of DS-1594.

### Statistical methods

Phase 1 used a BOIN design to arrive at the MTD and RP2D (Figure S2) and detailed in the study protocol Sect. "Results". All patients who received at least one dose of DS-1594b were eligible for safety and efficacy analysis. The study was monitored for futility and toxicity using the Bayesian method of Thall, Simon and Estey [20].

Event free survival (EFS) and overall survival (OS) were estimated using the Kaplan-Meier method. EFS was calculated from the date of start treatment to the date of disease relapse or date of death, whichever occurs first, and censored at the date of last follow-up if alive and without relapse. OS was calculated from the date of treatment start to the date of death or last follow-up and censored if alive at last data cutoff.

### Pharmacokinetic analysis

The pharmacokinetic (PK) dataset consisted of all subjects who had at least one blood sample providing evaluable PK data for DS-1594b (*n* = 14). Subjects with missing/reduced/interrupted doses for at least three consecutive days prior to PK sampling days (except for Cycle 1 Day 1) were not included in the analysis. The PK parameter estimates were generated for plasma DS-1594 on Day 1, Day 8 and Day 15 from Cycle 1 using the plasma concentrations in the respective samples and the actual time calculated relative to the time of drug administration. Details of time of PK sample collection are in Sect. 8.4 of study protocol in the supplement.

The following parameters were calculated: area under the plasma concentration-time curve (AUC) extrapolated from time zero to 12 h (AUC_12h) and 24 h (AUC_24h), AUC from dosing interval (AUCtau), AUC from time zero to infinity (AUCinf), maximum observed plasma concentration (Cmax), time of maximum observed plasma concentration (Tmax), apparent terminal phase half-life (t1/2), apparent clearance (CL/F), apparent volume of distribution (Vz/F). AUCinf, half-life, CL/V, and Vz/F are only calculated on Day 1. Partial AUCs (AUC_12h and AUC_24h) and t1/2 were only reported if elimination rate constant (λz) values were > 3 and R2 adjusted was > 0.7. AUCinf values were only reported if AUC % extrapolated was < 20%. PK parameters AUC_12h and AUC_24h were used for assessment of the accumulation ratio (AR) for BID and QD dosing regimen, respectively.

Subjects from step-up dosing cohorts were analyzed the same as cohorts without lead-in dosing since the observed concentrations after lead-in doses are near below the limit of quantification (BLQ) prior to target dose administration (50 mg QD or 100 mg QD). Following this approach, data from only subject from step-up dosing with target dose of 100 mg QD cohort was grouped together with 100 mg QD cohort for the analysis. Pharmacokinetic (PK) data were analyzed using standard NCA method as implemented in validated PK data analysis software Phoenix WinNonlin version 8.3.4.

## Results

**Fig. 1:**
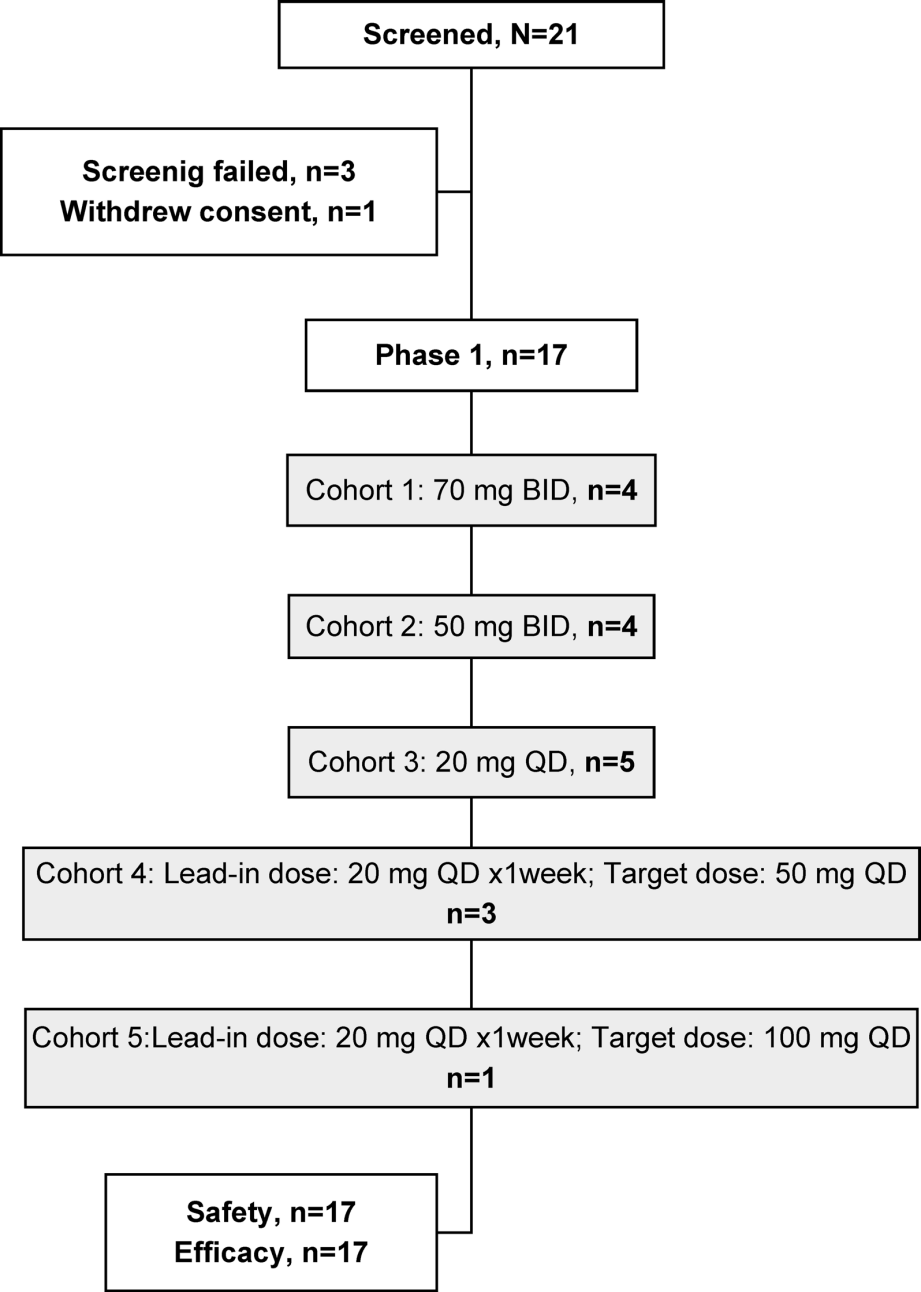
Study consort diagram. BID, twice daily; QD, once

From April 2021 to June 2022 a total of 21 patients were screened, and 17 patients were treated on the study, all in the phase 1 dose escalation portion. Three patients were screen failure, and one patient withdrew consent **(**Fig. 1**).** The median age of study patients was 56 years (range 19–82 years) and 8 (47%) were ≥ 60 years of age **(**Table 1**).** Fifteen patients (88%) had R/R AML, and 2 patients (12%) had R/R B-ALL. Nine patients (53%) had a *KMT2A*-r (8/15 [53%] patients with AML and 1/2 [50%] patient with B-ALL). No patient had an *NPM1* mutation. Six patients (40%) with AML had secondary AML, which included previously treated MDS in 5 patients and polycythemia vera in one patient. The median prior lines of therapy was 3 (range 1–8) and the regimen was the first salvage therapy in only 2 patients (one patient each with AML and B-ALL). All patients with AML had received prior venetoclax; one patient with B-ALL had received prior blinatumomab and one patient with B-ALL had received prior venetoclax. Five patients (29.4%), all with *KMT2A*-r AML, had received prior menin inhibitors as part of clinical trials (2 patients had received revumenib, one patient each had received ziftomenib and bleximenib and one patient had received both revumenib and ziftomenib). Overall, 6 patients (35.2%) (5 AML, 1 B-ALL) had received an HSCT in the past (3 patients had received two prior HSCTs).

**Table 1:** Baseline characteristics of study patients (*n* = 17)

Parameters	*N*(%), median [range]	
Age, years	Age ≥ 60 years	56 [19–82] 8 (47)
Gender	Female	5 (29)
Race	White Black Asian	13 (76) 3 (18) 1 (6)
Ethnicity	Hispanic	1 (6)
Diagnosis	AML • Treated MDS • Post MPN ALL	15 (88) 5 (29) 1 (6) 2 (12)
Previous therapy	Previous lines of therapy • One prior line only Venetoclax Menin inhibitor HSCT	3 [1–8] 2 (18) 16 (94) 5 (29) 6 (35)
Genomics	*KMT2A-*rearranged *NPM1* *TP53*	9 (53) 0 3 (18)
BM blasts (%)		60 [1–90]
WBC (x10^9^/cumm) Platelets (x10^9^/cumm)		2.7 [0.1–10.1] 24 [1–124]

AML, acute myeloid leukemia; MDS, myelodysplastic syndrome; MPN, myeloproliferative neoplasm; ALL, acute lymphoblastic leukemia; HSCT, allogeneic hematopoietic stem cell transplantation; BM, bone marrow

**Table 2:** A. All treatment emergent adverse events ≥ grade 3, regardless of attribution

Adverse event ≥ grade 3	Total	Grade 1–2	Grade 3–5	Grade 3	Grade 4	Grade 5
Infection related						
Pneumonia	8 (47)	1 (6)	7 (41)	3 (18)	3 (18)	1 (6)
Febrile Neutropenia	6 (35)	0	6 (35)	6 (35)	0	0
Sepsis	6 (35)	0	6 (35)	3 (18)	1 (6)	2 (12)
Skin/soft tissue infection	6 (35)	4 (23)	2 (12)	2 (12)	0	0
Bacteremia	6 (35)	6 (35)	0	0	0	0
Sinusitis	2 (12)	0	2 (12)	1 (6)	1 (6)	0
COVID-19	2 (12)	2 (12)	0	0	0	0
CMV reactivation	1 (6)	0	1 (6)	0	0	0
Rhinovirus	1 (6)	1 (6)	0	0	0	0
Cardiovascular and Respiratory						
Dyspnea	6 (35)	6 (35)	0	0	0	0
Respiratory failure/hypoxia	5 (36)	1 (6)	4 (23)	2 (12)	2 (12)	0
Sinus tachycardia	4 (23)	4 (23)	0	0	0	0
Sinus bradycardia	3 (18)	3 (18)	0	0	0	0
Rhinorrhea/Rhinitis	3 (18)	3 (18)	0	0	0	0
Syncope	2 (12)	0	2 (12)	2 (12)	0	0
Hypertension	2 (12)	1 (6)	1 (6)	0	1 (6)	0
Pericarditis	1 (6)	0	1 (6)	0	1 (6)	0
Atrial fibrillation	1 (6)	1 (6)	0	0	0	0
Gastrointestinal/hepatobiliary						
Nausea/vomiting	8 (47)	6 (35)	2 (12)	2 (12)	0	0
Transaminase elevation	7 (41)	6 (35)	1 (6)	1 (6)	0	0
Diarrhea	5 (36)	5 (36)	0	0	0	0
Hyperbilirubinemia	4 (23)	2 (12)	2 (12)	2 (12)	0	0
Abdominal pain	4 (23)	3 (18)	1 (6)	1 (6)	0	0
Mucositis	1 (6)	0	1 (6)	1 (6)	0	0
Nervous system						
Headache	3 (18)	2 (12)	1 (6)	1 (6)	0	0
Seizure	1 (6)	0	1 (6)	0	1 (6)	0
Bleeding/coagulopathy						
Epistaxis	4 (23)	2 (12)	2 (12)	2 (12)	0	0
Hematuria	4 (23)	3 (18)	1 (6)	1 (6)	0	0
Retroperitoneal hemorrhage	1 (6)	0	1 (6)	0	0	1 (6)
Intracranial hemorrhage	1 (6)	0	1 (6)	0	0	1 (6)
Hypofibrinogenemia	1 (6)	0	1 (6)	1 (6)	0	0
Hematological						
Anemia	5 (29)	0	5 (29)	4 (23)	1 (6)	0
Differentiation syndrome	5 (29)	3 (18)	2 (12)	1 (6)	1 (6)	0
Leukocytosis	2 (12)	0	2 (12)	0	2 (12)	0
Renal/Urogenital						
Acute kidney injury/elevated creatinine	7 (41)	5 (29)	2 (12)	2 (12)	0	0
Penile pain	1 (6)	0	1 (6)	1 (6)	0	0
Metabolic						
Hyperphosphatemia	8 (47)	7 (41)	1 (6)	1 (6)	0	0
Hypokalemia	6 (35)	3 (18)	3 (18)	1 (6)	2 (12)	0
Hyperuricemia	4 (23)	4 (23)	0	0	0	0
Hypomagnesemia	4 (23)	4 (23)	0	0	0	0
Hypocalcemia	4 (23)	4 (23)	0	0	0	0
Tumor lysis syndrome	2 (12)	1 (6)	1 (6)	0	1 (6)	0
Hyperglycemia	2 (12)	2 (12)	0	0	0	0
Hypercalcemia	2 (12)	2 (12)	0	0	0	0
Elevated lactate	2 (12)	2 (12)	0	0	0	0
Hypermagnesemia	1 (6)	0	1 (6)	1 (6)	0	0
Hypernatremia	1 (6)	1 (6)	0	0	0	0
Miscellaneous						
Malaise/fatigue	4 (23)	0	4 (23)	4 (23)	0	0
Bone pain	3 (18)	1 (6)	2 (12)	2 (12)	0	0
Blurred visions	2 (12)	2 (12)	0	0	0	0
Retinal hemorrhage	1 (6)	0	1 (6)	1 (6)	0	0
Leukemic optic nerve involvement	1 (6)	0	1 (6)	1 (6)	0	0

### Safety analysis

Four patients were initially treated on the starting dose of 70 mg BID (phase 1 cohort 1); one patient had grade 3 DS (considered a DLT). In the next dose level of 50 mg BID/100 mg daily (QD) (Cohort 2), 3 patients had a related DS (grade 2 DS in two patients and grade 3 DS in one patient) and detailed later. None of these patients had received the lead-in dosing as this was not part of the protocol at that time. As per scientific review committee recommendations after the high DS incidence, the dose was further reduced to 20 mg QD and 4 patients were treated at this dose level (Cohort 3). No DLTs were observed at this dose; subsequently 3 patients were treated at 50 mg/day with a 20 mg lead-in dosing (Cohort 4) and one patient at 100 mg/day with 20 mg followed by 50 mg lead-in dosing (Cohort 5). No further DLTs were observed with this approach. Patients received a median of 2 cycles of therapy (range 1–5) and 11 patients (64.7%) received ≥2 cycles of therapy. No patient was treated on the phase 1 sub-studies for dosing interaction with food or strong CYP3A4 inhibitors.

The most common treatment emergent grade 3 non-hematological AEs irrespective of attribution were infections: pneumonia/lung infection in 8 patients (47%), which included grade 3 in three patients, grade 4 in three patients and grade 5 in one patient, febrile neutropenia in 6 patients (35.3%) (all grade 3) and sepsis in 6 patients (35.3%) (grade 3 in three patients, grade 4 in one patient and grade 5 in two patients). Among the patients with pneumonia, 1 patient had a proven fungal (aspergillus) pneumonia, and 2 patients had probable fungal pneumonia based on clinical and radiological findings. Other infectious AEs included soft tissue infection/myositis and sinusitis in 2 patients each and cytomegalovirus reactivation in 1 patient (all grade 3). All grade relevant TEAEs irrespective of attribution are listed in Table 3. AEs of special interest included QTc prolongation and combined elevation of bilirubin and aminotransferase **(**Table 3**);** no patient had QTc prolongation, however one patient had possibly drug related grade 4 pericarditis and was taken off the study treatment. One patient had grade 2 atrial fibrillation, considered unrelated to the drug. Three patients (17.6%) had combined elevations of bilirubin and alanine aminotransferase (two of these bilirubin elevations were grade 3). Grade 3 treatment emergent anemia was seen in 5 patients (29.4%). The median packed red blood cell transfusion requirement during the first cycle was 3 units (range 0–13) and median platelet requirement was 7 units (range 0–36 units).

**Table 3:** Selected adverse events, regardless of attribution

Adverse events of special interest	All	Grade 1	Grade 2	Grade 3	Grade 4	Grade 5
Differentiation syndrome	5 (29)	1 (6)	2 (12)	1 (6)	1 (6)	0
Combined bilirubin and transaminase elevation	3 (18)	0	1 (6)	2 (12)	0	0
Atrial fibrillation	1 (6)	0	1 (6)	0	0	0
QTc prolongation	0	0	0	0	0	0

Overall, five patients (29.4%) had AEs suggestive of a DS in this study (Table S2). In the 70 mg BID starting dose (Phase 1, cohort 1) one patient had grade 1 and one patient grade 4, and in the 50 mg BID/100 mg QD arm (Phase 1, cohort 2) one patient had grade 3 and two patients had grade 2 DS. The grade 4 event of DS in the 70 mg BID cohort, occurred during cycle 1 and led to treatment discontinuation. Study treatment was continued in the other patient on 70 mg BID with grade 1 DS with resolution of DS after DS-1594b interruption of 5 days, during cycle 1. The patient received 3 further cycles without DS recurrence. In the 50 mg BID/100 mg QD arm the patient with grade 3 DS had treatment dose reduction to 50 mg QD with resolution of DS. One patient with grade 2 DS during cycle 1 in this cohort had treatment interruption for 8 days and dose reduction to 50 mg/day with resolution of DS; subsequently cycle 2 was started at 100 mg QD without DS recurrence. The third patient (with possible grade 2 DS) died on day 3 from interventricular bleeding, considered unrelated to therapy. This patient had received only 2 days of DS1594b. Three of the 5 patients (60%) with DS had a *KMT2A*-r; the total DS rate among target population of *KMT2A*r R/R AML patients was 3/8 (37.5%). In the 7 patients treated on the lead-in dosing cohorts (cohort 3–5), 4 had a *KMT2A-*r and no DS events were observed.

Three patients died on study, all considered unrelated to the study therapy: one patient from progressive leukemia and intracranial hemorrhage, one patient from infections and retroperitoneal hemorrhage with refractory leukemia, and a third patient from infections with refractory leukemia. Among the 12 patients in whom study therapy was discontinued for lack of response and the two patients in whom study therapy was discontinued because of toxicity (one grade 4 pericarditis and one DS), none of them received any further leukemia directed therapy before their death. The 4-week and 8-week mortality rate was 11.8% and 29.4% respectively.

### Treatment efficacy

**Fig. 2:**
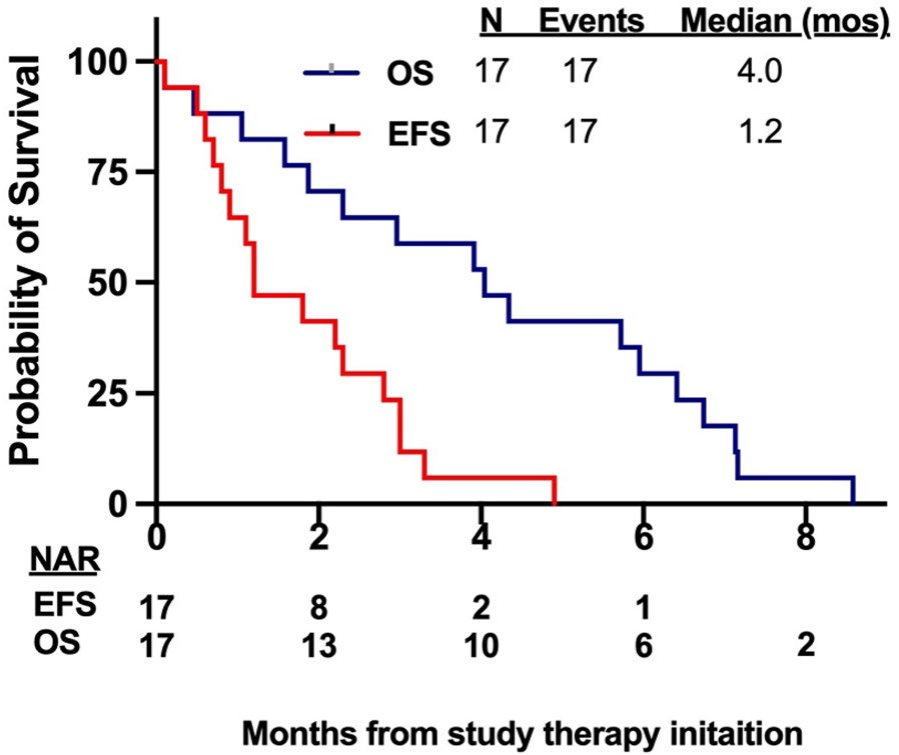
Survival outcomes. EFS, event free survival; OS, overall survival; mos, months

No patient achieved a protocol defined response. The study was terminated early before enrollment completed in cohorts with lead-in dosing up to the anticipated pharmacologically active dose, because of lack of efficacy at studied doses, supporting company portfolio reprioritization, and concurrent competing trials including numerous other menin inhibitor trials. Among the 3 patients who died on the study, 2 patients died before undergoing a response assessment BM. No patient achieved transfusion independence. Three patients had 25–50% blast reduction (70% to 38%, 52% to 32% and 60% to 35%), all after one cycle of therapy. Another patient had > 50% blast reduction (38% to 18%) after one cycle of therapy. All these 4 patients had AML and 2 of them had a *KMT2A-*r. At the time of data cutoff, no patient is on study, and no patients are alive. The median time on the study was 1.2 months (range 0.1–4.9 months). The median EFS and OS were 1.2 months (95% CI 0.7–2.8 months) and 4.0 months (95% CI 1.6–6.4 months) respectively **(**Fig. 2).

### Pharmacokinetic assessments

**Fig. 3:**
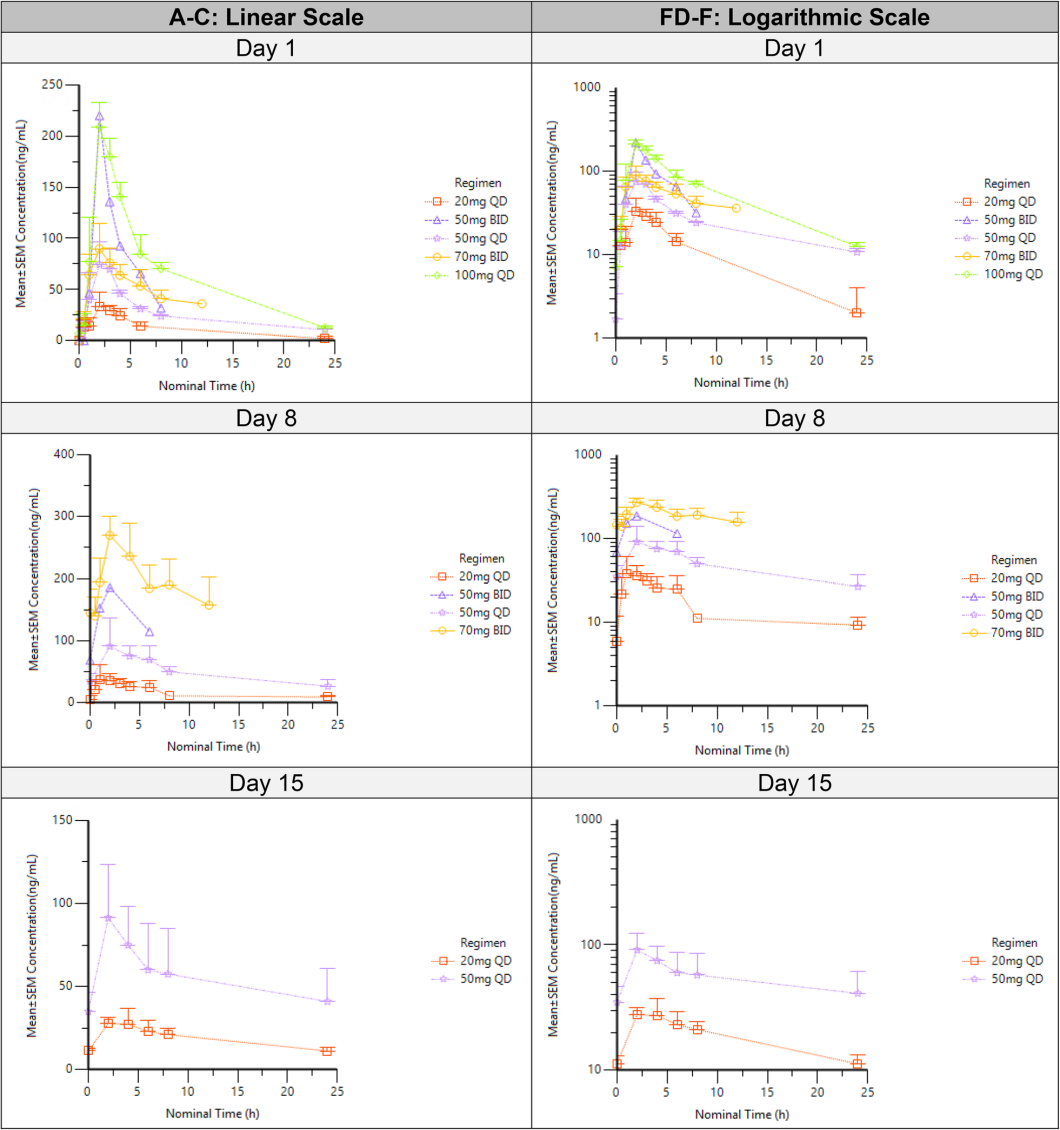
Mean±SEM DS-1594 Concentration-Time Profile by Day. 3** A-C. **Linear Scale; 3** D-F.**Logarithmic scale. QD, once daily; BID, twice daily; h, hours

The mean plasma concentration versus time plots after DS-1594b administration for cycle 1 by cohort and by day are shown in Figure S3 (linear and semi-log) and Fig. 3 (linear and semi-log) respectively. The summary pharmacokinetic parameters of DS-1594 on Day 1, 8 and 15 for all dosing regimen (20 mg QD, 50 mg BID, 50 mg QD, 70 mg BID, 100 mg QD) are tabulated in Table 4.

**Table 4:** Summary of DS-1594 Pharmacokinetic parameters

Cohort	20 mg QD			50 mg BID			50 mg QD			70 mg BID			100 mg QD		
Day	**1**	**8**	**15**	**1**	**8**	**15**	**1**	**8**	**15**	**1**	**8**	**15**	**1**	**8**	**15**
AUC_12h (h*ng/mL)	222.86 (49.95,2)	273.50 (73.10,2)	255.42 (38.31,3)	814.79 (NC,1)	NC	NC	427.22 (3.90,2)	693.16 (45.78,3)	710.93 (87.98,2)	584.45 (22.58,4)	2449.82 (33.31,3)	NC	1080.36 (7.96,3)	NC	NC
AUC_24h (h*ng/mL)	297.52 (65.08,2)	428.44 (70.35,2)	425.96 (36.54,3)	857.13 (NC,1)	NC	NC	615.55 (2.11,2)	1078.84 (45.98,3)	1227.59 (99.13,2)	715.93 (23.90,4)	3828.52 (30.60,3)	NC	1342.65 (17.55,3)	NC	NC
AUCtau (h*ng/mL)	297.52 (65.08,2)	428.44 (70.35,2)	425.96 (36.54,3)	814.79 (NC,1)	NC	NC	615.55 (2.11,2)	1078.84 (45.98,3)	1227.59 (99.13,2)	584.45 (22.58,4)	2449.82 (33.31,3)	NC	1342.65 (17.55,3)	NC	NC
AUCinf (h*ng/mL)	536.31 (NC,1)	NC	NC	857.80 (NC,1)	NC	NC	NC	NC	NC	NC	NC	NC	1578.44 (14.48,2)	NC	NC
Cmax (ng/mL)	37.12 (44.46,3)	38.69 (71.99,2)	30.39 (43.36,3)	220.00 (NC,1)	186.00 (NC,1)	NC	66.57 (61.44,3)	99.56 (53.05,3)	81.11 (64.09,3)	104.04 (21.40,4)	271.80 (27.80,4)	NC	221.02 (11.44,3)	NC	NC
Tmax (h)	1.85 (1–3,3)	1.96 (0.9–3.9,2)	2.43 (2.2–4.1,3)	2.02 (NC,1)	2.15 (NC,1)	NC	2.10 (1–6.1.1,3)	2.18 (2–4.1.1,3)	2.23 (2–2.3.3,3)	2.18 (1–4,4)	2.27 (2–4.1.1,4)	NC	2.12 (2–3.1.1,3)	NC	NC
Half-life (hr)	5.28 (77.99,2)	14.12 (23.54,2)	18.30 (15.88,3)	2.58 (NC,1)	NC	NC	13.61 (21.28,2)	12.76 (20.52,3)	58.30 (263.2,2)	4.82 (23.97,4)	14.51 (17.69,3)	NC	4.75 (61.03,3)	NC	NC
CL/F (L/h)	44.16 (NC,1)	NC	NC	61.37 (NC,1)	NC	NC	NC	NC	NC	NC	NC	NC	68.60 (14.23,2)	NC	NC
Vz/F (L)	547.54 (NC,1)	NC	NC	228.19 (NC,1)	NC	NC	NC	NC	NC	NC	NC	NC	649.07 (7.18,2)	NC	NC
AR_AUC12h^#^	-	1.40 (NC,1)	1.40 (26.90,2)	-	NC	NC	-	1.84 (62.22,2)	1.66 (94.07,2)	-	4.38 (21.60,3)	NC	-	NC	NC
AR_AUC24h^#^	-	1.48 (NC,1)	1.74 (45.20,2)	-	NC	NC	-	2.02 (57.30,2)	1.99 (102.7,2)	-	5.60 (28.77,3)	NC	-	NC	NC

All parameters are reported as geometric mean values (CV% geometric mean, N) except Tmax

Tmax is reported as Median (Min-Max, N)

NC = not calculable; ^#^Accumulation Ratio = AUC_0 − 12 h or 0−24 h, Day 8 or 15_/AUC_0 − 12 h or 0−24 h, Day 1_

After single dose (Cycle 1 Day 1), DS-1594 was absorbed with a median T_max_ from 1.85 to 2.18 h and then eliminated with a geometric mean terminal half-life from 2.58 to 13.61 h across dose levels. Geometric mean C_max_ (ng/mL) appeared to increase with dose when Day 1 values (37.12, 66.57, 221.02) were compared across 20 mg QD and 50 mg QD and 100 mg QD dosing regimen. Similarly, increased geometric mean AUC_24h_ (h*ng/mL) values with dose (297.52, 615.55 and 1342.65) on Day 1 in these cohorts suggest increase in total exposure with increasing dose. Geometric C_max_ (ng/mL) values of 220 (*N* = 1) and 104.04 (*N* = 4) and AUC_12h_ (h*ng/mL) values of 814.79 (*N* = 1) and 584.45 (*N* = 4) were observed on Day 1 following 50 mg BID and 70 mg BID administration, respectively.

After multiple dose administration (Cycle 1, Day 8 and Day 15), DS-1594 median Tmax was 1.96–2.43 h across dose levels, which is similar to single dose (Table 4). Total exposure (AUC_24h_ h*ng/mL) was similar between Day 15 and Day 8 for both 20 mg QD (425.96 vs. 428.44) and 50 mg QD (1227.59 vs. 1078.84) and comparable trends were also observed for C_max_ ng/mL (20 mg QD: 30.39 vs. 38.69, 50 mg QD: 81.11 vs. 99.56), suggesting DS-1594 reached the steady-state by Day 8 at least in 20 mg QD and 50 mg QD cohorts. AUC_24h_ based accumulation ratios (AR) were comparable between Day 15 and Day 8 (1.74 vs. 1.48 for 20 mg QD, 1.99 vs. 2.02 for 50 mg QD), suggesting moderate accumulation in these two cohorts and further support achieving stead-state by Day 8. AR values were not calculable for 50 mg BID dosing with the available data. Interestingly approximately four-fold accumulation (AR 4.38 based on AUC_12h_) was noticed for Day 8 compared to Day 1 in the 70 mg BID cohort (Table 4). BID dosing appears to have roughly twice the accumulation of QD dosing on Day 8 (70 mg BID AR 4.38 vs. 50 mg QD AR 1.84). In summary, DS-1594 reached maximum concentration approximately in 2 h with total exposure appears to increase with increasing dose. DS-1594b administration reached stead-state by Cycle 1 Day 8. Accumulation was approximately 2- and 4-fold by Day 8 with QD and BID dosing, respectively.

## Discussion

In this phase 1/2 single center open label clinical trial of a novel menin inhibitor, the agent was associated with clear evidence of DS which was manageable with no DS related mortality, The DS risk was further abated by implementing a lead-in step wise dosing strategy. Other AEs were as expected in patients with multiple treated R/R leukemias. The study was terminated early before the lead-in phases were completed because of lack of efficacy with DS-1594b (no responses) resulting in slow enrollment given competing trials with menin inhibitors.

While several menin inhibitors are in different phases of development with revumenib already US FDA approved in R/R *KMT2A*-r acute leukemias and more recently *NPM1*^mut^ AML, ongoing clinical benefits with these agents as monotherapy will be important to evaluate and may turn out to be possibly challenging. With respect to revumenib, the median DOR was about 6 months in patients with *KMT2A*-r acute leukemias who achieved a CR/CRh suggesting that transition to HSCT in remission should remain the goal for most patients and is likely needed to achieve long-term survival [7]. Similarly, in the KOMET trial with ziftomenib, no significant responses were observed in the dose finding phase while CR/CRh rate of 25% was achieved with treatment at RP2D (600 mg/day) in the phase 1b^11^. Best responses were achieved in patients with R/R *NPM1*^mut^ AML (CR/CRh rate of 35% at 600 mg dose). Notably, 5/7 patients with *NPM1*^mut^ AML treated at 600 mg dose and previously treated with venetoclax achieved a CR/CRh. However, the median DOR was again relatively limited at 6.6 months. Also, a higher rate of serious DS was seen in patients with *KMT2A*-r in both phase 1a and 1b leading to halting of further enrollment and dose escalation in patients with *KMT2A*-r in phase 1b.

DS is an important class wide AE to be recognized in patients being treated with menin inhibitors although the frequency and severity of DS may be different between different menin inhibitors and disease genotypes [21]. The present clinical trial had specific DS mitigation measures. Despite that, 5 patients had DS, for one of whom (grade 4) further treatment had to be discontinued and for another 2 patients, treatment had to be interrupted and led to alteration in dosing strategies. DS with menin inhibitors is a class effect with overall rates of about 28% with revumenib in the AUGMENT-101 study (16% grade 3) and 21% all-grade DS with ziftomenib in the KOMET study including phase 1a and 1b (*n* = 83), of which 14% were grade 3. Notably the grade 4 DS occurred at 1000 mg ziftomenib in the phase 1a which prevented further development at this dose level, and a further grade 5 DS occurred at 200 mg dose in a patient with *KMT2A*-r in phase 1b, because of which further *KMT2A*-r patients were not enrolled.

Despite the early termination of our clinical trial, the evidence of DS in 5 patients is likely suggestive of the functional on-target engagement of the drug. The PK studies showed peak concentration within 2 h of drug exposure and steady state level within a week of therapy with higher (4-fold observed versus 2-fold predicted) cumulative drug accumulation by Day 8 with the BID dosing. This led to an alteration of the dosing strategy and inclusion of QD dosing in cohort 2 to see if this would mitigate the cumulative drug accumulation resulting in more consistent and stable drug levels that could hopefully prevent drug interruption and AEs. As a note, the reason for higher than anticipated accumulation of DS-1594 compared to prediction from preclinical info is not fully elucidated but postulated etiologies included saturable absorption, time-dependent inhibition (TDI) of enzyme at gut or hepatic level. Correlative analysis looking at menin binding and depletion of menin activity was not part of this study. Additionally, the inclusion of patients without *KMT2A*-r or *NPM1* mutation in this study could have limited the scope of the drug to have shown its efficacy. Recent preclinical study in MV4;11 cells harboring *KMT2A*-r with CRISPR-Cas9 induced menin mutations (except M327) showed that DS1549b to have slightly higher IC_50_ than ziftomenib (22.2 nM) while bleximenib was more sensitive (3.3 nM) [22].

Several menin inhibitors are now in active development and some agents are already being evaluated in combination with other agents [23]. Revumenib has shown promising response rates in combination with oral decitabine-cedazuridine and venetoclax (SAVE regimen) in adults with R/R *NPM1*^mut^, *NUP98*-r, *KMT2A*-r AML [8]; ORR was 82% and CR/CRh rate 48%, 39% were consolidated with an HSCT and median duration of CR/CRh was not achieved with about 9 months median follow-up. Enzomenib as a single-agent showed impressive ORRs of 65% and 59% for *KMT2A*-r and *NPM1*^mut^ R/R AML (at the effective 200 mg or 300 mg BID doses) with CR + CRh rates of 30% and 47% respectively^12^. All grade DS was seen in 10.7% patients with Grade 3 or higher DS in only about 7%. Enzomenib, Ziftomenib and bleximenib are also being studied as part of combination therapy in newly diagnosed and R/R menin dependent leukemias and these studies will hopefully further improve the outcomes of these high-risk leukemias compared to the available current standard of care therapy [1, 4]. These drugs are also being evaluated as maintenance strategies post-consolidation and post-HSCT, as well as in some settings for MRD eradication. Given the demonstrated quick evolution of on target *MEN1* mutations in up to 30–35% of patients that likely lead to treatment resistance to revumenib, the only US FDA approved agent, evaluating and developing novel menin inhibitors with potentially differential efficacy, safety, and resistance profiles remains highly pertinent and important for the field.

In conclusion this first in human phase 1/2 clinical trial, DS1594b menin inhibitor showed DS in 29.4% patients with one grade 4 AE and no deaths directly related to the study drug. The drug as monotherapy did not lead to any responses though there were reductions in BM blast percentage. The study was terminated early due to lack of efficacy at studied dose levels and competing trials including competing menin inhibitors with demonstrated efficacy.

## Supplementary Information

Supplementary Material 1

Supplementary Material 2

## Data Availability

Data available from corresponding author on reasonable request.
