# Supplementary material for: A phase 1/2 study of DS-1594 menin inhibitor in relapsed/refractory acute leukemias
Source: J Hematol Oncol. 2025 Nov 27;18:108. doi: 10.1186/s13045-025-01757-4 (PMC12661886; doi:10.1186/s13045-025-01757-4)
Supplement: Supplementary file 1 — Supplementary Material 1 [file 13045_2025_1757_MOESM1_ESM.pdf]

MD Anderson IND Sponsor Cover Sheet	
**Protocol ID**	2020-0946
**Protocol Title**	An Open-label Phase 1/2 Multi-Arm Study of DS-1594b as a Single-Agent and in Combination with Azacitidine and Venetoclax or mini-HCVD for the Treatment of Patients with Acute Myeloid Leukemia (AML) and Acute Lymphoblastic Leukemia (ALL)
**Protocol Phase**	I/II
**Protocol Version**	8.0
**Version Date**	14 Dec 2021
**Protocol PI**	Naval Daver, MD
**Department**	Leukemia
**IND Sponsor**	MD Anderson Cancer Center
**IND #**	154785

An Open-label Phase 1/2 Multi-Arm Study of DS-1594b as a Single-Agent and in Combination with Azacitidine and Venetoclax or mini-HCVD for the Treatment of Patients with Acute Myeloid Leukemia (AML) and Acute Lymphoblastic Leukemia (ALL)

Principal Investigator: Naval Daver, MD

Co-PI: Hagop Kantarjian, MD

Sponsor: MD Anderson Cancer Center

University of Texas

MD Anderson Cancer Center

Leukemia Department, Unit 428.

Information contained in this document is proprietary to the Sponsor and to the holder of the Investigational New Drug Application. The information is provided to you in confidence which is requested under an agreed upon and signed Confidentiality and Disclosure Agreement. Do not give this document or any copy of it or reveal any proprietary information contained in it to any third party (other than those in your organization who are assisting you in this work and are bound by the Confidentiality and Disclosure Agreement) without the prior written permission of an authorized representative.

TABLE OF CONTENTS

LIST OF TABLES 7
LIST OF FIGURES 9
1. OBJECTIVES 10
2. BACKGROUND 15
2.1. Acute Myeloid Leukemia 15
2.2. Acute Lymphoblastic Leukemia 15
2.3. MLL rearrangements and NPM1 mutations in AML and ALL 15
2.4. DS-1594b 16
2.4.1. Pharmacodynamics 16
2.4.2. Safety Pharmacology and Toxicology 17
2.4.3. Benefit/Risk Assessment 18
3. STUDY DESIGN 18
3.1. Rationale for starting dose and regimen selection 19
3.1.1. Lead-in Dosing 20
3.2. Phase 1 Design 21
3.2.1. Operation Characteristics for BOIN Design 24
3.2.2. Dose-Limiting Toxicity Definition 26
3.2.3. DDI Sub-study with Anti-fungal Agents that are Strong CYP3A Inhibitors 27
3.2.3.1. Sub-Study Rationale 27
3.2.3.2. Sub-study Design 28
3.2.3.3. Sub-Study PK Evaluability Criteria 29
3.2.4. Food Effect Sub-study 29
3.2.4.1. Sub-Study Rationale 29
3.2.4.2. Sub-Study Design 29
3.2.4.3. Sub-Study PK Evaluability Criteria 30
3.3. Phase 2 Design 30
3.3.1. Rationale for Combination Cohorts (C&D) 32
4. SUBJECT SELECTION 32
4.1. Inclusion Criteria 32
4.2. Exclusion Criteria 35
5. TREATMENT PLAN 37

5.1. Phase 1 Dose Administration 37
5.1.1. Study Treatment 37
5.1.2. Dose Administration Guidance 37
5.2. Phase 2 Cohort A: R/R AML with MLL-rearrangement 39
5.2.1. Dose Administration Guidance 39
5.3. Phase 2 Cohort B: R/R AML with NPM1 mutation 39
5.3.1. Dose Administration Guidance 39
5.4. Phase 2 Cohort C: R/R AML Treated with the Combination of Azacitidine and Venetoclax with DS-1594b 39
5.4.1. Study Treatment of Study Drug and Other Combination Drugs 39
5.4.2. Venetoclax Administration 42
5.5. Phase 2 Cohort D: R/R ALL treated with mini-HCVD with DS-1594b....42
5.5.1. Treatment Overview....43
5.5.2. Mini-Hyper-CVD Regimen 44
5.5.2.1. General Considerations 44
5.5.2.2. Mini-Hyper-CVD [Odd courses 1, 3, 5, 7]: 45
5.5.2.3. Methotrexate and Cytarabine [Even courses 2, 4, 6, and 8]: 46
5.5.3. Intrathecal Treatments....46
5.5.4. Suggested Standard Dose Reductions/Modifications/Discontinuations: 47
5.5.5. Maintenance 48
5.6. Concomitant Medications 49
5.6.1. Concomitant Medications – Subject Supportive and Safety 49
5.6.2. Medications that may prolong the QTc interval 50
5.6.3. Concomitant Medications Requiring Caution....50
5.6.4. Prohibited Concomitant Medications: 50
5.6.5. Supportive Care Measures 51
6. DOSING DELAYS / MODIFICATIONS / DISCONTINUATIONS FOR POSSIBLY RELATED ADVERSE EVENTS 53
6.1. Toxicity At Least Possibly Related to Study Drugs 53
6.2. Toxicity Grading 53
6.2.1. Dose Adjustments for Hematological Adverse Events (AE) Considered at Least Possibly Related to Study Drugs 53

6.2.2. Dose Adjustments for Non-Hematologic AEs Considered at Least Possibly Attributable to Study Drugs 55
6.2.3. DS-1594b AEs of Special interest (AESI) 58
6.2.3.1. QTc Prolongation, Torsade de Pointes, and Other Ventricular Arrhythmias 58
6.2.3.2. Combined Elevations of Aminotransferases and Bilirubin 58
6.2.4. Management of AESI 59
6.2.4.1. QTc Prolongation, TdP, and Other Ventricular Arrhythmias 59
6.2.4.2. Combined Elevations of Aminotransferases and Bilirubin 59
6.3. CYP3A and P-gp Inhibitors: Dose modifications Venetoclax 60
6.4. Tumor Lysis Prophylaxis (TLS) 60
6.5. Differentiation Syndrome 60
6.6. Meals and Dietary Requirements 61
6.7. Modifications of Dose Schedules Other Than the Above Will Be Allowed Within the Following Guidelines 61
6.8. Treatment Interruptions 62
6.9. Intra-Patient Dose Escalation (IPDE) 62
7. AGENT FORMULATION AND PROCUREMENT 63
7.1. Potential Indications and Use 63
7.2. Ongoing Clinical Trials 63
7.3. Dosing and Administration 63
7.4. Packaging and Labeling 63
7.5. Study Drug Description and Preparation 63
7.6. Study Drug Storage 63
7.7. Study Drug Accountability 63
8. SUBJECT EVALUATION 64
8.1. Schedule of Assessments 65
8.2. Screening 65
8.3. Evaluation During Treatment 67
8.3.1. Lead-In Period (7-day step dosing intervals) 67
8.3.2. Target Dose or Stable Dose after Lead-In (Regular 28-day cycles begin) 67
8.4. PK Assessment 69
8.4.1. PK Blood Sample Collection and Handling 70

8.4.2. Analytical Methods 81
8.5. Cardiac Assessment (Electrocardiograms) 81
8.6. Correlative Studies (Mandatory for Phase 1) 86
8.7. Outside Physician Participation During Treatment 89
8.8. CRITERIA FOR RESPONSE: 90
8.8.1. Response Criteria for AML 90
8.8.2. Response Criteria for ALL 92
8.8.3. Transfusion Independence (TI) 92
8.8.4. Relapse Definition (AML and ALL) 92
8.9. DISCONTINUATION OF TREATMENT 93
8.9.1. Discontinuation Criteria for Individual Subjects 93
8.9.2. Study Stopping Rules 94
8.9.3. Protocol Violations and Deviations 94
8.10. ADVERSE EVENT MONITORING, RECORDING, AND REPORTING 94
8.10.1. Definitions and Procedures 94
8.10.2. Serious Adverse Events (SAEs) 97
Serious Adverse Event (SAE) Reporting Requirements for M D Anderson Sponsor Single Site IND Protocols 97
Adverse Events of Special Interest (AESI) 98
8.10.3. Pregnancy 98
8.10.4. Investigator Communications with MDACC 98
8.10.5. MDACC Reporting to Daiichi Sankyo, Inc. 99
9. STATISTICAL CONSIDERATIONS 99
9.1. Phase 1 99
9.2. Phase 2 100
9.3. Futility and Toxicity Monitoring 100
9.3.1. IND Office Futility and Safety Monitoring 101
9.4. Statistical Analysis Plan 103
9.4.1. Analysis Sets 103
9.4.1.1. Efficacy Analysis Set 103
9.4.1.2. Safety Analysis Set 103
9.4.1.3. DLT Evaluable Set 103

9.4.1.4. PK Analysis Set 103
9.4.2. Analysis Convention 104
9.4.3. Analysis of Subject Disposition, Demography and Baseline Characteristics, and Medical History 104
9.4.4. Analysis of Exposure of Study Drug 104
9.4.5. Analysis of Prior and Concomitant Medications 105
9.4.6. Efficacy Analyses 105
9.4.7. Safety Analyses 106
9.4.7.1. Dose-Limiting Toxicities 106
9.4.7.2. Adverse Events 106
9.4.7.3. Vital Signs 108
9.4.7.4. ECG 108
9.4.8. Dosing and Extent of Exposure 109
9.4.9. PK Analyses 110
9.4.9.1. Food Effect Sub-Study 110
9.4.9.2. DDI Sub-Study 110
9.4.10. PK/PD Analyses 111
10. APPENDICES 112
11. REFERENCES 140

LIST OF TABLES

Table 1: Primary Objectives and Their Endpoints and Category 10
Table 2: Secondary Objectives and Their Endpoints and Category 11
Table 3: Exploratory Objectives and Their Endpoints and Category 12
Table 4: Sample Lead-In Doses to Target Doses (QD Dosing) 21
Table 5: Dose escalation/de-escalation rule for the BOIN design 23
Table 6: Operating Characteristics of the BOIN Design 25
Table 7: Dose Levels for Azacitidine, Venetoclax, and DS-1594b 40
Table 8: Dose levels for mini-HCVD, and DS-1594b 43
Table 9: Maintenance Chemotherapy Dose Adjustments 49
Table 10: Recommendations for the use of concomitant medications and therapies 51

Table 11: DS-1594b Dose Adjustments for Neutropenia At Least Possibly Related to Study Drug 54

Table 12: Dose Adjustments of Azacitidine, Venetoclax, and/or DS-1594b for Non-Hematologic Possibly Related AEs, Clinically Significant in the Opinion of the Investigator 56

Table 13: Dose Adjustment for QTc Interval Prolongation 57

Table 14: Dose Modification for Clinically Suspected Acute Differentiation Syndrome 57

Table 15: DS-1594b PK Blood Collection for Twice a Day (BID) Dosing During Phase 1 [Excluding Subjects Enrolled in DDI and Food Effect Sub-Studies and NOT Applicable for Cohorts with Lead-in Period] Dose Escalation 71

Table 16: DS-1594b PK Blood Collection for Daily (QD) Dosing During Phase 1 [Excluding Subjects Enrolled in DDI and Food Effect Sub-Studies and NOT Applicable for Cohorts with Lead-in period] Dose Escalation 72

Table 17: DS-1594b PK Blood Collection for Daily (QD) Dosing with Lead-in Period (1-Week Interval per Each Step-up Dose Level) During Phase 1 [Excluding Subjects Enrolled in DDI and Food Effect Sub-Studies] Dose Escalation 74

Table 18: DS-1594b PK Blood Collection During Phase 2 Dose Expansion 76

Table 19: DS-1594b PK Blood Collection for DDI Sub-Study with Antifungal Agents During Phase 1 After MTD Determination (N ~ 12) 77

Table 20: DS-1594b PK Blood Collection for DDI Sub-Study with Antifungal Agents During Phase 1 Before MTD Determination 78

Table 21: DS-1594b PK Blood Collection for Food Effect Sub-Study During Phase 1 After MTD Determination (N ~ 6) 79

Table 22: ECG Collection During Phase 1 Escalation (Excluding Cohorts with Lead-in Period) 81

Table 23: ECG Collection During Phase 1 Escalation For Cohorts with Lead-in Period 83

Table 24: ECG Collection During Phase 2 Dose Expansion 85

Table 25: Stopping Boundaries for Toxicity and Futility Monitoring in Each of Cohorts A-D 101

Table 26: Operating Characteristics for Futility Monitoring in each of Cohorts A-D 101

Table 27: Operating Characteristics for Toxicity Monitoring in each of Cohorts A-D 102

Table 28: Overall early stopping probabilities for Futility and Toxicity Monitoring in each of Cohorts A-D 102

Table 29: 95% Credible Intervals Under Various Scenarios with Different Numbers of Responses 103

Table 30: AESI Categories and SMQ Search Criteria 107

Table 31: List of Prohibited QT Prolongation Drugs 112
Table 32: Prohibited Medications That Are Strong Inhibitors or Inducers of CYP3A, or CYP3A/P-Gp and CYP2C19 Substrates with Narrow Therapeutic Indices 113
Table 33: List of medications to be used with caution 114
Table 34: Additional Prohibited Medications for Subjects Enrolled in DDI and Food Effect Sub-Studies 116
Table 35: Phase 1 Lead-in Period Schedule of Events (QD Dosing) 117
Table 36: Phase 1 Dosing at the Target Dose Level 120
Table 37: Phase 2 Cohorts A & B Schedule of Events 127
Table 38: Phase 2 Cohort C Schedule of Events 131
Table 39: Phase 2 Cohort D Schedule of Events 135

LIST OF FIGURES

Figure 1: Study Schema with Target Doses in Phase 1 19
Figure 2: Sample Dosing Schematic for Lead-in to the Target Dose for the Cohort 20
Figure 3: Flowchart for Trial Conduct Using the BOIN Design 24

1. OBJECTIVES

The Primary, Secondary, and Exploratory Objectives are shown in tabular format in the following three tables. See Figure 1 for this Phase 1 / 2 Study Diagram.

Table 1: Primary Objectives and Their Endpoints and Category

Primary Objectives	Endpoints	Category
**Phase 1 only:**<br>To determine the safety, tolerability, Maximum Tolerable Dose (MTD), and Recommended Phase 2 Dose (RP2D) of DS-1594b in subjects with relapsed/refractory (R/R) Acute Myeloid Leukemia (AML) and Acute Lymphoblastic Leukemia (ALL).	MTD;<br>RP2D;<br>Safety Profile of DS-1594b (e.g., adverse events, ECGs, vital signs, clinical laboratory tests [including chemistry, hematology, coagulation], physical examinations)	Safety
**Phase 2 only (Cohort A):**<br>To evaluate the safety, tolerability, and efficacy defined as Complete Remission (CR) and Complete Remission with Partial Hematologic Recovery (CRh) rate of DS-1594b in subjects with R/R AML with Mixed-Lineage Leukemia rearrangement (MLLr).	CR/CRh rate with DS-1594b monotherapy in R/R AML with MLLr;<br>Safety Profile of DS-1594b	Efficacy<br>Safety
**Phase 2 only (Cohort B):**<br>To evaluate the safety, tolerability, and efficacy defined as CR+CRh rate of DS-1594b in subjects with R/R AML with a Nucleophosmin 1 mutation (NPM1m).	CR/CRh rate with DS-1594b monotherapy in R/R AML with NPM1m;<br>Safety Profile of DS-1594b	Efficacy<br>Safety
**Phase 2 only (Cohort C):**<br>To evaluate the safety, tolerability, and efficacy defined as CR+CRh rate of DS-1594b in combination with azacitidine and venetoclax in R/R MLLr or R/R NPM1m AML.	CR/CRh rate with DS-1594b monotherapy in R/R AML with MLLr;<br>Safety Profile of DS-1594b in combination with azacitidine and venetoclax	Efficacy<br>Safety
**Phase 2 only (Cohort D):**<br>To evaluate the safety, tolerability, and efficacy defined as CR+ Complete Remission with Incomplete Hematologic Recovery (CRi) rate of DS-1594b in combination with mini-HCVD in R/R ALL with MLLr.	CR/CRi rate with DS-1594b in combination with mini-HCVD in R/R ALL with MLLr;<br>Safety Profile of DS-1594b in combination with mini-HCVD	Efficacy<br>Safety

Response definitions Section 8.8

Table 2: Secondary Objectives and Their Endpoints and Category

Secondary Objectives	Endpoints	Category
**Phase 1 only:**<br>To evaluate the combined complete remission (CR) and CR with partial hematologic recovery (CRh) rate within 3 months of therapy initiation with DS-1594b in subjects with R/R AML or R/R ALL.	CR/CRh rate in Phase 1	Efficacy
**Phases 1 and 2:**<br>To assess the composite CR (CRc) rate defined as CR + complete remission with incomplete blood count recovery (CRi), morphologic leukemia free survival (MLFS), partial remission (PR), and overall response rate of subjects with R/R AML and R/R ALL treated on single-agent or combinations of DS-1594b.	CRc rate<br>MLFS rate<br>PR rate<br>ORR (CR + CRi + MLFS + PR) rate	Efficacy
**Phases 1 and 2:**<br>To determine the:<br>• duration of response (DOR),<br>• time to first response, time to best response,<br>• rate of durable transfusion independence (TI),<br>• event-free survival (EFS),<br>• overall survival (OS),<br>• measurable residual disease (MRD) by multiparameter flow cytometry at first response and best response,<br>• 4- and 8-week mortality, and<br>• number of subjects able to proceed to hematopoietic stem cell transplantation (HSCT) without additional AML therapy and median duration to HSCT from the initiation of single-agent or combinations of DS-	• DOR,<br>• time to first response, time to best response,<br>• TI rate,<br>• EFS,<br>• OS,<br>• MRD rate by multiparameter flow cytometry at first response and best response,<br>• 4- and 8-week mortality<br>• number of subjects able to proceed to HSCT without additional AML therapy and median duration to HSCT from the initiation of single-agent or combinations of DS-1594b in subjects with R/R AML and R/R ALL	Efficacy

Secondary Objectives	Endpoints	Category
1594b in subjects with RR AML and R/R ALL		
**Phases 1 and 2:** To characterize single and multiple-dose pharmacokinetics (PK) of DS-1594b and its metabolites	Cmax, Ctrough, Tmax, Tlast, AUClast, AUCtau, AUCinf, Kel, t1/2, CL/F, Vz/F, AR	PK
**Phase 1 only:** In a subset of subjects, to evaluate the effect of multiple doses of antifungal agents that are strong CYP3A inhibitors on the PK of DS-1594b	Cmax, Ctrough, Tmax, Tlast, AUClast, AUCtau, AUCinf, Kel, t1/2, CL/F, Vz/F	PK
**Phases 1 only:** In a subset of subjects, to evaluate the relative exposures of DS-1594b when administered with a standard meal compared to fasted conditions	Cmax, Ctrough, Tmax, Tlast, AUClast, AUCtau, AUCinf, Kel, t1/2, CL/F, Vz/F	PK

Table 3: Exploratory Objectives and Their Endpoints and Category

Exploratory Objectives	Endpoints	Category
**Phases 1 and 2:** To investigate correlations of clinical benefit to single-agent or combinations of DS-1594b with a pre- therapy, on-therapy, and progression using an appropriate NGS panel of gene mutations in AML and ALL, routine cytogenetics/karyotyping, fusion panels, immunophenotyping, and fluorescence in situ hybridization (FISH), when applicable.	Not applicable	PD/Biomarker

Exploratory Objectives	Endpoints	Category
**Phases 1 and 2:**<br>To investigate possible relationships between response and non-response to single-agent or combinations of DS-1594b with pretherapy, on-therapy, and progression gene expression signatures (e.g., MEIS1, MEF2c or HOXA).	Not applicable	PD/Biomarker
**Phases 1 and 2:**<br>To investigate the characterization of genetic heterogeneity in AML or ALL tumor cell populations that are longitudinally collected, by performing targeted single-cell sequencing using a novel microfluidic approach that barcodes amplified genomic DNA from thousands of individual leukemia cells confined to droplets (single cell sequencing). Targeted single-cell sequencing is able to sensitively identify cells harboring pathogenic mutations during complete remission and uncover complex clonal evolution within AML or ALL tumors that are not observable with bulk sequencing.	Not applicable	PD/Biomarker
**Phases 1 and 2:**<br>To identify individual cell populations (AML/ALL blasts, T-cells – both bulk and T-cell subsets and coreceptor/ligand expression, macrophages and their coreceptor/ligands) and how their signaling state in disease relates to clinical outcomes we will perform CyTOF (mass cytometry) using a customized CyTOF panel specifically developed for this study on subjects' bone marrow samples and peripheral blood at diagnosis, remission and relapse and potentially other time-points on study.	Not applicable	PD/Biomarker

Exploratory Objectives	Endpoints	Category
**Phases 1 and 2:**<br>To store and/or analyze surplus blood or tissue including bone marrow, if available, for potential future companion diagnostic development or exploratory research into factors that may influence development of AML/ALL and/or response to therapies (where response is defined broadly to include efficacy, tolerability or safety).	Not applicable	PD/Biomarker
**Phases 1 and 2:**<br>To assess the relationship between DS-1594b PK and PD biomarker, safety (e.g., QT prolongation) and efficacy endpoints.	PK parameters vs. PD, AEs, efficacy	PK

2. BACKGROUND

2.1. Acute Myeloid Leukemia

Acute Myeloid Leukemia (AML) is a hematologic neoplasm characterized by the proliferation of poorly differentiated myeloid progenitor cells. The disease is particularly difficult to treat in older adults who account for the majority of subjects; thus, the 5-year overall survival is only approximately 27% in newly diagnosed AML[1] . In recent years, multiple studies have characterized distinct molecular subgroups of AML, leading to the discovery of certain disease-associated mutations that are now being used to classify disease, dictate prognosis and, in some cases, guide therapy[2,3].

Approximately, 30 to 40% of adults with AML fail to achieve CR with 1 or 2 cycles of induction chemotherapy and are deemed primary refractory. The outcome of subjects with AML who are refractory to induction therapy are dismal, with low response rates (CR/CRi = 15 to 25%) to salvage chemotherapy and poor long-term survival[4,5,6], median OS 4-7 months . We have previously reported a dismal median OS of 3.8 months for subjects with AML who are refractory to HiDAC-containing induction therapy (defined as ≥ 1gm/m^2 cytarabine per dose[7]). Salvage therapy in such subject populations yielded a response rate of 18% and median response duration of 9 months. These results emphasize the need to explore alternate salvage regimens for subjects with relapsed/refractory AML. The development of novel and effective anti-AML agents and/or combinations is crucial to improving the outcome of AML.

2.2. Acute Lymphoblastic Leukemia

Acute Lymphoblastic leukemia (ALL) is a malignant transformation and proliferation of lymphoid progenitor cells. The incidence of ALL follows a bimodal distribution, with the first peak occurring in childhood and a second peak occurring around the age of 50[8]. The 5-year overall survival is approximately 90% in children and 30% to 40% in adults and elderly subjects. It is known that chromosomal alterations are a hallmark of ALL and include aneuploidy and recurring chromosomal translocations[9]. KMT2A rearrangements occur in about 5% of ALL and confer resistance to standard chemotherapy as well as novel agents including blinatumomab and inotuzumab.

2.3. MLL rearrangements and NPM1 mutations in AML and ALL

Two examples of mutations or cytogenetic aberrations are most relevant to our proposed study here:

The first example is the mixed-lineage leukemia (MLL) gene, also known as KMT2A, on chromosome 11q23, which is disrupted in a unique group of acute leukemias. Translocations of MLL gene, which are found in approximately 5%-10% of acute leukemia subjects, result in an aggressive subtype of leukemia, with a 5-year survival rate of only approximately 35%[10,11]. More than 80 different partner genes in these fusions have been described, although the majority of leukemias result from MLL fusions with one of about six common partner genes (AF4, AF9, ENL, AF10, ELL, PTD) and only 9 specific gene fusions account for more than 90% of all aberrant recombinations of the MLL gene[12]. MLL translocations lead to fusion of the N-terminal

fragment of MLL with 1 of the >80 protein partners, resulting in the expression of MLL fusion proteins that enhance proliferation and block hematopoietic differentiation, ultimately driving the development of leukemia. MLL fusion proteins bind to DNA/chromatin and induce leukemic transformation in hematopoietic stem and progenitor cells through transcriptional deregulation of fusion protein target genes. The best studied of the target genes include HOXA genes and their co-factors MEIS1, which have been shown to be important for leukemia development, cell proliferation, and self-renewal. Because of poor subject outcomes, MLL leukemia represents an unmet medical need, necessitating the development of new therapies.

The second example is one of the most common recurrent driver mutations in AML: mutation of the nucleophosmin 1 (NPM1) gene, which is seen in approximately 30% of cases[2]. NPM1 mutations most commonly occur in de novo AML and are associated with a normal karyotype. NPM1 mutations are associated with the upregulated expression of HOXA and MEIS1 genes as do the MLL rearrangements[13]. AML with mutated NPM1 is associated with a more favorable prognosis in the newly diagnosed setting; however, this favorable prognostic effect is lost in the presence of concomitant FLT3-ITD mutations, which are seen in 40%-50% of AML with mutated NPM1, or in a relapsed/refractory setting[14]. In addition to FLT3-ITD mutations, mutations in genes regulating DNA methylation, including DNMT3A, IDH1, and IDH2, are also commonly seen in the presence of NPM1 mutations, and these combined mutations are associated with significantly inferior overall survival[15], emphasizing the need for new therapies.

Menin is a scaffold protein encoded by the multiple endocrine neoplasia type 1 (MEN1) gene. In the nucleus, menin associates with different proteins that activate or repress the transcription of genes involved in multiple cellular processes. Among them, menin interacts with MLL. MLL has 2 fragments, MBM1 and MBM2, located within the 43-amino acid fragment at the N-terminus of MLL[16]. Because this N-terminus of MLL is retained in MLL fusion proteins, the menin-MLL interaction is preserved among wild-type MLL and all MLL fusion proteins. It has been reported that menin protein interaction with MLL fusion proteins, which leads to upregulation of HOXA and MEIS1 gene expressions, is crucial for the development of acute leukemias with MLL rearrangements[17,18]. Additionally, it has also been reported that menin protein interaction with wild-type MLL proteins, which is suggested to lead to upregulation of HOXA and MEIS1 gene expressions, is crucial for the development of other types of acute leukemias, such as those with NPM1 mutations[13]. The interaction of MLL1 fusion proteins with menin plays an important role to enhance the proliferation and block the differentiation of hematopoietic cells, ultimately leading to acute leukemia[19,20]. Therefore, inhibition of the menin-MLL interaction is expected to show efficacy against these acute leukemias. Subjects with MLL-r acute leukemia respond poorly to currently available treatments[19], emphasizing the urgent need to develop more effective therapies directly disrupting the menin-MLL1 complex.

2.4. DS-1594b

2.4.1. Pharmacodynamics

DS-1594b is the development designation of a novel, potent, orally available small molecule inhibitor of the binding of MLL1 fusion proteins and wild-type MLL1 to menin in development for the treatment of subjects with hematologic malignancies, including subjects with acute

leukemia with MLL1-r and NPM1 mutations. MLL1 is also known as KMT2A (lysine methyltransferase 2A). The nonclinical testing strategy was designed to demonstrate and characterize the mechanism of action of DS-1594b as well as to support the oral dosing regimen planned for the initial clinical studies. Nonclinical primary pharmacodynamic studies supporting development of DS-1594b were designed to provide information on its mechanism of action and potential for efficacy in the treatment of hematological tumors. In vitro studies assessed the effects of DS-1594b on the interaction between menin and MLL1, cellular growth of MV-4-11, MOLM-13, and K-562 cell lines, and expression levels of *MEIS1*, *HOXA9*, and *PBX3* genes in MV-4-11 and MOLM-13 cell lines. In vivo studies in mice evaluated the effects of DS-1594b on antitumor activity against MV-4-11 xenografts.

2.4.2. Safety Pharmacology and Toxicology

The safety pharmacology parameters were assessed in mice (CNS) and in monkeys (cardiovascular and respiratory systems). DS-1594b was administered orally by gavage in all in vivo studies. In addition, the effect of DS-1594b on the hERG potassium channel current was assessed in vitro. DS-1594b has potent activity on the hERG channel with an IC_{50} value of 0.3 µM. In an in vivo cardiovascular study using telemetered male cynomolgus monkeys, QT and QTc intervals were prolonged following a single administration of DS-1594b at ≥ 20 mg/kg in a dose dependent manner. Based on the exposure-response (ER) analysis, the plasma concentration of DS-1594a (free form of DS-1594b) to induce QTc interval prolongation by 10 milliseconds was predicted to be around 1000 ng/mL. No effect on any other cardiovascular or respiratory parameters was noted at doses up to 200 mg/kg. In the 28-day repeated dose toxicity study in monkeys, the QTc interval increased on Days 8 and 22 at ≥ 60 mg/kg/day; however, no exacerbation of the increased QTc interval was seen on Day 22 compared to that on Day 8. The anticipated human equivalent dose of the 60mg/kg/day dose in monkeys is approximately 19.2 mg/kg/day.

DS-1594b is highly bound to plasma protein (>97%) in all species. No concentration-dependency was observed across species. Cytochrome P450 (CYP) 3A4 is the primary CYP isoform involved in the metabolism of DS-1594b from the results of experiments using human CYP-expressing microsomes and human liver microsomes. DS-1594b did not inhibit CYP1A2 or CYP2B6 (IC_{50} > 50 µM). The IC_{50} values of DS-1594b were 19.6 µM for CYP2C8, 48.4 µM for CYP2C9, 2.66 µM for CYP2C19, and 27.0 µM for CYP2D6 without preincubation and comparable to those with preincubation. In addition, DS-1594b induced time-dependent inhibitory effects on CYP3A.

Results from in vitro studies using Caco-2 cell monolayer systems suggested that DS-1594b is a substrate of efflux transporters. In an exploratory PK study after oral dosing of DS-1594b at 3 mg/kg to rats, AUC increase of DS-1594b was observed by oral pretreatment of elacridar at 50 mg/kg, a P-gp inhibitor which partially inhibits BCRP. These observations indicated that DS-1594b is likely transported by P-gp, and BCRP to a lesser extent.

DS-1594b inhibited OATP1B1 (IC_{50}: 20.5 µM), OATP1B3 (IC_{50}: 32.3 µM), OCT1 (IC_{50}: 0.489 µM), OCT2 (IC_{50}: 12.8 µM), MATE1 (IC_{50}: 2.78 µM), MATE2-K (IC_{50}: 3.12 µM), P-gp (IC_{50}: 1.79 µM) and BCRP (IC_{50}: 1.94 µM) to different extent.

2.4.3. Benefit/Risk Assessment

DS-1594b is under development for the treatment of hematologic malignancies, including acute leukemias with MLL1-r or NPM1-mutations. No clinical studies have been conducted and therefore, no efficacy or safety in humans has been demonstrated.

Nonclinical studies have demonstrated the potent antitumor activity of DS-1594b in mouse models of acute leukemia with MLL1-r or NPM1-mutations. Therefore, DS-1594b is expected to demonstrate efficacy in subjects with MLL1-r or NPM1-mutated acute myeloid leukemia and acute lymphoid leukemia.

Potential risks of DS-1594b, based either on mechanism of action (i.e., differentiation syndrome), or nonclinical toxicity studies or clinical studies with other products of the same class include QT interval prolongation, gastrointestinal toxicity (i.e., nausea, vomiting), hematological (i.e., anemia, neutropenia, and thrombocytopenia), renal, hepatic, skeletal muscle, pulmonary, and cardiac toxicities should be surveilled and appropriately managed in clinical studies.

Based on the nonclinical data, the benefit-risk balance supports clinical development of DS-1594b.

Please see the Investigator Brochure for more information about DS-1594b.

3. STUDY DESIGN

This will be a Phase 1/2 study of orally administered single agent DS-1594b in subjects with R/R AML or ALL, including subjects with or without MLLr or NPM1m (Figure 1).

The Phase 1 portion of the study is open-label, non-randomized, sequential, BOIN escalation. Dose Escalation will provide the MTD and/or RP2D for the Phase 2 portion and will include two sub-studies to evaluate the drug-drug interaction potential with strong inhibitors of CYP3A and the effect of DS-1594b co-administration with food.

The Phase 2 portion is a four-cohort, open-label, non-randomized study with no crossover permitted between the cohorts to evaluate single agent DS-1594b and combinations of DS-1594b with azacitidine and venetoclax in AML subjects with MLLr or NPM1m, and with mini-HCVD in ALL subjects with MLLr.

A Safety Review Committee (SRC), comprised of selected Sponsor study team members and Investigators, will be responsible for overseeing the safety of dose escalation/de-escalation, declaring the RP2D, and recommending whether to open each expansion cohort. The SRC will review the emerging safety and PK data (if available) from each cohort to determine if dose escalation will occur.

Figure 1: Study Schema with Target Doses in Phase 1

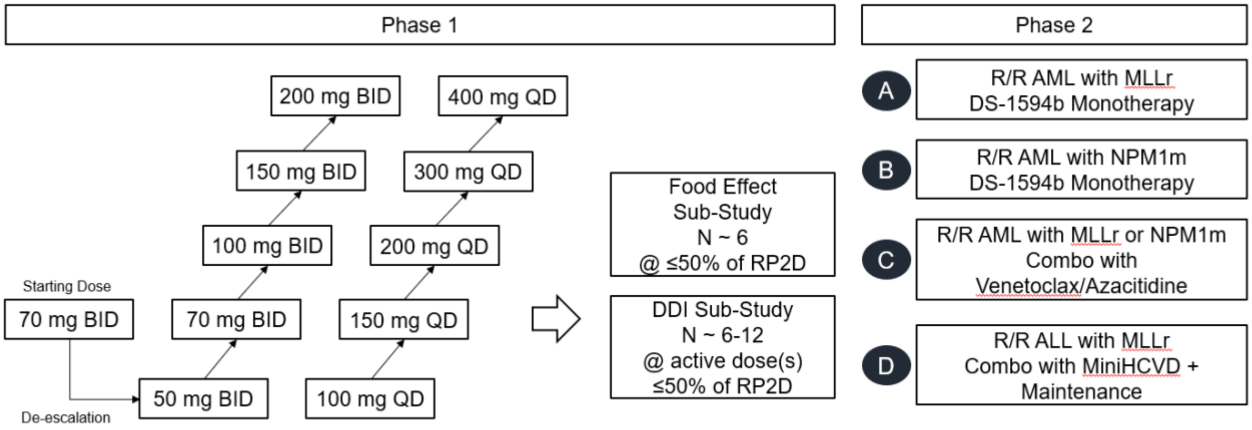

Note: The boxed doses represent the target dose for the cohorts with or without lead-in dosing. All target dose levels after the starting dose are tentative. Alternative doses or dosing regimens may be enrolled based on emerging clinical data, including further de-escalation. BID and QD cohorts may be enrolled in parallel and will be evaluated independently for DLT. Based on emerging clinical data, lead-in dosing to the target dose will be implemented to mitigate the potential clinical risks associated with rapid differentiation. See Section 3.1.1 for additional information. In Phase 2, Cohorts A and B (single agent cohorts) will enroll a maximum of 26 subjects, and Cohorts C and D (combination cohorts) will enroll a maximum of 32 subjects

3.1. Rationale for starting dose and regimen selection

The selection of the starting dose level and regimen is based on the extrapolation of data from cell line efficacy models and toxicology studies in rodents and monkeys.

The DS-1594b Severely Toxic Dose in 10% of animals (STD_{10}) in mice was greater than 300 mg/kg/day and the Highest Non-Severely Toxic Dose (HNSTD) in monkeys was 60 mg/kg/day. The human equivalent dose (HED) of 300 mg/kg/day in mice, based on allometric scaling by body surface area, is approximately 24.3 mg/kg, and using 1/10th of the HED gives a dose of 2.4 mg/kg as the recommended starting human dose for the proposed clinical study. The value of 2.4 mg/kg is lower than a starting dose (3.2 mg/kg) derived from 1/6th of the HED for the HNSTD (60 mg/kg/day) in monkeys. The estimated human systemic exposure at the starting dose exceeds the IC_{50} of DS-1594b against MV-4-11 human AML cell line (1.97 nM [1.13 ng/mL]), and inhibition of cell proliferation is expected. Therefore, the value of 2.4 mg/kg is considered the appropriate starting dose for the proposed clinical study. No supportive data are available at this stage that would suggest body size-based dosing would reduce human inter-subject variability in PK. Thus, based on an average body weight of 60 kg human, the recommended starting human dose is 140 mg/day. A dosing approach with 70 mg given twice a day will be adopted in order to reduce C_{max} while maintaining AUC compared to once-daily dosing to mitigate potential QT interval prolongation risk, given the observed QTc interval prolongation signal seen in monkeys (see Section 2.4.2). In addition, the estimated clinical half-life of DS-1594b is ~ 7 to 8 hours, which also supports a twice daily dosing regimen.

Based on emerging clinical data to assess the PK and safety profile of DS-1594b, the frequency of dosing may be adjusted (e.g., once daily [QD]), if deemed appropriate and supported by safety

data. In this case, dose escalation would follow the same rules as the BID schedule. The SRC may recommend the enrollment of simultaneous QD and BID dosing regimens or may switch from BID dosing to QD dosing.

In case two dosing regimens are enrolling simultaneously, sequentially enrolling subjects will be assigned to either regimen in an alternating method. After at least 3 subjects have completed the dose-limiting toxicity (DLT) evaluation window for a given cohort, all available clinical data (e.g., PK, safety and any other available data) will be assessed by the SRC to determine if a single dosing regimen (i.e., QD or BID) should be selected. If the SRC selects a single dosing regimen, simultaneous enrollment into both regimens will stop and the remaining subjects in dose-escalation will be enrolled in the subsequent dose level for that selected dosing regimen.

3.1.1. Lead-in Dosing

To mitigate the potential clinical risks associated with rapidly differentiating leukemic blasts, lead-in dosing will be employed to sensitize the subjects and to evaluate step-wise lead-in dosing. In the lead-in dosing period, subjects may receive one or multiple ascending lead-in doses with each dose lasting for approximately 7 days; however, these lead-in doses and the duration of each step may be altered based on tolerability and at the investigator's discretion. The starting dose for the lead-in period will be 20 mg. Upon completion of the lead-in period, subjects will receive their target cohort dose of DS-1594b, as shown in Figure 2. A similar strategy was implemented during the development of venetoclax to mitigate the risks of tumor lysis syndrome.

Figure 2: Sample Dosing Schematic for Lead-in to the Target Dose for the Cohort

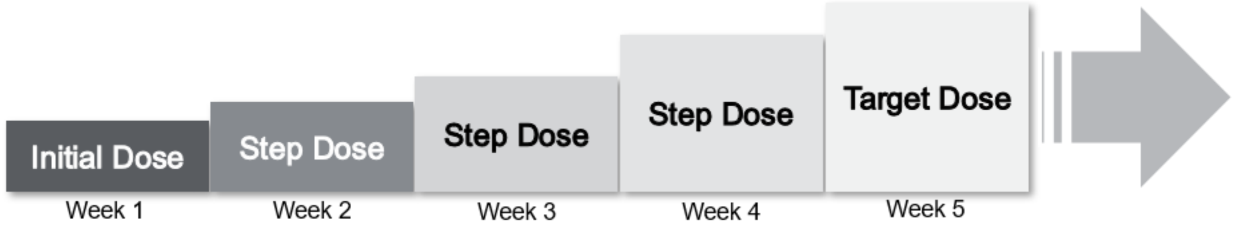

From: Roberts AW, Davids MS, Pagel JM, et al. Targeting BCL2 with Venetoclax in Relapsed Chronic Lymphocytic Leukemia. N Engl J Med 2016;374:311-22.

For all dose escalations, all laboratory values for subjects must be reviewed in real time by the investigator as they become available and prior to the subject's next dose level to ensure appropriate subject management. Based upon the results, the subject may continue dosing, may need to hold dose until resolution, may require hospitalization for further monitoring, or may need additional post-dose laboratory checks.

Sample lead-in doses to the target doses are shown in Table 4. Following the lead-in period, subjects will receive the target dose in continuous 28-day cycles. Please note that these lead-in doses and their respective durations may be altered based on subject's tolerability and at

investigator's discretion. Additionally, the Safety Review Committee may recommend enrolling cohorts without lead-in dosing.

Table 4: Sample Lead-In Doses to Target Doses (QD Dosing)

**Cohort**	**Initial Dose (mg)** [Lead-in Period]	**Step 2 (mg)** [Lead-in Period]	**Step 3 (mg)** [Lead-in Period]	**Step 4 (mg)** [Lead-in Period]	**Target Dose (mg)** [Treatment Cycle 1 Day 1]	**DLT period** (lead-in period plus 28-days at target dose)
4	20	-	-	-	50	35
5	20	50	-	-	100	42
6	20	50	100	-	150	49
7	20	50	100	150	200	56
8	20	50	100	200	300	56
9	20	50	100	200	400	56

Note: Doses shown in this table are once-daily (QD) doses. Steps 2-4 may be skipped if no clinically significant laboratory or clinical symptoms of differentiation are observed in a prior dose. Alternative doses, step intervals, and interval durations may be implemented based upon emerging data. The initial duration of each step interval will be 1 week.

3.2. Phase 1 Design

The primary objective of Phase 1 is to determine the safety, Maximum Tolerated Dose (MTD), and the Recommended Phase 2 Dose (RP2D) of DS-1594b when administered as a single agent in subjects with relapsed or refractory AML or ALL, including subjects with or without MLLr or NPM1m using a Bayesian optimal interval (BOIN) design to find the MTD[34,35].

The BOIN design is implemented in a simple way similar to the traditional 3+3 design but is more flexible and possesses superior operating characteristics that are comparable to those of the more complex model-based designs, such as the continual reassessment method (CRM)[33].

At least 3 subjects must be enrolled at each target dose level. If a target dose level is found to be safe by the BOIN design we will escalate to the next higher dose level. The SRC can recommend additional enrollment of up to 9 additional subjects per target dose level for dose levels that have already been deemed to be safe with the intent to gain additional information on safety, efficacy and PK at different dose levels, and to guide selection of optimal biologically effective RP2D. These additional 9 subjects per target dose level will not be considered evaluable for DLTs. It is anticipated that approximately 18 subjects will be added in this Phase 1 expansion.

Although all R/R ALL and R/R AML subjects, irrespective of mutations, will be allowed during the Phase 1 dose-escalation, the goal is to ensure that at least 3 subjects with MLLr or NPM1m are included at target dose levels that are anticipated to be biologically active to allow for optimal evaluation of safety and efficacy in the presumed target population to guide selection of the optimal RP2D.

Treatment during Phase 1 dose escalation phase will follow continuous oral administration under a twice-daily schedule or daily schedule on a 28-day dosing cycle.

The first dose cohort will start at 70 mg twice daily, approximately every 12 hours. The target toxicity rate for the MTD is $\phi = 0.2$ and the maximum sample size for determining the MTD is 42. Each cohort will include a minimum of 3 subjects. The DLT evaluation period will include the complete lead-in dosing period plus the first 28 days of dosing at the target dose. For example, if a subject receives 20 mg QD for 7 days followed by 50 mg QD for 7 days, before receiving the target dose of 100 mg QD, the DLT period will include total of (7+7+28) or 42 days. DLTs are defined in Section 3.2.2 and only those DLTs that occur within the DLT evaluation period will be used for dose finding. As shown in Figure 3, the BOIN design uses the following rule, optimized to minimize the probability of incorrect dose assignment, to guide dose escalation/de-escalation:

- if the observed DLT rate at the current target dose is 0.157 or less, escalate the target dose to the next higher dose level;
- if the observed DLT rate at the current target dose is more than 0.238, de-escalate the dose to the next lower target dose level;
- otherwise, stay at the current target dose.

Evaluation by the abovementioned BOIN rule will be done for each target dose level separately based on the cumulative number of DLT evaluable subjects treated in that target dose level and regimen (i.e., QD or BID). In case the multiple dosing regimens are initiated simultaneously and parallel enrollment is allowed in the multiple target dosing regimens, the evaluation by the BOIN rule for each target dose level and regimen would be made separately. DLT observed after intra-patient dose escalation (IPDE) will not be considered in the dose escalation/de-escalation decision (See Section 6.9 for details about IPDE). For the purpose of overdose control, doses j and higher levels will be eliminated from further examination if $\Pr(p_j > 0.2 \mid \text{data}) > 0.95$ and at least 3 subjects have been treated at dose level j, where p_j is the true DLT rate of target dose level $j, j = 1, \cdots, 6$. This posterior probability is evaluated based on the beta-binomial model $y_j \mid p_j \sim \text{binomial}(p_j)$ with $p_j \sim \text{uniform}(0,1)$, where y_j is the number of subjects who experienced DLT at target dose level j. When the lowest dose is eliminated, stop the trial for safety. The probability cutoff 0.95 is chosen to be consistent with the common practice that when the target DLT rate <=1/6, a dose with 2/3 subjects experienced DLT is eliminated. The above dose escalation/de-escalation and elimination rule can be equivalently presented in Table 5, which will be used to conduct the trial.

The steps to implement the BOIN design are described as follows:

1. Three subjects in the first cohort are treated at dose level 1 (70 mg twice daily). Each cohort will include a minimum of 3 subjects.
2. To assign a dose to the next cohort of subjects, conduct dose escalation/de-escalation according to the rule displayed in Table 5. When using Table 5, please note the following:

   a. "Eliminate" means eliminate the current and higher doses from the trial to prevent treating any future subjects at these doses because they are overly toxic.
   b. When we eliminate a dose, automatically de-escalate the target dose to the next lower level. When the lowest dose is eliminated, stop the trial for safety. In this case, no dose should be selected as the MTD.
   c. If none of the actions (i.e., escalation, de-escalation or elimination) is triggered, treat the new subjects at the current target dose.
   d. If the current dose is the lowest dose and the rule indicates dose de-escalation, treat the new subjects at the lowest dose unless the number of DLTs reaches the elimination boundary, at which point terminate the trial for safety.
   e. If the current target dose is the highest dose and the rule indicates dose escalation, treat the new subjects at the highest target dose.
3. Repeat step 3 until the maximum sample size of 30 is reached or stop the trial if the number of subjects treated at the current dose reaches 9 and the decision according to Table 5 is to stay at the current target dose.

Table 5: Dose escalation/de-escalation rule for the BOIN design

Number of subjects treated at current target dose	3	4	5	6	7	8	9
Escalate if number of DLTs is less than or equal to	0	0	0	0	1	1	1
De-escalate if number of DLTs equal to or greater than	1*	1*	2	2	2	2	3
Eliminate if number of DLTs equal to or greater than	2	3	3	3	4	4	4

Note. # of DLT is the number of subjects with at least 1 DLT in the first 28 days of dosing. Observations from IPDE will not be considered (Section 6.9). When none of the actions (i.e., escalate, de-escalate or eliminate) are triggered, stay at the current dose for treating the next cohort of subjects. *The SRC can decide to enroll an additional 3 subjects at this dose level. In case the multiple dosing regimens are initiated simultaneously and parallel enrollment is allowed in the multiple dosing regimens, the evaluation for BOIN rule for each dose level and regimen would be made separately.

In Table 5, if 1 out of 3 or 4 subjects experience a DLT during the DLT evaluation period, the algorithm recommends dose de-escalation, rather than staying at the current dose. Considering only information from 3 to 4 subjects will be initially available, the incidence of 1 DLT does not sufficiently establish that the MTD is exceeded. Under this situation, a recently published i3+3 design recommends staying in the current dose level if the observed number of DLTs is only 1 greater than the number required for dose escalation[32]. Therefore, the Safety Review Committee could decide to enroll an additional 3 subjects at that dose level, if only 1 of 3 or 4 subjects experiences a DLT.

Figure 3: Flowchart for Trial Conduct Using the BOIN Design

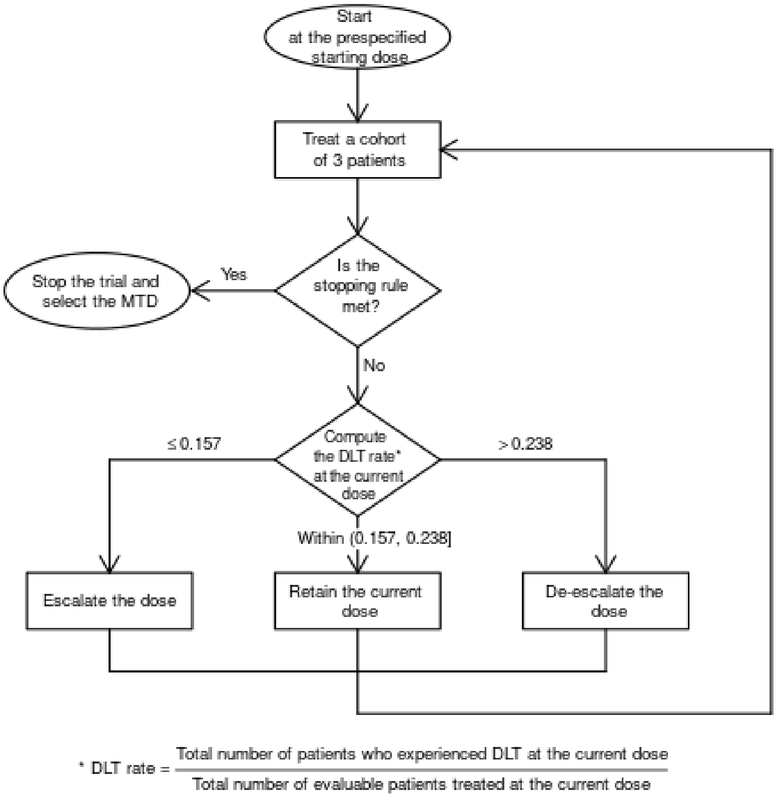

After the trial is completed, select the MTD based on isotonic regression as specified in Liu and Yuan (2015). This computation is implemented by the 'Shiny App' "BOIN" available at http://www.trialdesign.org. Specifically, select as the MTD the dose for which the isotonic estimate of the toxicity rate is closest to the target toxicity rate. If there are ties, select the higher dose level when the isotonic estimate is lower than the target toxicity rate and select the lower dose level when the isotonic estimate is greater than or equal to the target toxicity rate.

Up to approximately 18 additional subjects (no more than 9 additional subjects per dose) will be enrolled at a safe dose(s) below or at the MTD for additional experience with safety and efficacy. We will use the elimination boundaries in Table 5 for the toxicity monitoring.

The RP2D will be recommended by the SRC based on efficacy, safety and PK/PD data evaluation and may or may not be the same dose as the determined MTD dose.

3.2.1. Operation Characteristics for BOIN Design

Table 6 shows the operating characteristics of the trial design based on 1000 simulations of the trial using shiny app "BOIN" available at http://www.trialdesign.org. The operating characteristics show that the design selects the true MTD, if any, with high probability and allocates more subjects to the dose levels with the DLT rate closest to the target of 0.2.

Table 6: Operating Characteristics of the BOIN Design

Dose	1	2	3	4	5	6	Number of Subjects	% Early Stopping
**Scenario 1**								
True DLT Rate	0.2	0.34	0.4	0.45	0.5	0.55		
Selection %	57.5	20.9	4.9	2.4	1	0.1		13.2
**% Pts Treated**	41.4	29.8	14.4	8	4.4	2	18.2	
**Scenario 2**								
True DLT Rate	0.09	0.2	0.35	0.41	0.48	0.54		
Selection %	24.1	47.4	19.2	5.5	1	0.5		2.3
**% Pts Treated**	24.5	33.5	22.4	11.6	5.8	2.3	22	
**Scenario 3**								
True DLT Rate	0.02	0.08	0.2	0.37	0.43	0.49		
Selection %	1.5	22	56.4	16.4	2.6	1.1		0
**% Pts Treated**	8.4	22.4	33.8	21.4	9.6	4.4	23.7	
**Scenario 4**								
True DLT Rate	0.01	0.05	0.09	0.2	0.38	0.51		
Selection %	1	4.2	22.8	54.9	14.7	2.3		0.1
% Pts Treated	6.2	10.2	22.1	32.4	21.1	7.9	23.6	
**Scenario 5**								
**True DLT Rate**	0.01	0.04	0.07	0.1	0.2	0.37		
Selection %	0.2	1.4	6.6	23.1	49.5	19.2		0
**% Pts Treated**	5.4	7.9	12.3	22.8	31.9	19.8	22.9	
**Scenario 6**								
True DLT Rate	0.02	0.04	0.06	0.08	0.1	0.2		
Selection %	0.1	2	4	6.1	23.3	64.4		0.1
% Pts Treated	6.6	8.6	11	13.9	23.9	35.9	19.7	

Note: “% Early Stopping”" refers to early stopping due to excessive DLT.

The SRC will meet when there are a sufficient number of DLT-evaluable subjects to review in the current cohort who have completed the DLT evaluation period, and before proceeding to the next cohort at a higher dose level.

The dose-escalation recommendation obtained via the BOIN design will be reviewed by the SRC, along with the DLT data, other AE data, and available PK/PD and laboratory data before implementation of the next dose cohort.

If a lower dose is recommended by the BOIN method and confirmed by the SRC, then enrollment into the next lower dose level may be initiated, and additional cohorts may be opened to enroll subjects into a previously tested safe dose level to facilitate the evaluation of the dose-toxicity relationship.

3.2.2. Dose-Limiting Toxicity Definition

Dose-Limiting Toxicity (DLT) is defined in this study as a non-hematologic adverse event or abnormal laboratory value assessed as unrelated to disease progression, intercurrent illness, or concomitant medications and occurring during the DLT evaluation period that meets any of the following criteria, using the Common Terminology Criteria for Adverse Events (CTCAE) grading system, version 5.0. The DLT evaluation period will include the lead-in dosing period plus the first 28 days of dosing at the target dose. For example, if a subject receives 20 mg QD for 7 days followed by 50 mg QD for 7 days, before receiving the target dose of 100 mg QD, the DLT period will include total of (7+7+28) or 42 days.

- Hematologic DLT is defined as CTCAE Grade 4 neutropenia (absolute neutrophil count <0.5 x10^9/L) or thrombocytopenia (platelet count <25.0 x10^9/L) lasting for 42 days or more from Cycle 1 Day 1 (i.e., 42 days after the start of dosing at the target dose) in the absence of residual leukemia (i.e., with less than 5% blasts by morphology or residual leukemia by flow-cytometry).
  - Anemia will not be considered for the definition of DLT.
- CTCAE Grade 3 AST (SGOT) or ALT (SGPT) increased for at least 7 days or Grade 3 hyperbilirubinemia for at least 7 days.
- CTCAE Grade 3 AST (SGOT) or ALT (SGPT) increased accompanied by Grade 2 bilirubin increase
- CTCAE Grade 4 AST (SGOT) or ALT (SGPT) increased of any duration
- Any other non-hematological adverse events that are CTCAE Grade 3 or 4. However, the following adverse events are NOT considered DLTs:
  - Grade 3 nausea, vomiting and diarrhea will be considered DLT only if not controlled to Grade 2 or lower with optimal therapy within 72 hours (not including tube feeding, total parenteral nutrition, and/or hospitalization). Any Grade 3 gastrointestinal toxicity requiring tube feeding, total parenteral nutrition, and/or hospitalization and any Grade 4 gastrointestinal toxicity will be considered DLT.
  - Grade 3 or 4 biochemical abnormalities (e.g., lipase or amylase increased) will only be considered DLT if accompanied by clinical consequences.
  - Grade 3 or 4 isolated electrolyte abnormalities must be without clinical consequence and resolve, with or without intervention, to less than Grade 2 levels within 72 hours.
  - Grade 3 infusion reaction if successfully managed and which resolves within 72 hours
  - Grade 3 or 4 Tumor Lysis Syndrome if it is successfully managed clinically and resolves within 7 days without end-organ damage

  - Grade 3 or 4 Differentiation Syndrome which improves to ≤ Grade 1 within 7 days of the start date of ≥ Grade 3 Differentiation Syndrome and which is not associated with end-organ damage. Note: Grade 1 is mild, Grade 2 is moderate, Grade 3 is serious, and Grade 4 is life-threatening.

- CTCAE Grade 3 QTcF prolongation (based on the average of 3 triplicate 12-lead readings or Holter monitor reading)
- Results in dose-reduction or discontinuation of therapy
- Any treatment-related death;
- Subjects not considered evaluable due to insufficient dosing (at least 75% of doses of study drugs) and who do not develop a toxicity that is considered a DLT will be replaced. These subjects may continue therapy on trial after discussion with the Principal Investigator if they are having clinical benefit and the reasons for continuation and potential benefit/risk profile for the subject must be clearly documented in the medical records.
- **Phase 2 only**: For subjects in venetoclax- or Mini-HCVD-containing regimens (cohorts C and D) who do not receive venetoclax or Mini-HCVD on Day 1 due to logistical and/or financial reasons, the DLT period will be extended to 28 days from the first dose of venetoclax or Mini-HCVD. For evaluation of hematologic-related DLT, the DLT evaluation period will be 42 days from the first dose of venetoclax or Mini-HCVD.
- Subjects must receive at least 75% of the doses of DS-1594b, azacitidine (Phase 2 only), venetoclax (Phase 2 only), or miniHCVD (Phase 2) during the first 28 days on trial (i.e., during Cycle 1) to be considered evaluable during the DLT evaluation period. Subjects who receive less than these planned number of doses of each drug during the first 28 days on trial will not be considered evaluable during the DLT evaluation period. However, if such subjects develop a toxicity that would normally be considered a DLT, this will still be captured as a DLT. Any toxicity that meets the criteria for DLT during the first 28 days of trial in a subject who has received at least one dose of trial medication will constitute a DLT.
- For subjects who discontinue therapy, the reason for treatment discontinuation will be documented.
- Dose modifications other than the ones mentioned above can be considered after discussion with the PI and proper documentation of the rationale.

3.2.3. DDI Sub-study with Anti-fungal Agents that are Strong CYP3A Inhibitors

3.2.3.1. Sub-Study Rationale

Since AML subjects are at a high risk for fungal infections, to reduce the likelihood and severity of fungal infections, azole antifungal agents such as fluconazole, posaconazole and voriconazole are commonly administered. However, these azole compounds carry DDI risks because of their inhibitory effect on CYP3A enzymes. Based on nonclinical assessment, DS-1594b is a CYP3A

substrate (see Section 2.4.2), thus DDI assessment for DS-1594b and antifungal agents is necessary to identify the DS-1594b dosing regimen that can be concomitantly given with antifungals and potentially extend to other strong CYP3A inhibitors. This DDI assessment is also aligned with the recommendation from the FDA Industry Guidance for developing drugs and biological products for AML treatment (Aug 2020). The guidance recommends studying DDI with moderate and strong CYP3A inhibitors early in clinical development if the investigational AML drug is a CYP3A substrate, to determine dose modification, if needed, when concomitantly administered with moderate and/or strong CYP3A inhibitors.

3.2.3.2. Sub-study Design

Following dosing in Phase 1 Dose Escalation and MTD declaration, approximately 12 additional subjects will be enrolled into a drug-drug interaction (DDI) sub-study within the Phase 1 portion of the study at a predetermined dose level (at least 50% below the RP2D, as recommended by the SRC) to assess the effect of multiple doses of antifungal agents (i.e., posaconazole [N ~ 6], voriconazole [N ~ 6]) that are strong CYP3A inhibitors, on the steady-state PK of DS-1594b. Based on preliminary assessment using physiologic-based modeling, the magnitude of PK increase of DS-1594b ranges from 30% (with posaconazole) to 2-fold (with voriconazole).

This estimate will be further assessed based on emerging clinical and nonclinical data and the information will be provided to the SRC for consideration in dose selection. Nonetheless, the dose will be at least 50% below the declared RP2D. These subjects can be the same subjects included in the Phase 1 expansion for RP2D determination.

This DDI sub-study is a single sequence, crossover design. Enrollment will complete on the posaconazole sub-study before enrollment will begin on the voriconazole sub-study. Subjects will receive twice-daily or daily oral dosing of DS-1594b alone at the assigned dose from Cycle 1 Day 1 to Cycle 1 Day 8. On Cycle 1 Day 9 to Cycle 1 Day 18, subjects will receive the same assigned DS-1594b dose with either (a) 300 mg oral posaconazole twice on Cycle 1 Day 9 and once daily on Cycle 1 Day 10 to Cycle 1 Day 18 or (b) 400 mg oral voriconazole twice on Cycle 1 Day 9 and 200 mg twice daily from Cycle 1 Day 10 to Cycle 1 Day 18.

The anti-fungal agents are planned to be given for 10 consecutive days as stated above to ensure steady-state is reached in order to maximize inhibitory effect on CYP3A-mediated metabolism of DS-1594b. After completion of the sub-study (Cycle 1 Day 18 is the last day of the sub-study), subjects will continue with DS-1594b dosing alone starting on Cycle 1 Day 19. PK sampling timepoints of DS-1594b are outlined in Table 19. Due to the one-way crossover nature of the sub-study, the lead-in period will not be applied.

Note: *If the use of posaconazole is essential for subject care during the dose escalation phase (i.e., prior to MTD/RP2D declaration), the subject can enter the DDI sub-study sooner, starting from the third dose escalation cohort. Voriconazole is not allowed as DDI perpetrator prior to MTD declaration given it is a more potent CYP3A inhibitor compared to posaconazole. To avoid interference with the DLT evaluation period (first 28-day cycle), subjects will receive oral twice daily or daily dosing of DS-1594b alone at the assigned dose for the entire Cycle 1. Starting on Cycle 2 Day 1 to Cycle 2 Day 10, subjects will receive either the same assigned DS-1594b dose provided that dose level has been declared safe from the DLT evaluation, or at one dose level lower with 300 mg oral posaconazole twice on Cycle 2 Day 1 and once daily on Cycle 2 Day 2 to*

Cycle 2 Day 10. After completion of the sub-study (Cycle 2 Day 10 is the last day of the sub-study), subjects will continue with DS-1594b dosing alone starting on Cycle 2 Day 11. PK sampling timepoints of DS-1594b for these subjects are outlined in Table 20. These subjects will contribute to the overall sample size of ~ 12 for DDI sub-study.

The entry criteria for subjects in DDI sub-study will be the same as all other subjects, except for additional concomitant medications restrictions for CYP3A modulators and gastric reducing agents (see Section 4.2).

3.2.3.3. Sub-Study PK Evaluability Criteria

Subjects should meet the following minimum criteria to be considered evaluable for the DDI assessment. PK non-evaluable subjects will be replaced.

- On Cycle 1 Day 8 and Cycle 1 Day 18 (or Cycle 2 Day 10 for subjects entering DDI sub-study before MTD declaration), subject takes DS-1594b according to the assigned dose.
- Subject has an interpretable PK profile following administration of the assigned dose of DS-1594b on Cycle 1 Day 8 and Cycle 1 Day 18 (or Cycle 2 Day 10).
- Subject does not vomit within 4 hours after receiving either dose of DS-1594b or antifungal agents (posaconazole or voriconazole) on PK sampling days.
- Subject receives at least 5 consecutive twice daily or daily doses of DS-1594b at the assigned dose prior to the co-administration with antifungal agents
- Subject receives at least 5 consecutive twice daily or daily doses of DS-1594b at the assigned dose when co-administered with antifungal agents
- Subject receives the assigned dose and appropriate prandial condition of the antifungal agents.

3.2.4. Food Effect Sub-study

3.2.4.1. Sub-Study Rationale

As DS-1594b is administered orally, the potential impact of food intake on oral bioavailability is an important consideration. In addition, potential combination agents, such as venetoclax, require administration under fed conditions. Determining the food effect early for DS-1594b will help optimize the prandial condition for DS-1594b dosing. Based on in vitro and animal studies, minimal food effect has been observed thus far.

3.2.4.2. Sub-Study Design

Following MTD declaration, approximately 6 additional subjects will be enrolled into a food effect sub-study to evaluate the effect of a standard meal on the steady-state PK of DS-1594b.

This sub-study follows a single sequence, crossover design. Subjects will receive oral twice-daily or daily dosing of DS-1594b at a predetermined dose level (at least 50% lower than the RP2D) within 0.5 hours after a standard meal (breakfast and dinner), defined as one containing 600 kcal

and 30% fat (~21g fat), from Cycle 1 Day 1 to Cycle 1 Day 8. Subjects should refrain from food and drink, except water, 2 hours before dosing (except for breakfast and dinner) and 2 hours after dosing.

From Cycle 1 Day 9 to Cycle 1 Day 18, subjects will receive the same DS-1594b dose under fasting conditions (i.e., subjects refrain from food and drink, except water, for 2 hours before dosing and for 2 hours after dosing). See Section 5.1.2 (under "food effect sub-study only") for more details regarding dose administration guidance. On days when PK blood samples will be taken, DS-1594b dosing and meal regimens will be administered at the investigational site. After completion of the sub-study (Cycle 1 Day 18 is the last day of sub-study), subjects will continue with DS-1594b dosing under fasting conditions, same as patients not in sub-study (DS-1594b dosing at least 1 hour before or 2 hours after a meal) starting on Cycle 1 Day 19. Due to the one-way crossover nature of the sub-study, the lead-in period will not be applied.

Note: The entry criteria will be the same as the preceding cohorts, except for additional concomitant medications restrictions for CYP3A modulators and gastric reducing agents (see Section 4.2). PK sampling timepoints of DS-1594b are outlined in Table 21.

3.2.4.3. Sub-Study PK Evaluability Criteria

Subjects should meet the following minimum criteria to be considered evaluable for the food effect assessment. PK non-evaluable subjects will be replaced.

- On Cycle 1 Day 8 and Cycle 1 Day 18, subject takes DS-1594b according to the assigned dose.
- Subject has an interpretable PK profile following administration of the assigned dose of DS-1594b on Cycle 1 Day 8 and Cycle 1 Day 18.
- Subject takes DS-1594b under the assigned dose and prandial conditions (first 7 days under fed state, then under fasted state afterwards) for at least 5 consecutive days prior to full PK sampling days on Cycle 1 Day 8 and Cycle 1 Day 18.
- Subject does not vomit within 4 hours after DS-1594b dosing on full PK sampling days.

For toxicity monitoring in each group of the DDI sub-study and food effect sub-study we will use the elimination boundaries in Table 5 for BOIN design.

3.3. Phase 2 Design

Enrollment in Phase 2 will start once the RP2D has been determined in Phase 1. This Phase 2 study will have four cohorts:

- Cohort A: DS-1594b monotherapy for R/R AML with MLL-r,
- Cohort B: DS-1594b monotherapy for R/R AML with NPM1m,
- Cohort C: DS-1594b in combination with azacitidine and venetoclax for R/R AML with NPM1m or MLLr
- Cohort D: DS-1594b in combination of mini-HCVD for R/R ALL with MLLr

Subjects enrolled in Cohorts A and B will receive the RP2D dose of DS-1594b determined in Phase 1. Cohorts C and D will enroll an initial 6 subjects in a safety run-in at one dose level below the RP2D (Dose level 0) with DS-1594b dose titration as specified below. Lead-in dosing may be implemented if recommended by the SRC.

Cohorts A and B (single agent cohorts) will enroll a maximum of 26 subjects, and Cohorts C and D (combination cohorts) will enroll a maximum of 32 subjects, to allow reasonable determination of CRc within 3 months of therapy initiation.

For the combination cohorts (C & D) there will be a safety run-in evaluation with pre-defined toxicity stopping rules to be implemented for early DLT evaluation as per the following plan:

- Safety run-in evaluation by the Safety Review Committee of the first 6 subjects in cohorts C and D will occur after 6 subjects have completed at least one cycle of treatment (or at least 28 days from the first day of treatment).
- Enrollment will be paused while the first 6 subjects are evaluated for safety. Each combination cohort will be evaluated independently.
  - If ≤ 1 of the 6 subjects experiences DLT in the first cycle of treatment, the treatment combination will be considered as safe and the DS-1594b dose can be escalated to the RP2D (Dose level 1) for the treatment of the next 6 patients (repeat the safety run-in evaluation). If the RP2D (Dose level 1) is considered safe, continue enrollment of an additional 20 subjects at the RP2D (i.e., total subjects treated at the RP2D will be 26 subjects; total cohort size will be 32 subjects). If ≥2 of 6 subjects at the RP2D experiences DLT in the first cycle of treatment, de-escalate and continue enrollment of an additional 20 subjects at Dose Level 0.
  - If ≥2 of 6 subjects have DLTs in the first cycle of treatment at Dose Level 0, then an additional 6 subjects will enroll at a next lower dose level to be determined by the Principal Investigator and Safety Review Committee after reviewing the safety, efficacy, and PD data from the first 6 subjects enrolled (Table 7, Table 8). If ≤ 1 of the additional 6 subjects treated at the lower dose level experience DLTs, this treatment combination will be considered as safe and continue to enroll to a total of 26 subjects (i.e., an additional 20 subjects at the lower dose level; total cohort enrollment of 32 subjects). If ≥2 of these 6 additional subjects experienced DLTs, then enrollment at the current dose level will be closed.
- Subjects must receive at least 75% of the doses of DS-1594b, azacitidine (Phase 2 only), venetoclax (Phase 2 only), or mini-HCVD (Phase 2) during the first 28 days on trial (i.e. during Cycle 1) to be considered evaluable during the DLT evaluation period. Any subjects who are not evaluable for DLT in either cohort during the safety-run in period will be replaced for the assessment of DLT rate. This will allow for early identification of significant toxicities and early stoppage in the combination cohorts.
- The Safety Review Committee (SRC) will meet when there are enough DLT-evaluable subjects to review in each cohort who have completed the DLT

evaluation period. The SRC will review the DLT data, other AE data, and available PK/PD, ECG, and laboratory data before proceeding to expansion of each of the cohorts to enroll the additional 20 subjects.

3.3.1. Rationale for Combination Cohorts (C&D)

AML and ALL are highly heterogenous diseases and patients often harbor more than one driver mutation[2]. While recent studies of novel molecularly targeted agents, such as FLT3 inhibitors[28] and IDH1/2 inhibitors[29,30], have demonstrated improved response rates and overall survival as monotherapy in patients with relapsed or refractory AML, there is still a high unmet medical need as patients will ultimately succumb to the disease. Combination therapies with molecularly targeted agents have emerged as an attractive strategy to improve efficacy while maintaining a tolerable safety[31].

The primary objective of the Phase 2 combination Cohorts C & D is to evaluate the safety and tolerability, as well as efficacy, of DS-1594b administered with standard of care backbone regimens. The RP2D will be selected based upon the cumulative safety, efficacy and PK data at the end of the Phase 1 portion, as the anticipated optimal dose considering benefit-risk. Since this will be the first evaluation of DS-1594b administered in combination, both Cohorts C & D will include a safety run-in period (beginning enrollment at one dose level below the RP2D) with pre-specified rules for dose titration under the supervision of the Principal Investigator and the Safety Review Committee, as described in Section 3.3. Additionally, all cohorts will be monitored for toxicity, with pre-specified criteria for early termination if excessive toxicity is predicted, as described in Section 9.3.

4. SUBJECT SELECTION

4.1. Inclusion Criteria

Each subject must meet **all** the following inclusion criteria to be enrolled in this study.

a. Provision of written (signed) informed consent form (ICF) by the subject or legal guardian prior to the performance of any study-specific procedures, according to International Council on Harmonisation (ICH) and local regulatory requirements. Subject must be fully informed about their illness and the investigational nature of the study protocol (including foreseeable risks and possible toxicities) and must sign and date an Institutional Review Board (IRB)/Independent Ethics Committee (IEC) approved informed consent form (ICF)(including Health Insurance Portability and Accountability Act authorization [HIPAA], if applicable) before performance of any study-specific procedures or examinations.

b. Subjects must be willing and able to comply with the protocol.

c. Subjects with AML or ALL, diagnosed according to the 2016 criteria by the World Health Organization (WHO)[26,25], who are refractory or relapsed (any salvage) with no available therapies or not candidates for available therapies. For subjects with prior MDS or chronic myelomonocytic leukemia (CMML) or MPN who transformed to

AML, therapy received for MDS, CMML, or MPN is NOT considered as prior therapy for AML except for MDS or CMML treated with HMAs. Subjects with MDS or CMML treated with HMA therapies who progress to AML and have no available therapies or are not candidates for available therapies, will be eligible at the time of progression to AML. In Phase 1: all R/R AML or R/R ALL subjects irrespective of mutations will be eligible. In Phase 2 Cohort A only R/R AML with MLLr will be eligible. In Phase 2 Cohort B only R/R AML with NPM1m will be eligible. In Phase 2 Cohorts C and D: Only R/R AML or R/R ALL subjects with an MLLr or NPM1m will be eligible.

d. Prior therapy with hydroxyurea, chemotherapy, biological or targeted therapy (e.g., FLT3 inhibitors, other kinase inhibitors), or hematopoietic growth factors is permitted.

e. Age 18 years or older.

f. Eastern Cooperative Oncology Group (ECOG) Performance Status 0, 1, or 2.

g. Adequate hepatic organ function: total bilirubin ≤ 1.5 times upper limit of normal (x ULN); aspartate aminotransferase or alanine aminotransferase ≤ 2.5 x ULN (aspartate aminotransferase or alanine aminotransferase ≤ 5.0 x ULN if deemed related to leukemia by the treating physician)

h. Creatinine clearance ≥ 50 mL/min as calculated using the modified Cockcroft-Gault equation.

i. Serum electrolytes within the institution's normal limits: potassium, calcium (total calcium, calcium corrected for serum albumin in case of hypoalbuminemia or ionized calcium) and magnesium. If outside of the institution's normal range, subject will be eligible when electrolytes are corrected.

j. In the absence of rapidly progressive disease, the interval from prior treatment to the time of initiation of protocol therapy will be at least 14 days for prior anti-leukemic therapy with the exception of hydroxyurea as noted below OR at least 5 half-lives for cytotoxic/noncytotoxic agents, whichever is shorter. The half-life for the therapy in question will be based on published pharmacokinetic literature (abstracts, manuscripts, investigator brochure's, or drug-administration manuals) and will be documented in the protocol eligibility document. Since the effect of therapy may be delayed, use of hydroxyurea for subjects with rapidly proliferative disease is allowed before the start of study therapy and on study and hydroxyurea will not require a washout.

k. Concurrent therapy for CNS prophylaxis or continuation of therapy for controlled CNS disease is permitted. Subjects with a known history of CNS disease or leukemic brain metastasis must have been treated locally, have at least 3 consecutive LPs with no evidence of CNS leukemia, and must be clinically stable for at least 4 weeks prior to enrollment and have no ongoing neurological symptoms that in the opinion of the treating physician are related to the CNS disease (sequelae that are a consequence of the treatment of the CNS disease are acceptable).

l. Females must be surgically or biologically sterile or postmenopausal (amenorrheic for at least 12 months) or if of childbearing potential, must have a negative serum or urine pregnancy test within 72 hours before the start of the treatment.

m. Women of childbearing potential must agree to use an adequate method of contraception during the study and until 4 months after the last treatment. Males must be surgically or biologically sterile or agree to use an adequate method of contraception during the study until 3 months after the last treatment. In addition, sexually active male subjects must agree to use condoms during the study until 3 months after the last treatment to prevent the potential transmission of the study drug and/or metabolites via semen. Adequate methods of contraception include:

   - Total abstinence when this is in line with the preferred and usual lifestyle of the subject. Periodic abstinence (e.g., calendar, ovulation, symptothermal, post-ovulation methods) and withdrawal are not acceptable methods of contraception.
   - Female sterilization (have had surgical bilateral oophorectomy with or without hysterectomy) or tubal ligation at least six weeks before taking study treatment. In case of oophorectomy alone, only when the reproductive status of the woman has been confirmed by follow up hormone level assessment
   - Male sterilization (at least 6 months prior to screening). For female subjects on the study, the vasectomized male partner should be the sole partner for that subject
   - Combination of any of the two following (a+b or a+c or b+c)
      a. Use of oral, injected or implanted hormonal methods of contraception or other forms of hormonal contraception that have comparable efficacy (failure rate <1%), for example hormone vaginal ring or transdermal hormone contraception. Contraceptives containing ethinyl estradiol should be used with caution (Section 5.6.3).
      b. Placement of an intrauterine device (IUD) or intrauterine system (IUS).
      c. Barrier methods of contraception: Condom or Occlusive cap (diaphragm or cervical/vault caps) with spermicidal foam/gel/film/cream/ vaginal suppository.
   - In case of use of oral contraception, women should have been stable on the same pill before taking study treatment.
   - Note: *Oral contraceptives are allowed but should be used in conjunction with a barrier method of contraception due to unknown effect of drug-drug interaction. Women are considered post-menopausal and not of child-bearing potential if they have had 12 months of natural (spontaneous) amenorrhea with an appropriate clinical profile (e.g., age appropriate, history of vasomotor symptoms) or have had surgical bilateral oophorectomy (with or without hysterectomy) or tubal ligation at least six weeks ago. In the case of oophorectomy alone, only when the reproductive status of the woman has been confirmed by follow up hormone level assessment is she considered not of child-bearing potential.*

4.2. Exclusion Criteria

a. Subjects with a known allergy, hypersensitivity, or contraindication to the protocol therapies or any of their components to be used in the arm the subject is to be enrolled on.
b. Uncontrolled or significant cardiovascular disease, including any of the following:
   - Bradycardia of less than 50 beats per minute, unless the subject has a pacemaker;
   - QTcF interval >450 msec;
   - Diagnosis of or suspicion of long QT syndrome (including family history of long QT syndrome);
   - Systolic blood pressure ≥180 mmHg or diastolic blood pressure ≥110 mmHg;
   - History of clinically relevant ventricular arrhythmias within 6 months prior to screening (e.g., ventricular tachycardia, ventricular fibrillation, or Torsade de Pointes);
   - History of second (Mobitz II) or third-degree heart block (subjects with pacemakers are eligible if they have no history of fainting or clinically relevant arrhythmias while using the pacemaker);
   - History of uncontrolled angina pectoris, unstable angina or myocardial infarction, CABG, CVA, TIA, symptomatic pulmonary emboli within 6 months prior to Screening;
   - New York Heart Association Class 3 or 4 heart failure;
   - Left ventricular ejection fraction (LVEF) ≤50or less than the institutional lower limit of normal;
   - Complete left bundle branch block (right bundle branch block is permitted, but requires manual reading of the QTc interval);
   - Active cardiac dysrhythmias of NCI CTCAE grade ≥ 2 (e.g., atrial fibrillation).
c. Persisting toxicity related to prior therapy of Grade >1 NCI-CTCAE v 5.0; however, alopecia and sensory neuropathy Grade 2 or lower is acceptable
d. Underwent HSCT within 90 days of the first dose of protocol therapy, or subjects with clinically significant (Grade 2 or greater) graft-versus-host disease (GVHD) (the use of topical steroids for ongoing cutaneous GVHD is permitted)
e. Subjects with symptomatic CNS leukemia or subjects with poorly controlled CNS leukemia.
f. Active and uncontrolled disease (active infection requiring systemic therapy, fever likely secondary to infection within prior 48 hours, uncontrolled hypertension despite adequate medical therapy as judged by the treating physician).
g. Active (uncontrolled, metastatic) other malignancies
h. Major surgery within 28 days prior to the first dose of protocol therapy

i. Known dysphagia, short-gut syndrome, gastroparesis, or other conditions that limit the ingestion or gastrointestinal absorption of drugs administered orally

j. Known history of testing positive for HIV or known acquired immunodeficiency syndrome (test at screening only if required by local regulations)

k. Known history of Hepatitis B virus (HBV) or hepatitis C virus (HCV) infection with active Hepatitis B or C infection at screening (positive HBV surface antigen or HCV RNA if anti-HCV antibody screening test positive)

l. Vaccination with live-attenuated vaccines within 4 weeks of the first dose of study drug and while on trials is prohibited (COVID-19 vaccination is permitted)

m. Subjects who are currently receiving treatment with medication that meet one of the following criteria and that cannot be discontinued at least one week prior to the start of DS-1594b treatment:

   – Medications that may prolong QTc interval and have a known risk of inducing Torsades de Pointes unless it is vital for the care of the subjects (see Appendix Table 31)

   – Strong inhibitors or inducers of CYP3A (see Appendix Table 32)

   – CYP3A and CYP2C19 substrates with narrow therapeutic index (see Appendix Table 32)

n. Subjects who consume grapefruit products, Seville oranges, or Star fruit within 3 days prior to the first DS-1594b administration and until the last day of DS-1594b is completed.

o. **Additional exclusion criteria for the DDI sub-study and food effect sub-study (see Appendix** Table 34)

   – Subjects who are currently receiving moderate inhibitors or inducers of CYP3A who cannot discontinue at least one week prior to the start of DS-1594b treatment till the end of sub-study period (note that strong CYP3A inhibitors or inducers still need to be restricted as defined in Section 4.2).

   – Subjects who are currently receiving proton pump inhibitors who cannot discontinue at least 2 days prior to the start of DS-1594b treatment till the end of sub-study period.

p. Other severe acute or chronic medical conditions that is active and not well controlled including renal, skeletal muscle, adrenal insufficiency, colitis, inflammatory bowel disease, or psychiatric conditions including recent (within the past year) or active suicidal ideation or behavior; or laboratory abnormalities that may increase the risk associated with study participation or study treatment administration or may interfere with the interpretation of study results and, in the judgment of the investigator, would make the subject inappropriate for entry into this study.

q. Subjects unwilling or unable to comply with the protocol, including:

   – Pregnant or breastfeeding women or women of childbearing potential who are unable to comply with appropriate contraception as outlined in Section 4.1m or

who plan to become pregnant while in the study or for at least 6-7 months after last administration of study treatment

– Known alcohol or drug abuse within the last 1 year

– In a man whose sexual partner is a woman of childbearing potential, unwillingness or inability to use an acceptable contraceptive method for the entire study period and for at least 3 months after study completion

r. Acute promyelocytic leukemia (APL).

s. Uncontrolled or poorly controlled adrenal or pituitary disease (including adrenal insufficiency, Addison's disease, Cushing's disease)

5. TREATMENT PLAN

5.1. Phase 1 Dose Administration

5.1.1. Study Treatment

The orally administered film-coated tablet formulation of DS-1594b will be supplied by Daiichi Sankyo and are in 20 mg, 50 mg, and 200 mg dose strengths.

The first dose cohort will start at 70 mg twice daily, approximately every 12 hours. For provisional dose levels beyond the first dose cohort, see Figure 1. Alternative doses or dosing regimens may be enrolled based on emerging clinical data, including a lead-in dose regimen, intermediate dose levels or dose de-escalation. BID and QD cohorts can be enrolled in parallel and will be evaluated independently for DLT.

5.1.2. Dose Administration Guidance

DS-1594b will be administered orally on a continuous twice daily (or once daily) regimen. In this study a complete cycle of treatment is defined as 28 days at the target dose. Lead-in dosing may be administered to mitigate the possible clinical risks associated with rapidly differentiating leukemic blasts (see Section 3.1.1 for details). The investigator must instruct the subject to take the study drug (DS-1594b) exactly as prescribed:

1. For BID dosing, subjects should take DS-1594b tablets twice daily at approximately the same time each day for morning and evening doses; for QD dosing, subjects should take DS-1594b tablets once daily at approximately the same time each day for morning doses.
2. Each dose of DS-1594b is to be taken with a glass of water (at least 8 ounces – approximately 250 mL) and consumed over as short a time as possible (i.e., not slower than 1 tablet every 2 minutes).
3. Subjects should be instructed to swallow the tablets whole and not to chew them.
4. DS-1594b should be administered under fasting condition, at least 1 hour before or 2 hours after a meal.

5. For BID dosing, the morning and evening doses should be taken 12 (± 4) hours apart, although 12-hour interval is recommended. If a dose is not taken within 4 hours of the planned dosing time, the missed dose should not be replaced. On days when serial PK blood samples are to be collected especially those with 12 hours post-dose timepoint (see Section 8.4.1), subjects should keep the 12-hour interval between morning and evening doses.
6. On days when PK blood samples are to be collected, subjects will be instructed to hold their dose until arrival at the study center; the site will remind subjects one day before not to take the morning dose at home. DS-1594b will be administered at the site in the morning after the pre-dose ECG assessment and PK blood draw and prior to the post-dose PK blood draws. The exact date/time of drug administration should be recorded in the eCRF. If a subject vomits within the first 4 hours post-treatment on a full PK sampling day, the exact time of vomiting should be recorded on the eCRF.
7. All pre-dose PK samples should be drawn just before DS-1594b dosing. The sampling time of the pre-dose PK sample and the dosing time of DS-1594b must be precisely recorded in the eCRF. Furthermore, the dosing time of DS-1594b on the previous day must be precisely recorded in the eCRF.

   DDI sub-study only:

   – On days with posaconazole co-administration, DS-1594b will be dosed first under fasting condition. One hour later, posaconazole will be dosed (within 0.5 hours) following a breakfast.

   – On days with voriconazole co-administration, DS-1594b and voriconazole can be administered together since both drugs will be given under fasting conditions.

   Food effect sub-study only:

   – Subjects in this sub-study do not follow bullet (4) above but the bullets below for prandial condition.

   – For the morning doses from Cycle 1 Day 1 to Cycle 1 Day 8, DS-1594b will be administered immediately (within 0.5 hours) following a standard breakfast defined as one containing 600 kcal and 30% fat (~ 21g fat). Subjects should refrain from food and drink, except water, for at least 2 hours after dosing; for the evening doses (12 hours apart from morning dose) (applicable for BID dosing only), DS-1594b will also be administered immediately (within 0.5 hours) following a standard dinner (same meal content as breakfast). Subjects should refrain from food and drink, except water, for 2 hours before dosing (except for breakfast and dinner) and for 2 hours after dosing.

   – Starting from Cycle 1 Day 9 to Cycle 1 Day 18, DS-1594b should be administered under fasting condition (i.e., subjects should refrain from food and drink, except water, for 2 hours before dosing and for 2 hours after dosing). After completion of the sub-study (Cycle 1 Day 18 is the last day of sub-study), subjects will continue with DS-1594b dosing under fasting conditions, same as

patients not in the food effect sub-study (DS-1594b dosing at least 1 hour before or 2 hours after a meal) starting on Cycle 1 Day 19.

5.2. Phase 2 Cohort A: R/R AML with MLL-rearrangement

5.2.1. Dose Administration Guidance

The general dose administration guidance for DS-1594b as listed in Section 5.1 should be followed.

5.3. Phase 2 Cohort B: R/R AML with NPM1 mutation

5.3.1. Dose Administration Guidance

The general dose administration guidance for DS-1594b as listed in Section 5.1 should be followed.

5.4. Phase 2 Cohort C: R/R AML Treated with the Combination of Azacitidine and Venetoclax with DS-1594b

5.4.1. Study Treatment of Study Drug and Other Combination Drugs

Subjects will be treated according to the following dose levels. Lead-in dosing for DS-1594b may be implemented by the SRC based on emerging clinical data.

Table 7: Dose Levels for Azacitidine, Venetoclax, and DS-1594b

Dose Level	Azacitidine (mg/m^2)	Venetoclax (400 mg or equivalent)	DS-1594b
-1	75 mg/m^2 D1-7	D1-28[a, b, c, d]	Lower dose of DS-1594b as recommended by SRC
0 (Starting dose)	75 mg/m^2 D1-7	D1-28 [a, b, c, d]	One dose level below the RP2D of DS-1594b from Phase 1
1	Not applicable	Not applicable	RP2D of DS-1594b from Phase 1

Venetoclax:

[a] During Cycle 1, venetoclax administered orally will be dose escalated daily to the goal dose of 400mg daily. Subjects will receive 100mg on Day 1, 200mg on Day 2 and 400mg on Day 3 and onwards, or adjusted ramp up per drug label if on concomitant azoles. The goal is to administer 28 days of venetoclax in Cycle 1 unless marrow remission with concomitant marrow hypocellularity and/or myelosuppression is confirmed earlier than Cycle 1 Day 28. In this case the venetoclax may be stopped earlier to avoid venetoclax related myelosuppression or further marrow hypo/aplasia after discussion and approval from the PI. Venetoclax dose ramp-up as described in Section 5.4.2
[b] If a bone marrow remission (<5% blasts) or aplasia/hypoplasia (<10% cellularity or insufficient sample) is not confirmed on the Day 21 bone marrow, subjects should continue venetoclax until day 28 and have a repeat bone marrow on Day 28 (+/- 5 days). If the Day 28 bone marrow shows ≥ 5% blasts proceed with cycle 2 if this is in the best interest of the subject (those with C1D28 bone marrow ≥ 5% are allowed to receive 21 days of venetoclax in Cycle 2 after discussion and approval from the PI). If the Day 28 bone marrow shows < 5% blasts or aplasia/hypoplasia follow steps outlined in the Azacitidine administration information “a” and “b” below.
[c] Venetoclax will be administered orally daily on Days 1 to 28 of the first cycle; and may be reduced to Venetoclax Days 1 to 21 or less for subsequent cycles after PI approval.
[d] Results from published in vitro and clinical DDI studies revealed a need to modify the dose of venetoclax when it is co-administered with moderate or strong CYP3A inhibitors or P-gp inhibitors (VENCLEXTA® USPI). Based on in vitro assessment, DS-1594b has the potential to inhibit CYP3A-mediated metabolism and P-gp mediated transport of venetoclax in vivo. Thus, dose level(s) of venetoclax should be recommended by the SRC based on emerging DS-1594b PK and safety data, and the dose level of venetoclax and DS-1594b may be adjusted after review of the data collected during the safety run-in evaluation.

Azacitidine:

Azacitidine (75 $mg/m^2/day$) will be administered subcutaneously (SQ) or intravenously (IV) for the first 7 days of every cycle. Azacitidine may be given in a 5on - 2off - 2on or 4on - 2off – 3 on or other similar modified schedule if the subject is not able to get weekend infusions. Azacitidine is administered in the clinic or chemotherapy administration area. Both SQ and IV forms of administration are FDA approved and are allowed and considered interchangeable. Subjects may

start receiving azacitidine by one route and change to the other, and administration schedules from one schedule and change to the other at any time as needed based on subject and/or physician preference.

- In Cycle 1, subjects should undergo a bone marrow aspiration and biopsy on Day 21 (+/- 4 days). Subjects who achieve a confirmed marrow remission (i.e., bone marrow blasts < 5% by morphology) or marrow aplasia/hypoplasia (≤ 10% cellularity or insufficient sample) may discontinue venetoclax for the remainder of the cycle and be monitored for count recovery.
  - If the Day 21 bone marrow shows < 5% blasts, the venetoclax may be held, and consider delaying cycle 2 till ANC > 0.5 x 10^9/L and platelets are > 30 x 10^9/L without platelet transfusion support for > 5 days.
  - If the Day 21 bone marrow shows aplasia/hypoplasia (≤ 10% cellularity or insufficient sample), the venetoclax may be held on Day 21 to avoid venetoclax related myelosuppression or further marrow hypo/aplasia, and a repeat bone marrow should be performed on Day 28 (+/- 4 days) to confirm response and MRD assessment. If the Day 28 bone marrow shows ≥ 5% blasts, proceed with Cycle 2 if this is in the best interest of the subject (those with C1D28 bone marrow ≥ 5% are allowed to receive 21 days of venetoclax in Cycle 2). If the Day 28 bone marrow shows < 5% blasts, the venetoclax may be continued to be held, and consider delaying cycle 2 till ANC > 0.5 x 10^9/L and platelets are >30 x 10^9/L without platelet transfusion support for > 5 days. If the Day 28 bone marrow shows persistent aplasia/hypoplasia (≤ 10% cellularity or insufficient sample) venetoclax may be held to avoid venetoclax related myelosuppression or further marrow hypo/aplasia and consider repeating a bone marrow in approximately 10-14 days.
  - If a bone marrow remission (< 5% blasts) or aplasia/hypoplasia is not noted on the Day 21 bone marrow, subjects should continue venetoclax until day 28 and have a repeat bone marrow on Day 28 (+/- 5 days). If the Day 28 bone marrow shows ≥ 5% blasts proceed with Cycle 2 if this is in the best interest of the subject (those with C1D28 bone marrow ≥ 5% are allowed to receive 21 days of venetoclax in Cycle 2 after discussion with and approval from the PI). If the Day 28 bone marrow shows < 5% blasts or aplasia/hypoplasia follow steps outlined in "a" and "b" above.
  - Treatment interruptions and dosing schedules other than the ones mentioned above can be considered after discussion with the PI and proper documentation of the rationale.
  - DS-1594b administration will continue as scheduled and will not be impacted or adjusted based on venetoclax dosing schema or venetoclax-related delays.
- Physicians should leukoreduce with hydroxyurea to reduce the peripheral white blood count to below 10 x 10^9/L prior to the administration of the first dose of venetoclax. If the

WBC is more than 10 x 10^9/L, the venetoclax should not be initiated till the white count is brought down to below 10 x 10^9/L.

- The subject will be admitted to the hospital for at least the first 7 days of the 1st cycle of concomitant azacitidine-venetoclax-DS-1594b therapy (e.g., Day 1 through Day 7) of Cycle 1, potentially admission will be longer if we encounter TLS, infections or other complications that would be better managed in the in-patient setting. To mitigate the risk for tumor lysis syndrome, subjects must be receiving tumor lysis prophylaxis, including hydration (oral, intravenous) and treatment with a uric acid reducing agent (allopurinol, rasburicase) prior to the start of venetoclax therapy and continued during Cycle 1.
  - TLS chemistry tests (potassium, uric acid, creatinine, calcium and phosphorus) will be obtained prior to dosing and 5 to 8 hours after each new venetoclax dose during the venetoclax ramp-up period. TLS chemistry test results will be reviewed by the investigator in real time and prior to the subject receiving the next higher dose of venetoclax to ensure appropriate and timely management. If a subject meets the criteria for clinically significant laboratory or clinical TLS, no additional venetoclax should be administered until resolution of the TLS. Venetoclax interruption for up to 72 hours following transient (< 48 hours) chemical changes and laboratory TLS will be allowed and will not require a dose reduction.
- See Section 6.4 for details of TLS management.
- If azacitidine cycles are delayed, venetoclax therapy may continue on Days 1-21 or Days 1-28 of a 4-week cycle after discussion with the PI or Co-PI and documentation in the medical record.If azacitidine cycles are delayed, DS-1594b may continue without interruption after discussion with the PI or Co-PI and Sponsor, and documentation in the medical record.

5.4.2. Venetoclax Administration

If a dose is missed or vomited, the next dose should not be increased to account for missing a dose. The subject should take the next regular dose at the regularly scheduled time.

Day 1 of each cycle will be counted from the start of the azacitidine infusion. Treatment may be prolonged beyond the planned 28 days of each cycle if the start of the next course of azacitidne is delayed. However, if there are adverse events that mandate treatment interruption or it is considered in the best interest of the subject for safety reasons to interrupt DS-1594b and/or venetoclax therapy, DS-1594b and/or venetoclax administration can be transiently discontinued and re-started as per guidelines in Section 6.

5.5. Phase 2 Cohort D: R/R ALL treated with mini-HCVD with DS-1594b

Subjects will be treated according to the following dose levels:

Table 8: Dose levels for mini-HCVD, and DS-1594b

Dose Level	mini-HCVD	DS-1594b
-1	As published[21,22] mini-HCVD regimen	Lower dose of DS-1594b as recommended by SRC
0 (Starting dose)	As published mini-HCVD regimen	One dose level below the RP2D of DS-1594b from the Phase 1
1	Not applicable	RP2D of DS-1594b from the Phase 1

DS-1594b orally twice daily (or daily, dependent on results from dose escalation phase). In each cycle, DS-1594b will be taken daily continuously.

Variations in dose reductions of the individual chemotherapy or the administration of venetoclax or supportive care dose schedules other than those suggested below are allowed in the best interest of subjects. Such subjects should be discussed with the principal investigator. Dose escalations of chemotherapy above those outlined in the protocol; however, are not allowed. Variations in infusion times due to minor differences in IV bag overfill/underfill and institutional procedure on flushing chemotherapy lines will not result in protocol deviation.

5.5.1. Treatment Overview

DS-1594b will be given orally twice daily (or daily, dependent on results from dose escalation phase) in combination with mini-hyper-CVD. Each cycle is 28 days. Mini-hyper-CVD chemotherapy regimen consists of a total of 8 cycles of intensive chemotherapy with Mini-hyper-CVD alternating with high-dose methotrexate and cytarabine administered approximately every 28 days (or later to allow for recovery from myelosuppression or infection). Subjects will then receive up to 2 years of maintenance with: DS-1594b, vincristine and corticosteroids. Subjects who are responding, and without any major drug-related toxicity, may continue DS-1594b until the end of the 2-year maintenance period.

Cycle 1: Eligible subjects will receive DS-1594b orally twice daily (or daily) in the in-patient setting in combination with Mini-hyper-CVD chemotherapy starting together on Day 1. The subjects will remain hospitalized up until the completion of chemotherapy. The hospital stay may be prolonged in subjects with infections or other issues requiring an in-patient stay. The remainder of the course of DS-1594b can be administered as an out-patient. Dose level 0 is the starting dose level and will be one dose level below the RP2D, and dose level -1 will only be explored if there is excess toxicity with the starting dose. If dose level 0 is tolerated, escalation to the RP2D will be performed as described in Section 3.3. Each cycle is 28 days, however additional days may be needed for subject recovery before starting the next cycle. At the end of Cycle 1, subjects will have bone marrow aspirate/biopsy for response assessment.

Hydroxyurea and/or dexamethasone is allowed prior to the beginning of Mini-hyper-CVD.

Cycles 2 – 8: Subjects will continue DS-1594b orally twice daily (or daily) continuously. Chemotherapy from the mini-hyper-CVD regimen will be administered in the in-patient setting,

starting on Day 1 of each of the Cycles 2 through 8. The subjects may remain hospitalized up until the completion of chemotherapy. Hospital stay may be prolonged for subjects with infections or other issues requiring in-patient stay. The remainder of the course of DS-1594b can be administered as an out-patient.

Upon completion of the 8 courses of the Mini-hyper-CVD regimen, subjects may continue DS-1594b orally twice daily (or daily) continuously of each 28-day cycle for up to 2 years during maintenance. The dose will be according to the dose level administered during Cycles 2 through 8. Subjects may be moved from the intensive chemotherapy phase to the maintenance phase with vincristine, steroids, DS-1594b orally twice daily (or daily) continuously prior to completion of 8 cycles of chemotherapy due to frequent infectious complications, or if the subject is intolerant to the chemotherapy (after discussion with the Principal Investigator). If an early transition to maintenance phase is planned, subjects should receive at least 2 cycles of intensive chemotherapy. The reason for early transition to the maintenance phase must be documented in the medical record.

At any time while enrolled in this trial, subjects may be referred for hematopoietic stem cell transplantation at the discretion of the treating physician. DS-1594b will be discontinued at least 2 days prior to the start of the conditioning regimen for hematopoietic stem cell transplantation.

5.5.2. Mini-Hyper-CVD Regimen

5.5.2.1. General Considerations

- The Mini-hyper-CVD (odd cycles) will alternate with methotrexate and cytarabine (even courses) administered on Day 28 (if count recovery allows) or later day cycle (as count recovery allows).
- Anti-emetic therapy with each course of intensive chemotherapy as needed.
- Filgrastim (G-CSF) will be administered with each course after the completion of chemotherapy. Peg-filgrastim may replace filgrastim (G-CSF). Biosimilars may be used for filgrastim or peg-filgrastim.
- Next cycle may be started when granulocytes > 1.0 x $10^{9/}$L and platelets ≥ 50 x 10^9/L. Cycles may be started with dose reductions prior to full platelet recovery, if the treatment is delayed (e.g., greater than 28 days from last cycle).
- Prophylactic antibiotics may be given with each course until neutrophil recovery to 0.5 x 10^9/L or greater (or other antibiotics if being treated for active infection). Suggestions include: Levaquin 500 mg orally daily; trimethoprim-sulfamethoxazole double-strength one tablet orally twice daily; or other appropriate antibacterial agent. Caspofungin or another appropriate antifungal agent. Valacyclovir 500 mg orally daily; or acyclovir 200 mg orally twice daily; or another appropriate antiviral agent.
- Omit azoles on Day -1, same day, and day after vincristine.
- In general, 8 cycles of chemotherapy (4 Mini-hyper-CVAD, 4 methotrexate plus cytarabine) will be administered in approximately 28-day intervals (depending on the recovery of blood counts. Thereafter, maintenance therapy with DS-1594b in

combination with, vincristine and corticosteroids may be continued for up to 2 years of maintenance. Modifications thought to be in the best interest of the subject are allowed after discussion with the Principal Investigator. Subjects will be followed indefinitely for relapse and survival.

- For subjects with CD20 expression (≥ 20% by flow cytometry) Rituximab 375 mg/m^2 may be added on Days 1 (± 3 days) and 11 (± 3 days) of Cycles 1 and 3, and on Days 1 (± 3 days) and 8 (± 3 days) of Cycles 2 and 4. For subjects receiving rituximab all therapy can be moved back 1 day if needed (i.e., Day 1 starts Day 2, Day 2 starts Day 3, etc.).
- Methotrexate dose regimen will be adjusted as indicated below for pleural effusions.

5.5.2.2. Mini-Hyper-CVD [Odd courses 1, 3, 5, 7]:

- Cyclophosphamide (CTX) 150 mg/m^2 intravenously (IV) over 3 hrs (± 1 hour) every 12 hrs x 6 doses on Days 1, 2, 3
- MESNA 300 mg/m^2/d IV continuous infusion daily for 24 hrs (± 4 hours), starting approximately 1 hour prior to CTX and completing by approximately 12 hrs after the last dose of CTX.
- Vincristine 2 mg IV on Days 1 (± 3 days) and 11 (± 3 days). Vincristine is not myelosuppressive and may be given while subjects are receiving a filgrastim product (G-CSF); no known adverse effects have been observed with the 2 agents given together. Biosimilars may be used for filgrastim or peg-filgrastim.
- Dexamethasone 20 mg IV or orally daily on Days 1 - 4 (± 3 days) and Days 11 - 14 (± 3 days).
- Filgrastim (G-CSF) 10 mcg/kg (rounded) subcutaneously daily (or 5 mcg/kg twice daily) until post-nadir granulocytes > 1.0 x 10^9/L and may be stopped earlier for bone pain or other related toxicity. Minimum time allowed between courses is 14 days. Peg-filgrastim may replace filgrastim (G-CSF) at a dose of 6 mg SQ on Day 5 (± 3 days). Biosimilars may be used for filgrastim or peg-filgrastim.
- For subjects with CD20 expression (≥ 20% by flow cytometry) Rituximab 375 mg/m^2 may be added on Days 1 (± 3 days) and 11 (± 3 days) of Cycles 1 and 3. For subjects receiving rituximab all therapy can be moved back 1 day if needed (i.e., Day 1 starts Day 2, Day 2 starts Day 3, etc.).
- CNS prophylaxis: Methotrexate 12 mg intrathecally (6 mg via Ommaya reservoir) on Day 2 (± 3 days) of Cycles 1 and 3. Cytarabine 100 mg intrathecally on Day 7 (± 3 days) of Cycle 1 and 3 for a total of 8 intrathecal (IT) doses. These are generally given on Cycles 1 - 4 but if one or more IT treatments is missed it can be made up on a later cycle. Administer in 3 to 5 cc of preservative-free normal saline.

5.5.2.3. Methotrexate and Cytarabine [Even courses 2, 4, 6, and 8]:

- Methotrexate (MTX) 50 mg/m^2 IV over 2 hrs (± 1 hour) followed by 200 mg/m^2 over 22 hrs on Day 1. Total duration of administration is 24 hours (2 plus 22 hours) (± 3 hours).
- Cytarabine 0.5 g/m^2 IV over 3 hrs (± 1 hour) every 12 hrs for 4 doses on Days 2 and 3.
- Leucovorin rescue 50 mg IV or oral followed by 15 mg IV or oral every 6 hours for 8 doses (or until MTX level is less than 0.1 µM) beginning 12 hrs (± 3 hrs) post MTX completion (i.e., approximately 36 hours from start of MTX).
- Check MTX levels around time 0h, 24h and 48h post completion of MTX unless methotrexate cleared:
  - if > 20 µM at Time 0, hold cytarabine and repeat level; if continues to be > 20 µM reduce cytarabine to 0.25 g/m^2 IV over 2 hours every 12 hours for 4 doses on Days 2 and 3. Begin leucovorin rescue as described above.
  - if > 1 µM at 24hrs or > 0.1 µM at 48 hours, increase leucovorin rescue to 50 mg IV or oral every 6 hrs until serum methotrexate level is < 0.1 µM. Clearance to levels 0.15 µM or less is acceptable in subjects with normal renal function.
  - Leucovorin rescue may be increased further for elevated methotrexate levels or delayed clearance
- Filgrastim (G-CSF) 10 mcg/kg (rounded) subcutaneously daily (or 5 mcg /kg twice daily) until post-nadir granulocytes ≥ 1.0 x 10^9/L and may be stopped earlier for bone pain or other related toxicity. Minimum time allowed between courses is 14 days (e.g., Day 14). Peg-filgrastim may replace filgrastim (G-CSF) at 6 mg SQ on Day 4 (± 3 days). Biosimilars may be used for filgrastim or peg-filgrastim.
- For subjects with CD20 expression (≥ 20% by flow cytometry) Rituximab 375 mg/m^2 may be added on Days 1 (± 3 days) and 8 (± 3 days) of Cycles 2 and 4. For subjects receiving rituximab all therapy can be moved back 1 day if needed (i.e., Day 1 starts Day 2, Day 2 starts Day 3, etc.).
- CNS prophylaxis: cytarabine 100 mg day 5 + 3 days and methotrexate 12 mg intrathecal (IT) (6 mg via Ommaya reservoir) Day 8 + 3 days of Cycle 2 and 4 for a total of 8 IT doses. These are generally given on Cycles 1- 4 but if one or more IT treatments is missed it can be made up on a later cycle. Administer in 3 to 5 mL of preservative-free normal saline

5.5.3. Intrathecal Treatments

If the subject has been previously treated, and has had prior intrathecal therapy, or prior CNS disease, discuss management of CNS with the Principal Investigator.

If active CNS disease: Consider methotrexate alternating with cytarabine twice weekly until cerebrospinal fluid (CSF) clear; then once weekly for 4 weeks, then every other week for 4

weeks, then monthly for 4 months. Alternative methods of treating CNS disease are allowed if appropriate for the subject (e.g., intrathecal liposomal cytarabine, or others).

Concomitant intrathecal chemotherapy should be avoided, if possible, on the days of administration of intravenous methotrexate and cytarabine (even courses).

5.5.4. Suggested Standard Dose Reductions/Modifications/Discontinuations:

- Vincristine 1 mg IV Days 1 and 11 (50% reduction) if:
  - Bilirubin > 2 mg/dl and ≤ 3 mg/dl
  - Clinically significant grade 2 peripheral neuropathy persisting greater than 2 weeks.
  - Eliminate vincristine for Grade 3-4 peripheral neuropathy, including Grade 3-4 ileus suspected to be related to vincristine, or bilirubin > 3 mg/dL.
- Methotrexate:
  - Consider reduction by 25% to 50% for Grade 3 or worse mucositis with previous methotrexate course.
  - Reduce by 50% for calculated creatinine clearance 10-50 mL/min, if < 10 ml/min, hold methotrexate.
  - Reduce by 25% to 75% for delayed excretion and/or nephrotoxicity with previous methotrexate course.
  - Reduce by 50% for pleural effusion or ascites (drain effusion if possible).
- **Non-hematological toxicity**
  - If Grade 3 or 4 non-hematologic toxicity is at least possibly attributable to DS-1594b, dose interruption of DS-1594b is required. Subjects who experience drug-related Grade 3 non-hematological toxicity may be given a subsequent course one dose level below the previous course, but the subject must have recovered to Grade ≤1 before start of the next course. If a subject has drug-related Grade 4 non-hematological toxicity, he/she may receive a subsequent courses at one reduced dose level after resolution of toxicity to Grade ≤1, only if approved by the PI based on the clinical significance of the toxicity and only if subject has had derived a benefit from the therapy. The dose of DS-1594b can be decreased during a cycle, at the discretion of treating physician and PI, for chronic Grade 2 non-hematological toxicity. Other dose modifications may be considered as clinically indicated with documentation and approval of the PI.
- **Hematologic toxicity**
  - Dose interruptions or modifications will be made for Grade 4 hematological toxicities, only if considered at least possibly related to DS-1594b and not related to underlying disease or the use of chemotherapy, as determined by the treating

physician and the study PI. Subjects with baseline neutropenia or those who have significant bone marrow involvement may be particularly at high risk.

  - If a subject achieves CRi or has a morphologically leukemia free bone marrow after completion of a cycle, DS-1594b may be interrupted from Day 21 for up to 14 days or until recovery of ANC ≥ 0.5 x 10^9/L. The next cycle administration will also be delayed until ANC ≥ 0.5 x 10^9/L. If DS-1594b is interrupted then the next cycle administration of chemotherapy will also be delayed. The next cycle with both DS-1594b and chemotherapy will resume on the same day after the interruption.
  - If a subject presents with new onset Grade 4 neutropenia for more than 1 week during subsequent cycles, unless it is thought to be due to the underlying disease, DS-1594b dosing may be interrupted until ANC recovery to >0.5 x 10^9/L in consultation with the study PI. DS-1594b may be re-initiated at a lower dose per discussion between the treating physician and the study PI.

- Other modifications of drug schedules may be implemented if judged to be in the best clinical interest of the subject after discussion with PI or at the discretion of the treating physician. This includes delays in chemotherapy cycles because of persistent myelosuppression, other side effects, subject request, or other reasons. Missed doses of DS-1594b do not need to be made up.
- Dose reductions exceeding those above or in other agents, e.g., leucovorin, antibiotics, antiemetics, etc., are allowed after discussion with the Principal Investigator.

5.5.5. Maintenance

Maintenance chemotherapy with vincristine, DS-1594b, and prednisone for approximately 24 months beginning at Dose level 0 or lower dose level if prior toxicity required dose reduction of agents (titrate to keep ANC > 0.5 x 10^9/L and platelet count ≥ 30 x 10^9/L) (See Table 9 below):

- Vincristine 2 mg IV on Day 1 approximately every 28 days.
- Prednisone 50 mg orally daily on Days 1 to 5 approximately every 28 days with vincristine.
- DS-1594b continuously, twice daily (or daily) every 28 days at the same dose received during induction/consolidation.
- For DS-1594b dose adjustments, see Section 6.
- Suggested maintenance chemotherapy dose adjustments are in Table 9 below.

Table 9: Maintenance Chemotherapy Dose Adjustments

Level	Vincristine (mg)	Prednisone (mg)
0	2	50
-1	1	40
-2	0	30

- Prednisone
  - Dose should remain at 50 mg unless steroid myopathy or other significant toxicity occurs.
  - Prednisone is not required to be dose adjusted for hyperglycemia.
  - Further reductions beyond what is shown in Table 9 may be allowed if recommended by the treating physician and after discussion with the PI.
- Vincristine
  - Decrease by one dose level for Grade 2 peripheral neuropathy persisting longer than 2 weeks and bilirubin >2 mg/dL and ≤ 3 mg/dL.
  - Discontinue for Grade 3 or greater peripheral neuropathy persisting longer than 2 weeks, including Grade 3-4 ileus suspected to be related to vincristine, or bilirubin > 3 mg/dL.

5.6. Concomitant Medications

5.6.1. Concomitant Medications – Subject Supportive and Safety

The use of any concomitant medication/therapies deemed necessary for subject supportive care and safety are permitted, with the exception of those outlined below. Other leukemia directed therapy including systemic chemotherapy or biologic response modifiers are not permitted during the study except for those specified in the eligibility criteria. A course of radiation therapy is permitted, if in the best interest of the subject and approved by the PI/Co-PI. No other investigational agent for the treatment of AML is allowed during the study. Antiemetics may be used for the prevention or treatment of nausea and vomiting.

Administration of other antineoplastic agents is prohibited for subjects while on this study with the following exceptions:

- intrathecal (IT) therapy for subjects with controlled CNS leukemia at the discretion of the PI,
- Since the effect of DS-1594b may be mediated by differentiation, this effect may be delayed, use of hydroxyurea (any dose) for subjects with rapidly proliferative disease is allowed on study and before the start of study therapy and will not require a washout. These medications will be recorded in the case-report form.

5.6.2. Medications that may prolong the QTc interval

Medications that may prolong QTc interval and have a known risk of inducing Torsades de Pointes should be avoided unless it is vital for the care of the subjects:

- Concomitant use of DS-1594b with drugs known to have a high risk of increasing the QTc interval, and drugs known to increase the QTc interval that are also primarily metabolized by CYP3A should be avoided. See Appendix Table 31.

5.6.3. Concomitant Medications Requiring Caution

- Based on nonclinical assessment, DS-1594b is primarily metabolized by CYP3A. Caution should be exercised with concomitant use of drugs that are moderate inhibitors or inducers of CYP3A (Appendix Table 33). The actual dose, actual start/end date and time of dosing for these concomitant medications should be recorded on the eCRF.
- DS-1594b is shown to inhibit P-gp activity in vitro with estimated IC_{50} value of 1.79 µM. Alternative therapies that are not P-gp substrates should be considered. If concomitant use of DS-1594b and P-gp substrates is unavoidable, separate DS-1594b administration by at least 6 hours before or after the administration of oral narrow therapeutic index P-gp substrates (e.g., digoxin, tacrolimus) to minimize the potential for interactions.
- DS-1594b was shown to inhibit BCRP and OCT1 activities in vitro with estimated IC_{50} value of 1.94 µM and 0.489 µM, respectively. Alternative therapies that are not BCRP or OCT1 substrates should be considered. If concomitant use of DS-1594b and BCRP or OCT1 substrates is unavoidable, caution should be exercised when co-administered with DS-1594b (Appendix Table 33).
- The concurrent use of a H2-antagonist or an antacid is allowed, but the H2-antagonist must be administered 10 hours before or 2 hours after the DS-1594b dose, and the antacid must be administered 2 hours before or 2 hours after the DS-1594b dose.
- Caution is advised with concurrent use of proton pump inhibitors, as exposure of DS-1594b may be reduced. The dose, start/end date of dosing for these concomitant medications should be recorded on the eCRF.
- Caution is advised with concurrent use of ethinyl estradiol containing contraceptives, as in vitro studies of DS-1594b have demonstrated the potential for time-dependent inhibition of CYP3A. If ethinyl estradiol containing contraceptives are used, counsel subjects and monitor for adverse events related to potential increased ethinyl estradiol exposure, such as venous thromboembolism.
- Drugs that should be used with caution are included in Appendix Table 33.

5.6.4. Prohibited Concomitant Medications:

- Co-administration of DS-1594b with drugs that are CYP3A and CYP2C19 substrates known to have narrow therapeutic index should be avoided, unless there are no

alternative therapies or if the drugs are considered by the investigators as vital for the care of the subjects.

- Strong inhibitors or inducers of CYP3A may affect the clearance of DS-1594b, and this may impact its potential efficacy or increase the risk of side effects. Therefore, strong CYP3A inhibitors (e.g., posaconazole, voriconazole) or inducers (e.g., rifampin) should be restricted in the initial stage of the study until DDI assessments have been conducted post-MTD/RP2D determination to characterize this risk.
- P-gp substrates that have narrow therapeutic indices should be avoided.
- Drugs that should be restricted is included in Appendix Table 32.
- For the DDI sub-study with anti-fungal agents, medications known to induce or inhibit CYP3A (moderate/strong inhibitors/inducers) must be discontinued or substituted one week prior to the first dose of DS-1594b, or must be temporarily interrupted during the course of the DDI sub-study of 17 day duration (Appendix Table 31 and Table 34).
- For the food effect sub-study, gastric reducing agents such as proton pump inhibitors should be discontinued one week prior to the first dose of DS-1594b or must be temporarily interrupted during the sub-study of 14-day duration (Appendix Table 34).
- Refer to the package inserts of venetoclax and azacitidine for prohibited concomitant medications related to venetoclax and azacitidine (Cohort C).

5.6.5. Supportive Care Measures

Supportive care measures including blood products, infection prophylaxis and growth factors will be administered according to standard practice at the investigational site (See Recommendations in Table 10).

Table 10: Recommendations for the use of concomitant medications and therapies

Category of Use	Medication	Comment on Use	Restriction on Use
Recommended	Prophylactic antibiotics, antifungal agents, and antiviral agents	Strongly encouraged	Refer to Appendix Table 25, Table 26, and Table 28 for restrictions, and Section 5.3.
	Antiemetic agents	According to standard of care at the site	Avoid QTc prolonging antiemetics (Section 10)
	Hydration (oral, intravenous)	Recommended for tumor lysis syndrome prophylaxis with venetoclax	None

Category of Use	Medication	Comment on Use	Restriction on Use
		administration (Section 6.4)	
Allowed	Oral allopurinol or rasburicase	According to standard of care at the site	None
	Leukapheresis	According to standard of care at the site	Before first dose on Day 1 = 1 only
	Red blood cell transfusion	None	None
	Platelet transfusion	None	None
	White blood cell transfusion	At Investigators discretion according to standard of care at the site	None
	Myeloid growth factors or platelet growth factor	At Investigators discretion according to standard of care at the site	None
	Erythropoietin or darbepoetin	At Investigators discretion according to standard of care at the site	None
	Any other medication for supportive care	At Investigators discretion according to standard of care at the site	None

Consistent with subject safety and comfort, administration of any prescription or over-the-counter drug products other than study medication should be minimized during the study period. Subjects should be discouraged from use of street drugs, herbal remedies, self-prescribed drugs, tobacco products, or excessive alcohol during the clinical study.

If considered necessary for the subject's wellbeing, drugs for concomitant medical conditions or for symptom management may be given at the discretion of the investigator. The investigator's decision to authorize the use of any drug other than study drug should take into account subject safety, the medical need, the potential for drug interactions, the possibility for masking symptoms of a more significant underlying event, and whether use of the drug will compromise the outcome or integrity of the study.

Subjects should be instructed about the importance of the need to inform the clinic staff of the use of any drugs or remedies (whether prescribed, over-the- counter, or illicit) before and during the study.

Recommendations for specific types of concomitant therapies, supportive care; diet and other interventions are as follows:

- Infections secondary to myelosuppression are common in subjects with AML, and may be related to underlying disease, chemotherapy, or both. Therefore, the use of prophylactic antibiotics, antifungal agents, and antiviral agents is recommended according to institutional standards. The use of strong CYP3A inhibitors is not allowed. Therefore, the use of antifungal, antiviral and antibiotics agents listed in Appendix Table 32 are prohibited until DDI assessments based on DDI sub-study have been conducted to fully characterize the risk.
- All ongoing medications and therapies (including herbal products, nutritional supplements, and nontraditional medications) at screening will be considered prior medications. All prior medications (within 30 days of enrollment) and concomitant medications will be recorded in the case report form.
- If a prohibited medication is inadvertently administered/ taken by the subject, the subject may remain on study as long as the prohibited medication is discontinued as soon as feasible. If a prohibited medication is considered essential for the subject well-being, continuation on study with concomitant administration of such medication(s) will need to be discussed with and approved by the principal investigator and sponsor.

6. DOSING DELAYS / MODIFICATIONS / DISCONTINUATIONS FOR POSSIBLY RELATED ADVERSE EVENTS

6.1. Toxicity At Least Possibly Related to Study Drugs

Subjects experiencing unacceptable toxicity at least possibly related to the study drugs should temporarily stop treatment according to the guidelines in the dose adjustment schema.

6.2. Toxicity Grading

Toxicity grading will be according to the NCI CTCAE, v5.0. To prevent unnecessary morbidity, the following guidelines for dose adjustment for toxicities at least possibly related to study drugs are recommended.

6.2.1. Dose Adjustments for Hematological Adverse Events (AE) Considered at Least Possibly Related to Study Drugs

Dose reduction/interruption/discontinuation decisions should be based on the CTCAE version 5.0 and the guidelines provided below.

Subjects with acute leukemias usually present with abnormal peripheral blood counts at the time therapy is started, and myelosuppression is an expected event during the course of therapy for acute leukemia. Thus, subsequent courses may be administered regardless of peripheral blood counts during the first 3 cycles and/or in the presence of residual leukemia. After Cycle 3, treatment interruptions and dose adjustments may be considered according to the following guidelines when there is no evidence of active leukemia:

- Subjects with a response (no evidence of any residual leukemia on bone marrow and/or peripheral blood by morphology or flow-cytometry) will have dose modifications for hematological toxicities as specified in the azacitidine and venetoclax PIs and in Section 5.4.1 and Section 5.4.2. See Table 11 for recommended dose modifications for DS-1594b for hematologic adverse events that are considered at least possibly related (see Section 5.1 for preliminary dose levels).
- If there are persistent peripheral blood blasts, or the bone marrow shows ≥ 5% blasts or any evidence of residual leukemia by morphology or flow, treatment may be continued regardless of neutrophil and platelet count with supportive care as needed. Dose-interruptions of individual drugs in these subjects should be considered on an individual case-by-case basis and discussed with the PI and sponsors.
- Investigators should, whenever possible, determine which medication is causing the toxicity and interrupt or dose reduce, as applicable.

Table 11: DS-1594b Dose Adjustments for Neutropenia At Least Possibly Related to Study Drug

Grade	Occurrence	Dose Modification
1 or 2	Any	No modification
3 (without infection or febrile neutropenia)	Any	No modification
≥ 3 (with infection or febrile neutropenia) or 4	1st	Interrupt dosing until resolution to ≤ Grade 2 or baseline<br>• If recovery occurs ≤14 days of holding DS-1594b: resume dosing at the same dose level the subject was on at the time of the interruption<br>• If recovery occurs >14 days of holding DS-1594b: resume dosing at one dose level lower than the dose level the subject was on at the time of the interruption (Dose level -1)

Grade	Occurrence	Dose Modification
	2nd	Interrupt dosing until resolution to ≤ Grade 2 or baseline<br>• If recovery occurs ≤14 days of holding DS-1594b: resume dosing at the same dose level the subject was on at the time of the interruption<br>• If recovery occurs >14 days of holding DS-1594b: resume dosing at one dose level lower than the dose level the subject was on at the time of the interruption (Dose level -1 or -2, depending on the dose level the subject was on at the time of the interruption)
	3rd	• If the subject is on Dose level -2 at the time of the event, permanently discontinue therapy.<br>• If the subject is not on Dose level -2 at the time of the event, interrupt the dosing until resolution to ≤ Grade 2 or baseline and resume dosing at one dose level lower than the dose level the subject was on at the time of the interruption (Dose level -1 or -2, depending on the dose level the subject was on at the time of the interruption).
	4th	• Permanently discontinue therapy.

Note: Treatment interruptions and dose modifications other than the ones mentioned above can be considered after discussion with the PI and proper documentation of the rationale.

6.2.2. Dose Adjustments for Non-Hematologic AEs Considered at Least Possibly Attributable to Study Drugs

Investigators should, whenever possible, determine which medication is causing the toxicity and interrupt or dose reduce azacitidine, venetoclax, and/or interrupt DS-1594b, see Table 12.

Dose reductions of azacitidine will be as follows: Baseline dose level: 75 mg/m^2 x 7 days, dose level -1 of azacitidine: 50 mg/m^2 x 7 days, dose level -2: 37.5 mg/m^2 x 7 days, dose level -3: 25 mg/m^2 x 7 days. Further reductions or modifications to schedule beyond what is shown above or alternative reductions (e.g., 75 mg/m^2 x 5 days) may be allowed if deemed in the subject's best interest by the treating physician after approval from the PI and Sponsors.

Dose reductions of venetoclax will be as follows: Baseline dose level of venetoclax: 400mg, dose level -1 of venetoclax: 200mg, dose level -2: 100mg, dose level -3, 50mg. Alternatively, the duration of venetoclax administration can be decreased (e.g., decrease from 28-days per cycle to 21-days per cycle to 14-day per cycle to 10 days per cycle) rather the dose being reduced.

Dose reductions for DS-1594b will be allowed if warranted by Investigator and after discussion and approval by PI and Sponsors and will follow the Phase 1 doses used for DS-1594b (Table 7).

Table 12: Dose Adjustments of Azacitidine, Venetoclax, and/or DS-1594b for Non-Hematologic Possibly Related AEs, Clinically Significant in the Opinion of the Investigator

Grade	Occurrence	Dose modification
1 or 2	Any time	No dose reduction
3<br><br>(Persistent Grade 2: Consider similar dose adjustments if persistent and not responding to optimal management in the opinion of PI and treating physician)	1st and 2nd time	Hold suspected drug.<br>-Resume the held drug at prior dose if recovery to ≤ Grade 1 occurs within 14 days.<br>-If toxicity persists for 15-28 days, hold the drug and resume at prior dose if recovery to ≤ Grade 1 OR resume the drug at ONE dose level below current dose if recovery to ≤ Grade 2.<br>-If toxicity persists for >28 days, hold the drug; resume the drug at ONE dose level below current dose if recovery to ≤ Grade 2.<br><br>Dose re-escalation to prior dose of the drug is permitted in accordance with the dose-escalation guidelines in Section 5 .
	3rd time	Hold suspected drug.<br>Follow until toxicity ≤ Grade 2.<br>Resume the held drug at ONE dose level below current dose.<br>Dose re-escalation of the drug to prior dose is permitted in accordance with the dose-escalation guidelines outlined below Subjects who are experiencing ongoing dose delays >8 weeks due to unresolved grade ≥3 adverse events should be taken off treatment.
	4th time	Discontinue therapy
4	Any time	Discontinue therapy

Note: For dose adjustments to DS-1594b for QTc Interval Prolongation or Acute Differentiation Syndrome, refer to Table 13 and Table 14.

Table 13: Dose Adjustment for QTc Interval Prolongation

<table>
<tr><td colspan="2">For all QTc interval prolongations (average of the triplicate 12-lead readings or by Holter): Check serum electrolyte levels (including magnesium, potassium, and calcium) and correct any abnormalities. If possible, stop any medications that may prolong the QTc interval.

Subjects who experience Grade ≥2 QTcF prolongation and undergo dose interruption and/or reduction must be monitored closely with ECGs, performed twice weekly for the first week of the QTcF prolongation and then weekly thereafter until the QTcF prolongation is resolved.</td></tr>
<tr><td>Grade 2: QTcF (average of triplicate ECG readings) > 480ms and ≤ 500ms</td><td>Reduce the dosage of one dose level without interrupt the administration
To resume the treatment at the previous dose level if the QTcF has decreased to within 30 ms of baseline or <450 ms</td></tr>
<tr><td>Grade 3: QTcF (average of triplicate ECG readings) > 500ms for the first time; >60 ms change from baseline</td><td>Interrupt DS-1594b administration.
If QTcF returns to within 30 ms of baseline or <450 ms within 14 days, study drug administration may be resumed at ONE dose level below the current dose.</td></tr>
<tr><td>QTcF (average of triplicate ECG readings) > 500ms repeatedly</td><td>Permanently discontinue DS-1594b administration if QTcF recurs despite appropriate dose reduction and correction/elimination of other risk factors (e.g., serum electrolyte abnormalities, discontinuation of a concomitantly administered drug susceptible to prolong QT)</td></tr>
<tr><td>Grade 4: Torsade de pointes or polymorphic ventricular tachycardia or signs/symptoms of serious arrhythmia</td><td>Permanently discontinue DS-1594b administration</td></tr>
</table>

Table 14: Dose Modification for Clinically Suspected Acute Differentiation Syndrome

<table>
<tr><th>Occurrence</th><th>Action</th><th>DS-1594b dose modification</th></tr>
<tr><td rowspan="2">1st</td><td rowspan="2">Interrupt If severe pulmonary symptoms (requiring intubation or ventilatory support), and/or renal dysfunction persisting more than 48 hours after initiation of corticosteroids
Resume when until signs and symptoms no longer severe (i.e., improve to Grade 2* or lower);
Start treatment with systemic corticosteroids</td><td>If signs and symptoms improve to Grade 2* or lower within 7 days, resume at ONE dose level below current dose.

Escalate back to current dose after 7 days, if the reduced dose is tolerated after.</td></tr>
<tr><td>If syndrome persists for ≥7 days despite supportive management, discontinue permanently.</td></tr>
<tr><td>2nd</td><td>Discontinue DS-1594b permanently; start systemic corticosteroid treatment</td><td>Not applicable.</td></tr>
</table>

*Grade 1 is mild, Grade 2 is moderate, Grade 3 is serious, Grade 4 is life-threatening.

6.2.3. DS-1594b AEs of Special interest (AESI)

6.2.3.1. QTc Prolongation, Torsade de Pointes, and Other Ventricular Arrhythmias

Subjects who experience >480 ms QTcF prolongation and undergo dose interruption and/or reduction must be monitored closely with ECGs, performed twice weekly for the first week of the QTcF prolongation and then weekly thereafter until the QTcF prolongation is resolved (Table 13).

QTcF prolongation of Grade 3 or higher, either serious or non-serious and whether or not causally related, must be recorded as AE or SAE in the Electronic Data Capture (EDC) system within 24 hours of awareness of the central ECG laboratory reading, with the investigator's assessment of seriousness, causality, and a detailed narrative. The central ECG reading confirming QTcF prolongation ≥ Grade 3 is to be recorded in the EDC system.

Regarding the detailed narrative(s):

- Serious AESI: The narrative will be captured directly in the EDC system.
- Non-Serious AESI: The narrative will be captured on a form outside of the EDC system (paper form).

6.2.3.2. Combined Elevations of Aminotransferases and Bilirubin

Combined elevations of aminotransferases and bilirubin, either serious or non-serious and whether or not causally related, meeting the laboratory criteria of a potential Hy's Law case [ALT or AST ≥3 × ULN with simultaneous TBL ≥2 × ULN] should always be recorded as an AE or SAE within 24 hours of awareness, with the investigator's assessment of seriousness, causality, and a detailed narrative.

Regarding the detailed narrative(s):

- Serious AESI: The narrative will be captured directly in the EDC system.
- Non-Serious AESI: The narrative will be captured on a form outside of the EDC system (paper form).

For liver elevation, workup elevations meeting FDA DILI criteria below:

Elevation of liver enzymes that meet the criteria below should be investigated and the cause identified when possible:

- ALT >8 × ULN; or
- AST or ALT >5 x ULN for more than two weeks; or
- ALT or AST >3 x ULN, but not reaching the limits in the above criteria, in combination with clinical symptoms suggestive of hepatitis; or
- ALT or AST >3 x ULN with TBL >2 x ULN.

Once the cause of liver enzyme elevation is identified, remove or treat the contributing cause.

If the above criteria are met and the elevation is considered to be related or possibly related to DS-1594b, dosing should be interrupted.

Liver enzyme level testing will be repeated at least weekly, or more frequently, based on degree of hepatic laboratory abnormality. If the liver enzyme levels return to baseline levels, DS-1594b may be resumed at ONE dose level below the current dose level. If toxicity does not improve/resolve within 28 days, then DS-1594b will be discontinued. Upon resumption of DS-1594b, if liver enzyme elevations recur, treatment must be permanently discontinued.

6.2.4. Management of AESI

6.2.4.1. QTc Prolongation, TdP, and Other Ventricular Arrhythmias

Electrolytes (potassium, calcium, and magnesium) should be checked and supplementation given to correct any values outside the normal range.

Concomitant medications should be reviewed to identify and, if appropriate, discontinue any medication with known QT prolonging effects (Table 31).

Subjects who experience >480 msec QTcF prolongation and undergo dose interruption and/or reduction must be monitored closely with ECGs, performed twice weekly for the first week of the QTcF prolongation and then weekly thereafter until the QTcF prolongation is resolved.

6.2.4.2. Combined Elevations of Aminotransferases and Bilirubin

Combined elevations of aminotransferases and bilirubin, either serious or non-serious and whether or not causally related, meeting the laboratory criteria of a potential Hy's Law case [ALT or AST ≥3 x ULN with simultaneous TBL ≥2 x ULN] should always be recorded as an AE or SAE within 24 hours of awareness, with the Investigator's assessment of seriousness, causality, and a detailed narrative. Subjects will be monitored as described in Section 8.10. Evaluation may include the following depending on the clinical situation:

- Medical history and physical exam, including focus on medications and substances used: alcohol, acetaminophen, azole antifungals, change in medication dosages, new medications added, over the counter medication use and recreational drug use. Check for change in diet or use of dietary supplements;
- Abdominal ultrasound;
- Hepatitis A, B, C, and E screening (anti-hepatitis A virus immunoglobulin M, hepatitis B surface antigen, anti-hepatitis C virus plus viral titer, and evaluation for Hepatitis E), antinuclear antibody and anti-Smith antibody, cytomegalovirus, Epstein Barr virus;
- Additional evaluations as deemed appropriate by the Investigator to exclude other causes of liver enzyme and bilirubin elevations;
- All laboratory results, including local laboratory reference ranges are to be recorded.

6.3. CYP3A and P-gp Inhibitors: Dose modifications Venetoclax

Venetoclax should be administered at 50% dose reduction in the setting of moderate CYP3A inhibitor and at 75% dose reduction in the setting of strong CYP3A inhibitor for the duration of co-administration. The venetoclax dose will be reduced to 70mg by mouth once daily if subject is on posaconazole.

In the event the co-administered CYP3A inhibitor is discontinued, the assigned venetoclax dose should be resumed 3 days after discontinuation.

Every effort should be made to adhere venetoclax dose reduction. Variations in schedule of events such as late/missed interventions that do not affect the rights and safety of the subject will not be considered as deviations.

P-gp substrates: Concomitant use of venetoclax increases Cmax and AUCinf of P-gp substrates, which may increase toxicities of these substrates (please see Venetoclax US prescribing information). Avoid concomitant use of venetoclax with a P-gp substrate. If a concomitant use is unavoidable, separate dosing of the P-gp substrate at least 6 hours before venetoclax.

6.4. Tumor Lysis Prophylaxis (TLS)

The venetoclax dose titration scheme utilized in the AML studies performed to date to mitigate the risk of Tumor Lysis Syndrome (TLS) will be employed. All subjects will be hospitalized for the entirety of the venetoclax dose escalation starting at least on Day 1 of treatment initiation and until 24 hours after the completion of venetoclax dose escalation. During Cycle 1, venetoclax will be dose escalated daily to the goal dose regimen of 400 mg daily. Subjects will receive 100 mg on Day 1, 200 mg on Day 2, and 400 mg on Day 3 and onwards, or adjusted dose if on concomitant azoles (discussed below in Section 6.7).

To mitigate the risk for TLS, subjects must be receiving tumor lysis prophylaxis, including hydration (oral, intravenous) and treatment with a uric acid reducing agent (allopurinol, rasburicase) prior to start of venetoclax and at least during the first cycle of therapy.

TLS chemistry tests (potassium, uric acid, creatinine, calcium and phosphorus) will be obtained prior to dosing and 5 to 8 hours after each day of a new venetoclax dose. TLS chemistry test results will be reviewed by the investigator in real time and prior to the subject's next dose to ensure appropriate management. If a subject meets the criteria for clinically significant laboratory or clinical TLS, no additional venetoclax should be administered until resolution. Venetoclax interruption for up to 72 hours following transient (< 48 hours) chemical changes and laboratory TLS will be allowed and will not require a dose reduction.

6.5. Differentiation Syndrome

DS-1594b may result in terminal differentiation of leukemic blast cells in subjects with relapsed or refractory disease, which may be associated with the development of differentiation syndrome (also known as retinoic acid syndrome) and may be life-threatening or fatal if not treated.

It is important to promptly recognize the signs and symptoms of differentiation syndrome and implement appropriate treatment. Symptoms of differentiation syndrome could include dyspnea, unexplained fever, peripheral edema, unexplained hypotension, weight gain >5 kg, pulmonary

infiltrates, pleuro-pericardial effusion, acute renal failure, musculoskeletal pain, and hyperbilirubinemia. Less commonly, differentiation syndrome might present with pulmonary hemorrhage or acute febrile neutrophilic dermatosis.

The diagnosis of differentiation syndrome requires the presence of at least 2 of the following signs or symptoms: dyspnea, unexplained fever ≥38°C, weight gain >5 kg, unexplained hypotension, acute renal failure, or a chest radiograph demonstrating pulmonary infiltrates or pleuro-pericardial effusion[36] or multiple organ dysfunction.[37] No single sign or symptom is sufficient to make a diagnosis of the syndrome. It is important to rule out other conditions with similar symptoms, such as sepsis, pneumonia, heart failure, pulmonary embolism, and diffuse alveolar hemorrhage.

For subjects at an increased risk for severe differentiation syndrome (white blood cell count > 5 x 10^9/L or serum creatinine > 1.4 mg/dL), consider the administration of prophylactic systemic glucocorticoids.[38] For subjects with a white blood cell count > 10 x 10^9/L, administer hydroxyurea 1-3g per day.

Subjects with suspected differentiation syndrome should promptly start treatment with intravenous or oral dexamethasone 10 mg every 12 hours or equivalent and hemodynamic monitoring until improvement. If symptoms do not improve after 24 hours of therapy, increase dexamethasone dosing to 10 mg every 6 hours. If severe pulmonary symptoms (requiring intubation or ventilatory support), and/or renal dysfunction persisting more than 48 hours after initiation of steroids, interrupt DS-1594b until signs and symptoms no longer severe (Table 14). Hospitalization for subjects with severe pulmonary or renal manifestation is recommended.

6.6. Meals and Dietary Requirements

Each dose of venetoclax should be taken with approximately 240 mL of water within 30 minutes after the completion of a meal, preferably breakfast.

Subjects should not consume grapefruit or grapefruit products, Seville oranges, or Star fruit within the 3-day period prior to the first DS-1594b or venetoclax administration and until the last day of DS-1594b or venetoclax is completed due to possible CYP3A-mediated metabolic interaction.

6.7. Modifications of Dose Schedules Other Than the Above Will Be Allowed Within the Following Guidelines

- Dose adjustments by more than 1 dose level at a time for azacitidine or venetoclax (e.g., from azacitidine 75 mg/m^2 to 25 mg/m^2) can be considered when judged in the best interest of the subject (e.g., severe myelosuppression) when toxicity has resolved. The reason for this reduction will be discussed with the PI or Co-PI and documented in the medical record.
- A subject who has had a dose reduction because of any of the reasons mentioned above may have their dose escalated provided the subject has remained free of toxicity requiring dose adjustments as defined above in Table 12 for at least 1 month. Escalation will be made by 1 dose-level increment only, and not more frequent than

every month. The dose of any agent must not exceed the RP2D dose for that agent in this protocol (i.e., azacytidine dose cannot exceed 75mg/m^2 x 7 days, venetoclax dose cannot exceed 400 mg/day, and established RP2D for DS-1594b from Phase 1).

- Treatment interruptions and dose modifications other than the ones mentioned above can be considered after discussion with the PI and proper documentation of the rationale. This includes delays in chemotherapy cycles because of persistent myelosuppression, other side effects, subject request, or other reasons. Missed doses of DS-1594b do not need to be made up. Dose adjustment/delay/interruptions of only one of the agents is permissible if the toxicity is most likely judged to be related to one of the agents by the investigator (e.g., in subjects with Differentiation Syndrome (Table 14) this would be likely secondary to the DS-1594b, in subjects with neutropenia this would be likely secondary to azacitidine and venetoclax). Subjects in whom one agent is interrupted/discontinued for potential toxicity may be able to continue treatment on protocol if it is in the best interest of the subject to continue on protocol.

6.8. Treatment Interruptions

Treatment interruptions and dose modifications or delays other than the ones mentioned above can be considered after discussion with the PI or Co-PI and clear documentation of the rationale in the medical records.

6.9. Intra-Patient Dose Escalation (IPDE)

Intra-patient dose escalation (IPDE) of DS-1594b is permitted for subjects enrolled in the dose escalation portion of the study who may benefit from IPDE after approval from the PI or Co-PI, provided the subject meets all of the following criteria:

- The subject has failed to achieve a complete remission (CR) or has experienced disease progression on treatment with DS-1594b,
- The subject has not experienced a DLT or treatment related adverse event requiring DS-1594b dose reduction or interruption.

The dose may be escalated up to a dose level that has previously been established to be safe and tolerable in at least 3 DLT evaluable subjects (for example, a subject enrolled at 50mg BID may be dose escalated to 70mg BID only after the 70mg BID dose level has been declared safe and tolerable in at least 3 DLT evaluable subjects).

Additionally, a subject may be switched to an alternative DS-1594b dosing regimen (BID vs. QD) once they have completed the DLT evaluation period in Phase I, if that dosing regimen has been found to be safe and tolerable in at least 3 DLT evaluable subjects (for example, from 50 mg BID to 100 mg QD).

7. AGENT FORMULATION AND PROCUREMENT

7.1. Potential Indications and Use

DS-1594b is being developed for the treatment of relapsed or refractory AML or ALL with MLL1-r or AML with NPM1-mutations.

7.2. Ongoing Clinical Trials

This is the first clinical study for DS-1594b. There are no other ongoing studies.

7.3. Dosing and Administration

See Section 5.1.2 for DS-1594b dosing and administration information.

7.4. Packaging and Labeling

DS-1594b is packaged as 35 tablets per bottle, in high-density polyethylene (HDPE) bottles with desiccant. The labeling complies with the requirements of the applicable regulatory agencies.

7.5. Study Drug Description and Preparation

DS-1594b tablets are formulated in 3 strengths: 20 mg, 50 mg, and 200 mg. The dose strength of the tablet is expressed as the free form of DS-1594b. DS-1594b tablets are supplied as grayish-red, film-coated round-shaped tablets (20 mg) and grayish-red, film-coated oblong-shaped tablets (50 mg and 200 mg).

DS-1594b is supplied as tablets that need no further preparation at the study sites.

7.6. Study Drug Storage

DS-1594b must be stored appropriately in a locked cabinet/room with limited and controlled access under the recommended storage conditions. The required storage conditions are provided in the investigational medicinal product labeling.

NOTE: *If storage conditions go outside of the recommended storage conditions, the Study Center must not dispense the affected supplies (affected supplies should be placed in quarantine). Proper notification processes must be followed immediately after receipt of DS-1594b identified as having a temperature excursion.*

7.7. Study Drug Accountability

A Drug Accountability Record must be kept current and must contain the dates and quantities of study drug received, the subject's identification number and/or initials or supply number (as applicable), the date and quantity of DS-1594b dispensed and remaining (in tablets), as well as the initials of the dispenser.

Azacitidine: It is commercially available and will be handled as per the packet insert and standards in the site pharmacy.

Venetoclax: It is commercially available and will be handled as per the packet insert and standards in the site pharmacy

International Non-proprietary name venetoclax (formerly ABT-199)

Manufacturer Abbvie/Genentech

Dose 70 - 400 mg daily

Route of Administration oral

Formulation Capsule formulation (10 mg, 50 mg and 100 mg)

Venetoclax will be obtained from commercial source. For further details, please refer to the prescribing information (Appendix G).

Mini-HCVD: It is commercially available and will be handled as per the packet insert and standards in the site pharmacy. cyclophosphamide, vincristine, methotrexate, cytarabine, rituximab, MESNA, filgrastim, peg-filgrastim, dexamethasone, and prednisone will be obtained from commercial source. Biosimilars may be used for filgrastim or peg-filgrastim. For further details on each drug, please refer to the prescribing information .

Disposition of unused drug: all unused drug will be disposed of per institutional guidelines and procedures.

Variations in infusion times of drugs due to minor differences in IV bag overfill/under fill and institutional procedure on flushing chemotherapy lines will not result in protocol deviation. All infusion times are considered approximate.

For further details on drug formulation, reconstitution, administration, infusion related instructions, concern and plan of management for infusion related topics please the respective agents IB or package insert.

8. SUBJECT EVALUATION

Every effort will be made to adhere to the schedule of events and all protocol requirements.

8.1. Schedule of Assessments

All Assessment Schedule Tables are in the Appendix:

Phase 1<br>Dose Escalation	Lead-In Period	Table 35
	Post Lead-In Period<br>(28-day Cycles)	Table 36
Phase 2<br>Dose Expansion	Cohorts A & B	Table 37
	Cohort C	Table 38
	Cohort D	Table 39

8.2. Screening

The Screening period begins up to 14 days prior to treatment. It is anticipated that subjects will recently have had some, or all, of the screening tests done as part of routine care outside the auspices of this study. Results of some tests done as part of routine care, outside the auspices of this study, may be used to qualify the subject. All pretreatment studies should be obtained within 14 days of first dose, unless otherwise stated. Written informed consent must be obtained before any study-specific procedure is performed.

All study procedures, risks, and benefits must be explained to the subject and/or their legal guardians; the Study Informed Consent Form must be reviewed with the subject and/or their legal guardians; all the subject's and/or their legal guardians' questions must be answered; the subject and/or their legal guardians must sign the Informed Consent Form. Subjects and/or their legal guardians must receive a copy of their Informed Consent Form.

All subjects will be assigned a unique identification number upon signing the informed consent form.

At screening, the following will be performed:

- A complete history and physical, medical and acute leukemia disease history (including classification per the latest WHO criteria for AML or ALL), disease state (primary refractory, untreated relapse, refractory relapse), number of prior lines of therapy, best response to most recent line of therapy, duration of most recent remission (if applicable), prior medications, non-drug therapies, and radiotherapy within 28 days of Cycle 1 Day 1, prior transfusions (red blood cell and platelet) within 28 days of Cycle 1 Day 1, concomitant medications, non-drug therapies, and radiotherapy, and performance status.
- Complete blood count (CBC), platelet count, differential (differential can be omitted if WBC is $\leq 0.4 \times 10^9$/L), international normalized ratio (INR), fibrinogen, and prothrombin time (PT)/partial thromboplastin time (PTT).

- Serum chemistry panel (including creatinine, electrolytes, glucose, uric acid, calcium, magnesium, BUN).
- Hepatic panel (AST, ALT, Alk phosphatase, total bilirubin [fractionated])
- Creatinine Kinase (fractionated if > ULN)
- Troponin
- Ferritin
- Lactate dehydrogenase (LDH)
- C-reactive protein (CRP)
- Cytokine panel including interleukin 6 (IL-6), interferon gamma (IFN-γ), and tumor necrosis factor alpha (TNF-α)
- Alpha-Melanocyte stimulating hormone (alpha-MSH)
- Fasting morning adrenocorticotropic hormone (ACTH), aldosterone, renin, and cortisol levels. If results are abnormal, consult an endocrinologist. Perform an ACTH stimulation test if adrenal insufficiency is suspected.
- Urinalysis (by dipstick) to include appearance, bilirubin, blood, specific gravity, glucose, ketones, pH, and protein.
- Pregnancy test (urine or plasma) in females of childbearing potential should be performed within 72 hours before initiation of protocol therapy.
- Bone marrow aspirate/biopsy
  - Cytogenetics and molecular tests for MLLr or NPM1m performed by MDACC laboratories must be obtained
- Height, weight, and BSA calculated by the Mosteller formula.
- HIV antibody test (if required by local regulations)
- Hepatitis B (HBsAG) test, and Hepatitis C antibody test
- Blood oxygen levels (SpO2).
- Vital signs, including blood pressure, pulse rate, respiratory rate, and temperature (taken 5 min prior to the electrocardiogram [ECG]).
- Triplicate Electrocardiogram (ECG) by 12-lead or Holter measurement at baseline. See Section 8.5 for the details of ECG monitoring.
- Pretreatment correlative studies (Section 8.6).
- MUGA or Echocardiogram

8.3. Evaluation During Treatment

8.3.1. Lead-In Period (7-day step dosing intervals)

- Physical exam at the start of each lead-in dosing step (± 4 days)
- Hematology/coagulation labs: CBC with differential, platelet count, INR pre-dose on Day 1 and at least 3 times per week
- Fibrinogen and prothrombin time (PT)/partial thromboplastin time (PTT) labs pre-dose on Day 1 and at least 2 times per week.
- Serum chemistry labs: serum creatinine, electrolytes, glucose, uric acid, calcium, magnesium, BUN pre-dose on Day 1 and at least 3 times per week.
- Hepatic labs: AST, ALT, alkaline phosphatase, total bilirubin, direct bilirubin, and indirect bilirubin pre-dose on Day 1 and at least 3 times per week.
- Creatinine kinase (fractionated if > ULN) pre-dose on Day 1 and at least once weekly.
- Urinalysis pre-dose on Day 1 and monthly during treatment.
- Pregnancy test (urine or plasma) in females of childbearing potential should be performed monthly during treatment.
- Ferritin pre-dose on Day 1 and at least 2 times per week.
- Lactate dehydrogenase (LDH) pre-dose on Day 1 and at least 2 times per week.
- C-reactive protein (CRP) pre-dose on Day 1 and at least 2 times per week.
- Cytokine panel including interleukin 6 (IL-6), interferon gamma (IFN-γ), and tumor necrosis factor alpha (TNF-α) pre-dose on Day 1 and at least 2 times per week.
- Troponin during treatment as clinically indicated.
- If lead-in period is ≥ 21 days, an optional bone marrow aspiration/biopsy and/or periferal blood sample may be collected at the end of the lead-in period (± 4 days) for the purpose of correlative studies. Bone marrow tests can be ordered more frequently if mandated by development of peripheral blood counts. No repeat bone marrow is necessary if nonresponse or progressive disease can be unequivocally diagnosed from peripheral blood tests or, in subjects with a WBC < 0.3 if the bone marrow test is considered noncontributory by the investigator at any time point.

8.3.2. Target Dose or Stable Dose after Lead-In (Regular 28-day cycles begin)

- Physical exam at the start of each cycle (± 4 days).
- Hematology/coagulation labs: CBC with differential, platelet count, INR pre-dose on Cycle 1, Day 1 and at least 3 times per week for the first cycle, then 2 times per week for Cycles 2 and 3, then one to two times per week in subsequent cycles (differential can be omitted if WBC is ≤ 0.5 x10^9/L). In addition, fibrinogen and prothrombin time

(PT)/partial thromboplastin time (PTT) labs pre-dose on Cycle 1, Day 1 and at least 2 times per week in Cycle 1. Lower frequency of laboratory evaluations may be considered on a case by case basis after Cycle 6 in subjects in remission, after discussion and approval from PI (e.g., at least once every 2 weeks).

- Serum chemistry labs: serum creatinine, electrolytes, glucose, uric acid, calcium, magnesium, BUN pre-dose on Cycle 1, Day 1 and at least 3 times per week for the first cycle, then 2 times per week for Cycles 2 and 3, then one to two times per week in subsequent cycles. Lower frequency of laboratory evaluations may be considered on a case by case basis after Cycle 6 in subjects in remission, after discussion and approval from PI (e.g., at least once every 2 weeks)
- Hepatic labs: AST, ALT, alkaline phosphatase, total bilirubin, direct bilirubin, and indirect bilirubin pre-dose on Cycle 1, Day 1 and at least 3 times per week for the first cycle, then 2 times per week for Cycles 2 and 3, then one to two times per week in subsequent cycles. Lower frequency of laboratory evaluations may be considered on a case by case basis after Cycle 6 in subjects in remission, after discussion and approval from PI (e.g., at least once every 2 weeks).
- Creatinine kinase (fractionated if > ULN) pre-dose on Cycle 1, Day 1 and at least once weekly in Cycles 1-3, then monthly. Lower frequency of laboratory evaluations may be considered on a case by case basis after Cycle 6 in subjects in remission, after discussion and approval from PI.
- Urinalysis pre-dose on Cycle 1, Day 1 and monthly during treatment.
- Pregnancy test (urine or plasma) in females of childbearing potential should be performed monthly during treatment.
- Tumor lysis syndrome chemistry tests (potassium, uric acid, creatinine, calcium and phosphorus) will be obtained same day prior to dosing and 5-8 hours on each day after a new venetoclax dose during cycle 1 (Cohort C).
- Ferritin pre-dose on Cycle 1, Day 1 and at least 2 times per week in Cycle 1.
- Lactate dehydrogenase (LDH) pre-dose on Cycle 1, Day 1 and at least 2 times per week in Cycle 1.
- C-reactive protein (CRP) pre-dose on Cycle 1, Day 1 and at least 2 times per week in Cycle 1.
- Cytokine panel including interleukin 6 (IL-6), interferon gamma (IFN-γ), and tumor necrosis factor alpha (TNF-α) pre-dose on Cycle 1, Day 1 and at least 2 times per week in Cycle 1.
- Troponin during treatment as clinically indicated.
- Plasma alpha-melanocyte stimulating hormone (alpha-MSH), fasting morning adreno-corticotropic hormone (ACTH), aldosterone, renin, and cortisol levels on Cycle 1 Day 28 (± 4 days), then once every 3 cycles beginning at Cycle 3 (e.g., Cycles 3, 6, 9) and at End of Treatment. If results are abnormal, consult an

endocrinologist. Perform an ACTH stimulation test if adrenal insufficiency is suspected.

- Bone marrow aspiration/biopsy on Cycle 1 Day 28 (± 4 days), then on Day 28 (± 4 days) of Cycles 2, 3, 6, 9 and progression. Bone marrow tests can be ordered more frequently if mandated by development of peripheral blood counts. No repeat bone marrow is necessary if nonresponse or progressive disease can be unequivocally diagnosed from peripheral blood tests or, in subjects with a WBC < 0.3 if the bone marrow test is considered noncontributory by the investigator at any time point. For subjects in Cohort C, the initial bone marrow aspiration/biopsy in Cycle 1 will be on Day 21 (± 4 days).
- For subjects that remain on study with no significant toxicity for more than 6 months, subsequent evaluations during study may be modified after discussion with the principal investigator. These include a decrease in frequency of bone marrow aspiration and biopsy to every 6-12 months (or as clinically indicated), correlative studies to every 6-12 months (or suspension of sample collection for correlative studies), other laboratory tests to once every cycle.

8.4. PK Assessment

Blood samples for PK analysis of DS-1594b and metabolites (if appropriate) will be collected during the study from all subjects receiving DS-1594b. PK parameters using standard non-compartmental method will be generated. Plasma concentration-versus-time data will be summarized using summary statistics by dose/study day/time

- On Cycle 1 Day 1 and PK sampling days during lead-in period (for cohorts with lead-in period)(dose escalation and dose expansion): Cmax, Tmax, Tlast, AUClast, AUCtau, AUCinf*, Kel*, t1/2*, CL/F*, Vz/F*
- On Cycle 1 Day 8 (dose escalation and dose expansion): Cmax, Cavg, Ctrough, Tmax, Tlast, AUClast, AUCtau, Kel*, t1/2*, CLss/F, Vz/F*, AR**, t1/2,ar***
- Food effect sub-study:
  - On Cycle 1 Day 1: Cmax, Tmax, Tlast, AUClast, AUCtau, AUCinf*, Kel*, t1/2*, CL/F*, Vz/F*
  - On Cycle 1 Day 8 and Cycle 1 Day 15: Cmax, Ctrough, Tmax, Tlast, AUClast, AUCtau, Kel*, t1/2*, CLss/F, Vz/F*
- DDI sub-study:
  - On Cycle 1 Day 1: Cmax, Tmax, Tlast, AUClast, AUCtau, AUCinf*, Kel*, t1/2*, CL/F*, Vz/F*
  - On Cycle 1 Day 8 and Cycle 1 Day 18 (or Cycle 2 Day 10 for subjects who enrolled to sub-study prior to MTD/RP2D determination): Cmax, Ctrough, Tmax, Tlast, AUClast, AUCtau, Kel*, t1/2*, CLss/F, Vz/F*

*: To be calculated only if possible.

**: Accumulation ratio (AR) calculated as AUCtau at steady state /AUCtau following first dose, where tau is the dosing interval of 12 hour

*** Effective half-life can be calculated from AR: ln2 • tau/ ln(AR / (AR-1))

8.4.1. PK Blood Sample Collection and Handling

Time points of PK blood sample collection are outlined in Table 15 (BID dosing), Table 16 (QD dosing), and Table 17 (QD dosing with lead-in period) for Dose Escalation and Table 18 for Dose Expansion in Phase 1. PK sampling associated with intra-patient dose escalation were built into the BID and QD dosing scheme (see Table 15 to Table 17). Time points of PK blood sample collection for the DDI sub-study and food effect sub-study of Dose Escalation are outlined in Table 19 and Table 20 (DDI sub-study) and Table 21 (food effects sub-study).

On PK sampling days, DS-1594b will be administered during the clinic visit as instructed by study personnel. Complete dosing and sampling information, including the exact dates and times of PK blood draw and exact dates and times of the last study drug dose prior to PK sampling and the study drug dose on the day of PK sampling (i.e., dose after pre-dose PK sampling), will be obtained on all PK sampling days and recorded on eCRF and on the CRO requisition form(s). If vomiting occurs within 4 hours of dose administration, the vomiting time should be documented on the eCRF. No re-dosing of the subject will be permitted during that period.

An unscheduled PK sample should be collected if a subject experiences an adverse event that results in an unscheduled visit or fits the criteria of a SAE, or if the triplicate ECG reading shows QTcF interval prolongation of > 60 msec change from baseline or new absolute QTcF ≥ 500 msec.

Residual PK samples may also be used for exploratory analysis to further characterize the PK of DS-1594b and all of its metabolite(s). This may include using leftover samples for protein binding analysis or metabolite profiling (e.g., other metabolites and markers for metabolic enzyme activity such as 4-beta hydroxy cholesterol levels), if there is sufficient sample remaining.

For the food effect sub-study, meal records will be collected including the start and end time of the meal associated with each DS-1594b dose administered in the clinic on PK sampling days (Cycle 1 Day 1 and Cycle 1 Day 8). Compliance with prandial conditions for non-PK sampling days (fed or fasting conditions) will be confirmed by the study personnel during study visits.

For the DDI sub-study, the actual dose, starting date/time and stopping date/time for the anti-fungal agents (posaconazole or voriconazole) must be recorded on the eCRF. If vomiting of these agents occurs within 5 hours of dose administration, the vomiting time should be documented on the eCRF.

All blood samples will be taken by either direct venipuncture or venous catheter or indwelling cannula inserted in a forearm vein. Instructions for the handling of blood samples and shipping of plasma PK samples are included in a separate document (e.g., laboratory manual).

Table 15: DS-1594b PK Blood Collection for Twice a Day (BID) Dosing During Phase 1 [Excluding Subjects Enrolled in DDI and Food Effect Sub-Studies and NOT Applicable for Cohorts with Lead-in Period] Dose Escalation

Cycle	Day	Scheduled Time Point
1	1	Pre-dose/0h[a]
1	1	Post-dose 0.5h (± 5 min)
1	1	Post-dose 1h (± 10 min)
1	1	Post-dose 2h (± 10 min)
1	1	Post-dose 3h (± 10 min)
1	1	Post-dose 4h (± 10 min)
1	1	Post-dose 6h (± 10 min)
1	1	Post-dose 8h (± 10 min)
1	1	Post-dose 12h (± 60 min) (i.e., pre-dose of Cycle 1 Day 1 evening dose)
1	2	Pre-dose/0h[a]
1	5	Pre-dose/0h[a]
1	8	Pre-dose/0h[a]
1	8	Post-dose 0.5h (± 5 min)
1	8	Post-dose 1h (± 10 min)
1	8	Post-dose 2h (± 10 min)
1	8	Post-dose 3h (± 10 min)
1	8	Post-dose 4h (± 10 min)
1	8	Post-dose 6h (± 10 min)
1	8	Post-dose 8h (± 10 min)
1	8	Post-dose 12h (± 60 min) (i.e., pre-dose of Cycle 1 Day 8 evening dose)
1	15	Pre-dose/0h[a]
1	15	Post-dose 2h (± 10 min)
1	15	Post-dose 4h (± 10 min)
1	15	Post-dose 6h (± 10 min)
1	15	Post-dose 8h (± 10 min)
1	21	Pre-dose/0h[a]
1	21	Between 2-4 hours post-dose

Cycle	Day	Scheduled Time Point
2	1	Pre-dose/0h[a]
3	1	Pre-dose/0h[a]
4	1	Pre-dose/0h[a]
5	1	Pre-dose/0h[a]
6	1	Pre-dose/0h[a]
7	1	Pre-dose/0h[a]
8	1	Pre-dose/0h[a]
Unscheduled		Anytime
**After intra-patient dose escalation (see Section 6.9):**		
Cycle X[b] of the new higher dose level or different dosing regimen (frequency)	8	Pre-dose/0h[a]
Cycle X[b] of the new higher dose level or different dosing regimen (frequency)	8	Between 2-4 hours post-dose
Unscheduled		Anytime

All measurement times are relative to dose of DS-1594b unless otherwise specified
[a] PK sample should be taken immediately prior to the next administration of DS-1594b
[b] Cycle X will be the Cycle in which the new dose level or regimen (frequency) is tested as well as any subsequent cycles.

Table 16: DS-1594b PK Blood Collection for Daily (QD) Dosing During Phase 1 [Excluding Subjects Enrolled in DDI and Food Effect Sub-Studies and NOT Applicable for Cohorts with Lead-in period] Dose Escalation

Cycle	Day	Scheduled Time Point
1	1	Pre-dose/0h[a]
1	1	Post-dose 0.5h (± 5 min)
1	1	Post-dose 1h (± 10 min)
1	1	Post-dose 2h (± 10 min)
1	1	Post-dose 3h (± 10 min)
1	1	Post-dose 4h (± 10 min)
1	1	Post-dose 6h (± 10 min)
1	1	Post-dose 8h (± 60 min)
1	2	Pre-dose/0h[b]
1	5	Pre-dose/0h[a]
1	8	Pre-dose/0h[a]
1	8	Post-dose 0.5h (± 5 min)

Cycle	Day	Scheduled Time Point
1	8	Post-dose 1h (± 10 min)
1	8	Post-dose 2h (± 10 min)
1	8	Post-dose 3h (± 10 min)
1	8	Post-dose 4h (± 10 min)
1	8	Post-dose 6h (± 10 min)
1	8	Post-dose 8h (± 60 min)
1	9	Pre-dose/0h[c]
1	15	Pre-dose/0h[a]
1	15	Post-dose 2h (± 10 min)
1	15	Post-dose 4h (± 10 min)
1	15	Post-dose 6h (± 10 min)
1	15	Post-dose 8h (± 60 min)
1	16	Pre-dose/0h[d]
1	21	Pre-dose/0h[a]
1	21	Between 2-4 hours post-dose
2	1	Pre-dose/0h[a]
3	1	Pre-dose/0h[a]
4	1	Pre-dose/0h[a]
5	1	Pre-dose/0h[a]
6	1	Pre-dose/0h[a]
7	1	Pre-dose/0h[a]
8	1	Pre-dose/0h[a]
Unscheduled		Anytime
**After intra-patient dose escalation (see Section 6.9):**		
Cycle X[e] of the new higher dose level or different dosing regimen (frequency)	8	Pre-dose/0h[a]
Cycle X[e] of the new higher dose level or different dosing regimen (frequency)	8	Between 2-4 hours post-dose
Unscheduled		Anytime

All measurement times are relative to dose of DS-1594b unless otherwise specified.

[a] PK sample should be taken immediately prior to the next administration of DS-1594b.

[b] Predose C1D2 PK sample is the same as 24 hours (± 2h) C1D1 post-dose PK sample; it will be collected immediately prior to the administration of DS-1594b.

[c] Predose C1D9 PK sample is the same as 24 hours (± 2h) C1D8 post-dose PK sample; it will be collected immediately prior to the administration of DS-1594b.

[d] Predose C1D16 PK sample is the same as 24 hours (± 2h) C1D15 post-dose PK sample; it will be collected immediately prior to the administration of DS-1594b.

[e] Cycle X will be the Cycle in which the new dose level or regimen (frequency) is tested as well as any subsequent cycles.

Table 17: DS-1594b PK Blood Collection for Daily (QD) Dosing with Lead-in Period (1-Week Interval per Each Step-up Dose Level) During Phase 1 [Excluding Subjects Enrolled in DDI and Food Effect Sub-Studies] Dose Escalation

Treatment Period	Week	Day	Scheduled Time Point	Comment
Lead-in	1	1	Pre-dose/0h[a]	For cohorts with Step-up Dose 1 on Week 1 (see Section 3.1.1)<br><br>e.g., Subjects in cohort(s) with only one Step-up Dose during Lead-in Period (e.g., Cohort 4 in Table 4) will have PK sampling under this Week 1 Schedule, then proceed to PK sampling under Treatment Period.
Lead-in	1	1	Post-dose 1h (± 10 min)	
Lead-in	1	1	Post-dose 2h (± 10 min)	
Lead-in	1	1	Post-dose 3h (± 10 min)	
Lead-in	1	1	Post-dose 4h (± 10 min)	
Lead-in	1	1	Post-dose 6h (± 10 min)	
Lead-in	1	1	Post-dose 8h (± 60 min)	
Lead-in	1	2	Pre-dose/0h[b]	
Lead-in	1	5	Pre-dose/0h[a]	
Lead-in	2	1	Pre-dose/0h[a]	For cohorts with Step-up Dose 2 on Week 2 (see Section 3.1.1)<br><br>e.g., Subjects in cohort(s) with only two Step-up Dose during Lead-in Period (e.g. Cohort 5 in Table 4) will have PK sampling under Week 1 and Week 2 Schedule, then proceed to PK sampling under Treatment Period.
Lead-in	2	1	Post-dose 2h (± 10 min)	
Lead-in	2	1	Post-dose 4h (± 10 min)	
Lead-in	2	1	Post-dose 6h (± 10 min)	
Lead-in	3	1	Pre-dose/0h[a]	For cohorts with Step-up Dose 3 on Week 3 (see Section 3.1.1)<br><br>e.g., Subjects in cohort(s) with three Step-up Dose during Lead-in Period (e.g., Cohort 6 in Table 4) will have PK sampling under Week 1, Week 2 and Week 3 Schedule, then proceed to PK sampling under Treatment Period.
Lead-in	3	1	Post-dose 2h (± 10 min)	
Lead-in	3	1	Post-dose 4h (± 10 min)	
Lead-in	3	1	Post-dose 6h (± 10 min)	
Lead-in	4	1	Pre-dose/0h[a]	For cohorts with Step-up Dose 4 on Week 4 (see Section 3.1.1)
Lead-in	4	1	Post-dose 2h (± 10 min)	
Lead-in	4	1	Post-dose 4h (± 10 min)	

Lead-in	4	1	Post-dose 6h (± 10 min)	e.g., Subjects in cohort(s) with four Step-up Dose during Lead-in Period (e.g., Cohorts 7-9 in Table 4) will have PK sampling under Week 1 to Week 4 Schedule, then proceed to PK sampling under Treatment Period.
Lead-in	Unscheduled		Anytime	
**Treatment Period**	**Cycle**	**Day**	**Scheduled Time Point**	**Comment**
With Target Dose	1	1	Pre-dose/0h[a]	
With Target Dose	1	1	Post-dose 0.5h (± 5 min)	
With Target Dose	1	1	Post-dose 1h (± 10 min)	
With Target Dose	1	1	Post-dose 2h (± 10 min)	
With Target Dose	1	1	Post-dose 3h (± 10 min)	
With Target Dose	1	1	Post-dose 4h (± 10 min)	
With Target Dose	1	1	Post-dose 6h (± 10 min)	
With Target Dose	1	1	Post-dose 8h (± 60 min)	
With Target Dose	1	2	Pre-dose/0h[c]	
With Target Dose	1	5	Pre-dose/0h[a]	
With Target Dose	1	8	Pre-dose/0h[a]	
With Target Dose	1	8	Post-dose 2h (± 10 min)	
With Target Dose	1	8	Post-dose 4h (± 10 min)	
With Target Dose	1	8	Post-dose 6h (± 10 min)	
With Target Dose	1	8	Post-dose 8h (± 60 min)	
With Target Dose	1	9	Pre-dose/0h[d]	
With Target Dose	1	15	Pre-dose/0h[a]	
With Target Dose	1	15	Post-dose 2h (± 10 min)	
With Target Dose	1	15	Post-dose 4h (± 10 min)	
With Target Dose	1	15	Post-dose 6h (± 10 min)	
With Target Dose	1	15	Post-dose 8h (± 60 min)	
With Target Dose	1	16	Pre-dose/0h[e]	
With Target Dose	1	21	Pre-dose/0h[a]	
With Target Dose	1	21	Between 2-4 hours post-dose	
With Target Dose	2	1	Pre-dose/0h[a]	
With Target Dose	3	1	Pre-dose/0h[a]	
With Target Dose	4	1	Pre-dose/0h[a]	
With Target Dose	5	1	Pre-dose/0h[a]	
With Target Dose	6	1	Pre-dose/0h[a]	

With Target Dose	7	1	Pre-dose/0h[a]	
With Target Dose	8	1	Pre-dose/0h[a]	
With Target Dose	Unscheduled		Anytime	
With Target Dose	Cycle X[f] of the new higher dose level or different dosing regimen (frequency)	8	Pre-dose/0h[a]	**After intra-patient dose escalation (see Section 6.9)**
	Cycle X[f] of the new higher dose level or different dosing regimen (frequency)	8	Between 2-4 hours post-dose	

All measurement times are relative to dose of DS-1594b unless otherwise specified.
[a] PK sample should be taken immediately prior to the next administration of DS-1594b.
[b] Predose PK Sample on Week 1 Day 2 of Lead-in Period is the same as 24 hours (± 2h) post-dose Week1 Day 1 PK sample; it will be collected immediately prior the administration of DS-1594b.
[c] Predose C1D2 PK sample is the same as 24 hours (± 2h) C1D1 post-dose PK sample; it will be collected immediately prior to the administration of DS-1594b.
[d] Predose C1D9 PK sample is the same as 24 hours (± 2h) C1D8 post-dose PK sample; it will be collected immediately prior to the administration of DS-1594b.
[e] Predose C1D16 PK sample is the same as 24 hours (± 2h) C1D15 post-dose PK sample; it will be collected immediately prior to the administration of DS-1594b.
[f] Cycle X will be the Cycle in which the new dose level or regimen (frequency) is tested as well as any subsequent cycles.

Table 18: DS-1594b PK Blood Collection During Phase 2 Dose Expansion

Cycle	Day	Scheduled Time Point	Comment
1	1	Pre-dose/0h[a]	
1	1	Post-dose 0.5h (± 5 min)	Optional pending Phase 1data
1	1	Post-dose 1h (± 10 min)	
1	1	Post-dose 2h (± 10 min)	
1	1	Post-dose 4h (± 10 min)	
1	1	Post-dose 6h (± 10 min)	
1	1	Post-dose 8h (± 60 min)	
1	2	Pre-dose/0h[b]	For daily (QD) dosing only
1	8	Pre-dose/0h[a]	
1	8	Post-dose 0.5h (± 5 min)	Optional pending Phase 1 data
1	8	Post-dose 1h (± 10 min)	
1	8	Post-dose 2h (± 10 min)	
1	8	Post-dose 4h (± 10 min)	
1	8	Post-dose 6h (± 10 min)	
1	8	Post-dose 8h (± 60 min)	

Cycle	Day	Scheduled Time Point	Comment
1	9	Pre-dose/0h[c]	For daily (QD) dosing only
1	15	Pre-dose/0h[a]	
1	15	Between 2-4 hours post-dose	
2	1	Pre-dose/0h[a]	
3	1	Pre-dose/0h[a]	
4	1	Pre-dose/0h[a]	
5	1	Pre-dose/0h[a]	
6	1	Pre-dose/0h[a]	
Unscheduled		Anytime	

All measurement times are relative to dose of DS-1594b unless otherwise specified
[a] PK sample should be taken immediately prior to the next administration of DS-1594b
[b] Predose C1D2 PK sample is the same as 24 hours (± 2h) C1D1 post-dose PK sample; it will be collected immediately prior the administration of DS-1594b
[c] Predose C1D9 PK sample is the same as 24 hours (± 2h) C1D8 post-dose PK sample; it will be collected immediately prior the administration of DS-1594b

Table 19: DS-1594b PK Blood Collection for DDI Sub-Study with Antifungal Agents During Phase 1 After MTD Determination (N ~ 12)

Cycle	Day	Scheduled Time Point	Comment
1	1	Pre-dose/0h[a]	
1	1	Post-dose 1h (± 10 min)	
1	1	Post-dose 2h (± 10 min)	
1	1	Post-dose 4h (± 10 min)	
1	1	Post-dose 6h (± 10 min)	
1	1	Post-dose 8h (± 10 min)	
1	8	Pre-dose/0h[a]	Full PK profile in the absence of antifungal agents
1	8	Post-dose 0.5h (± 5 min)	
1	8	Post-dose 1h (± 10 min)	
1	8	Post-dose 2h (± 10 min)	
1	8	Post-dose 3h (± 10 min)	
1	8	Post-dose 4h (± 10 min)	
1	8	Post-dose 6h (± 10 min)	
1	8	Post-dose 8h (± 10 min)	
1	8	Post-dose 12h (± 60 min) (i.e., pre-dose of Cycle 1 Day 8 evening dose) [for BID dosing only]	
1	9	Pre-dose/0h[b] [for daily dosing only]	
1	18	Pre-dose/0h[a]	

Cycle	Day	Scheduled Time Point	Comment
1	18	Post-dose 0.5h (± 5 min)	Full PK profile in the presence of antifungal agents
1	18	Post-dose 1h (± 10 min)	
1	18	Post-dose 2h (± 10 min)	
1	18	Post-dose 3h (± 10 min)	
1	18	Post-dose 4h (± 10 min)	
1	18	Post-dose 6h (± 10 min)	
1	18	Post-dose 8h (± 10 min)	
1	18	Post-dose 12h (± 60 min) (i.e., pre-dose of Cycle 1 Day 18 evening dose) [for BID dosing only]	
1	19	Pre-dose/0h[c] [for daily dosing only]	
2	1	Pre-dose/0h[a]	
3	1	Pre-dose/0h[a]	
4	1	Pre-dose/0h[a]	
Unscheduled		Anytime	

All measurement times are relative to dose of DS-1594b unless otherwise specified
[a] PK sample should be taken immediately prior to the next administration of DS-1594b
[b] Predose C1D9 PK sample is the same as 24 hours (± 2h) C1D8 post-dose PK sample; it will be collected immediately prior the administration of DS-1594b
[c] Predose C1D19 PK sample is the same as 24 hours (± 2h) C1D18 post-dose PK sample; it will be collected immediately prior the administration of DS-1594b

Table 20: DS-1594b PK Blood Collection for DDI Sub-Study with Antifungal Agents During Phase 1 Before MTD Determination

Cycle	Day	Scheduled Time Point	Comment
1	1	Pre-dose/0h[a]	
1	1	Post-dose 0.5h (± 5 min)	
1	1	Post-dose 1h (± 10 min)	
1	1	Post-dose 2h (± 10 min)	
1	1	Post-dose 3h (± 10 min)	
1	1	Post-dose 4h (± 10 min)	
1	1	Post-dose 6h (± 10 min)	
1	1	Post-dose 8h (± 10 min)	
1	1	Post-dose 12h (± 60 min) (i.e., pre-dose of Cycle 1 Day 1 evening dose) [for BID dosing only]	
1	2	Pre-dose/0h[a]	
1	5	Pre-dose/0h[a]	
1	8	Pre-dose/0h[a]	

Cycle	Day	Scheduled Time Point	Comment
1	8	Post-dose 0.5h (± 5 min)	Full PK profile in the absence of antifungal agents
1	8	Post-dose 1h (± 10 min)	
1	8	Post-dose 2h (± 10 min)	
1	8	Post-dose 3h (± 10 min)	
1	8	Post-dose 4h (± 10 min)	
1	8	Post-dose 6h (± 10 min)	
1	8	Post-dose 8h (± 10 min)	
1	8	Post-dose 12h (± 60 min) (i.e., pre-dose of Cycle 1 Day 8 evening dose) [for BID dosing only]	
1	9	Pre-dose/0h[b] [for daily dosing only]	
2	10	Pre-dose/0h[a]	Full PK profile in the presence of antifungal agents
2	10	Post-dose 0.5h (± 5 min)	
2	10	Post-dose 1h (± 10 min)	
2	10	Post-dose 2h (± 10 min)	
2	10	Post-dose 3h (± 10 min)	
2	10	Post-dose 4h (± 10 min)	
2	10	Post-dose 6h (± 10 min)	
2	10	Post-dose 8h (± 10 min)	
2	10	Post-dose 12h (± 60 min) (i.e., pre-dose of Cycle 2 Day 10 evening dose) [for BID dosing only]	
2	11	Pre-dose/0h[c] [for daily dosing only]	
3	1	Pre-dose/0h[a]	
4	1	Pre-dose/0h[a]	
5	1	Pre-dose/0h[a]	
6	1	Pre-dose/0h[a]	
Unscheduled		Anytime	

All measurement times are relative to dose of DS-1594b unless otherwise specified

[a] PK sample should be taken immediately prior to the next administration of DS-1594b

[b] Predose C1D9 PK sample is the same as 24 hours (± 2h) C1D8 post-dose PK sample; it will be collected immediately prior the administration of DS-1594b

[c] Predose C2D11 PK sample is the same as 24 hours (± 2h) C2D10 post-dose PK sample; it will be collected immediately prior the administration of DS-1594b

Table 21: DS-1594b PK Blood Collection for Food Effect Sub-Study During Phase 1 After MTD Determination (N ~ 6)

Cycle	Day	Scheduled Time Point	Comment
1	1	Pre-dose/0h[a]	

Cycle	Day	Scheduled Time Point	Comment
1	1	Post-dose 1h (± 10 min)	
1	1	Post-dose 2h (± 10 min)	
1	1	Post-dose 4h (± 10 min)	
1	1	Post-dose 6h (± 10 min)	
1	1	Post-dose 8h (± 10 min)	
1	8	Pre-dose/0h[a]	Full PK profile under fed condition with standard breakfast
1	8	Post-dose 0.5h (± 5 min)	
1	8	Post-dose 1h (± 10 min)	
1	8	Post-dose 2h (± 10 min)	
1	8	Post-dose 3h (± 10 min)	
1	8	Post-dose 4h (± 10 min)	
1	8	Post-dose 6h (± 10 min)	
1	8	Post-dose 8h (± 10 min)	
1	8	Post-dose 12h (± 60 min) (i.e., pre-dose of Cycle 1 Day 8 evening dose) [for BID dosing only]	
1	9	Pre-dose/0h[b] [for daily dosing only]	
1	18	Pre-dose/0h[a]	Full PK profile under fasting condition
1	18	Post-dose 0.5h (± 5 min)	
1	18	Post-dose 1h (± 10 min)	
1	18	Post-dose 2h (± 10 min)	
1	18	Post-dose 3h (± 10 min)	
1	18	Post-dose 4h (± 10 min)	
1	18	Post-dose 6h (± 10 min)	
1	18	Post-dose 8h (± 10 min)	
1	18	Post-dose 12h (± 60 min) (i.e., pre-dose of Cycle 1 Day 18 evening dose) [for BID dosing only]	
1	19	Pre-dose/0h[c] [for daily dosing only]	
2	1	Pre-dose/0h[a]	
3	1	Pre-dose/0h[a]	
4	1	Pre-dose/0h[a]	
Unscheduled		Anytime	

All measurement times are relative to dose of DS-1594b unless otherwise specified

[a] PK sample should be taken immediately prior to the next administration of DS-1594b

[b] Predose C1D9 PK sample is the same as 24 hours (± 2h) C1D8 post-dose PK sample; it will be collected immediately prior the administration of DS-1594b

[c] Predose C1D19 PK sample is the same as 24 hours (± 2h) C1D18 post-dose PK sample; it will be collected immediately prior the administration of DS-1594b

8.4.2. Analytical Methods

Plasma DS-1594b and metabolites (if appropriate) will be measured at a laboratory designated by Daiichi Sankyo using a validated LC/MS assay.

8.5. Cardiac Assessment (Electrocardiograms)

A standard 12-lead ECG or Holter monitor will be used to collect the ECG timepoints described in Table 22 and Table 23 (Phase 1) and Table 24 (Phase 2). At all scheduled timepoints, ECGs must be performed in triplicate. The triplicate ECGs must be taken approximately ≥1 minute apart, with the subject in the supine/semi-recumbent position. All ECGs recorded for each time point must be transmitted electronically to a central laboratory and must be centrally reviewed by an independent reviewer. Any original ECG not transmitted electronically to the central laboratory must be forwarded for central review. **All ECGs must be performed within 15 mins prior to the collection of all PK samples.**

The ECG collection plan is described in Table 22 and Table 23 for Phase 1 Dose Escalation and Table 24 for Phase 1 Dose Expansion / Phase 2.

Table 22: ECG Collection During Phase 1 Escalation (Excluding Cohorts with Lead-in Period)

Cycle	Day	Time	Comment
Screening	-28 to -1	Anytime	
1	1	Pre-dose/0h[a]	See footnote for details of 3 sets of triplicates
1	1	Post-dose 2h	
1	1	Post-dose 4h	
1	1	Post-dose 6h	
1	1	Post-dose 8h	
1	1	Post-dose 12h[c] (or Cycle 1 Day 1 pre-evening dose)	For BID dosing only
1	2	Pre-dose/0h[b] (Post-dose 12h from evening dose on Cycle 1, Day 1 [if BID dosing], or post-dose 24h from morning dose on Cycle 1, Day 1 [if QD dosing])	
1	5	Pre-dose/0h[b]	
1	8	Pre-dose/0h[b]	
1	8	Post-dose 2h	
1	8	Post-dose 4h	
1	8	Post-dose 6h	
1	8	Post-dose 8h	
1	8	Post-dose 12h[c] (or Cycle 1 Day 8 pre-evening dose)	For BID dosing only
1	9	Pre-dose/0h[b] (same as post-dose 24 h of C1D8)	For daily (QD) dosing only
1	15	Pre-dose/0h[b]	
1	15	Post-dose 2h	

1	15	Post-dose 4h	
1	18	Pre-dose/0h[b]	DDI sub-study only[e]
1	18	Post-dose 2h	DDI sub-study only[e]
1	18	Post-dose 4h	DDI sub-study only[e]
1	18	Post-dose 6h	DDI sub-study only[e]
1	18	Post-dose 8h	DDI sub-study only[e]
1	18	Post-dose 12h[c] (or Cycle 1 Day 18 pre-evening dose)	DDI sub-study only[e] For BID dosing only
1	19	Pre-dose/0h[b] (same as post-dose 24 h of C1D18)	DDI sub-study only[e] For QD dosing only
1	21	Pre-dose/0h[b]	
1	21	Between 2-4 hours post-dose	
2	1	Pre-dose/0h[b]	
2	10	Pre-dose/0h[b]	DDI sub-study only[f]
2	10	Post-dose 2h	DDI sub-study only[f]
2	10	Post-dose 4h	DDI sub-study only[f]
2	10	Post-dose 6h	DDI sub-study only[f]
2	10	Post-dose 8h	DDI sub-study only[f]
2	10	Post-dose 12h[c] (or Cycle 2 Day 10 pre-evening dose)	DDI sub-study only[f] For BID dosing only
2	11	Pre-dose/0h[b] (same as post-dose 24 h of C2D10)	DDI sub-study only[f] For QD dosing only
3-8	1	Pre-dose/0h[b]	
During treatment after Cycle 6	Any day[d]	Anytime	
**After intra-patient dose escalation (see Section 6.9):**			
Cycle X of the new higher dose level or different dosing regimen (frequency)	8	Pre-dose/0h[b]	
Cycle X of the new higher dose level or different dosing regimen (frequency)	8	Between 2-4 hours post-dose	
End of Treatment	--	--	

All measurement times are relative to dose of DS-1594b unless otherwise specified

[a] Pre-dose assessment sets on C1D1 should be taken prior to the next administration of DS-1594b at -45 mins (triplicate), -30 mins (triplicate) and -15 mins (triplicate)

[b] Pre-dose assessment should be taken prior to the next administration of DS-1594b and within 15 mins prior to PK collection

[c] 12-hour assessment must be performed prior to the next dose and within 15 mins prior to PK collection

[d] ECG should be performed only if clinically indicated

[e] For subjects participating in the DDI sub-study AFTER MTD declaration (N ~ 12), additional triplicate ECGs will be performed on Cycle 1 Day 18 and Day 19 to coincide with PK assessments in which DS-1594b is co-administered with antifungal agents (posaconazole, voriconazole) that are potent CYP3A inhibitors

[f] For subjects participating in the DDI sub-study BEFORE MTD declaration, additional triplicate ECGs will be performed on Cycle 2 Day 10 and Day 11 to coincide with PK assessments in which DS-1594b is co-administered with antifungal agents (posaconazole) that are potent CYP3A inhibitors

Table 23: ECG Collection During Phase 1 Escalation For Cohorts with Lead-in Period

Treatment Period	Week	Day	Scheduled Time Point	Comment
Lead-in	0	-28 to -1	Anytime	
Lead-in	1	1	Pre-dose/0h[a]	For cohorts with Step-up Dose 1 on Week 1 (see Section 3.1.1)<br><br>e.g., Subjects in cohort(s) with only one Step-up Dose during Lead-in Period (e.g., Cohort 4 in Table 4) will have ECG collection under this Week 1 Schedule, then proceed to collection under Treatment Period.
Lead-in	1	1	Post-dose 2h	
Lead-in	1	1	Post-dose 4h	
Lead-in	1	1	Post-dose 6h	
Lead-in	1	1	Post-dose 8h	
Lead-in	1	2	Pre-dose/0h[b] (Post-dose 24h from morning dose on Week 1, Day 1	
Lead-in	1	5	Pre-dose/0h[b]	
Lead-in	2	1	Pre-dose/0h[b]	For cohorts with Step-up Dose 2 on Week 2 (see Section 3.1.1)<br><br>e.g., Subjects in cohort(s) with only two Step-up Dose during Lead-in Period (e.g., Cohort 5 in Table 4) will have ECG collection under Week 1 and Week 2 Schedule, then proceed to collection under Treatment Period.
Lead-in	2	1	Post-dose 2h	
Lead-in	2	1	Post-dose 4h	
Lead-in	2	1	Post-dose 6h	
Lead-in	3	1	Pre-dose/0h[b]	For cohorts with Step-up Dose 3 on Week 3 (see Section 3.1.1)<br><br>e.g., Subjects in cohort(s) with three Step-up Dose during Lead-in Period (e.g., Cohort 6 in Table 4) will have ECG
Lead-in	3	1	Post-dose 2h	
Lead-in	3	1	Post-dose 4h	
Lead-in	3	1	Post-dose 6h	

				collection under Week 1, Week 2 and Week 3 Schedule, then proceed to collection under Treatment Period.
Lead-in	4	1	Pre-dose/0h[b]	For cohorts with Step-up Dose 4 on Week 4 (see Section 3.1.1)<br><br>e.g., Subjects in cohort(s) with four Step-up Dose during Lead-in Period (e.g., Cohorts 7-9 in Table 4) will have ECG collection under Week 1 to Week 4 Schedule, then proceed to collection under Treatment Period.
Lead-in	4	1	Post-dose 2h	
Lead-in	4	1	Post-dose 4h	
Lead-in	4	1	Post-dose 6h	
**Treatment Period**	**Cycle**	**Day**	**Scheduled Time Point**	**Comment**
With Target Dose	1	1	Pre-dose/0h[b]	
With Target Dose	1	1	Post-dose 2h	
With Target Dose	1	1	Post-dose 4h	
With Target Dose	1	1	Post-dose 6h	
With Target Dose	1	1	Post-dose 8h	
With Target Dose	1	2	Pre-dose/0h[b]	
With Target Dose	1	8	Pre-dose/0h[b]	
With Target Dose	1	8	Post-dose 2h	
With Target Dose	1	8	Post-dose 4h	
With Target Dose	1	8	Post-dose 6h	
With Target Dose	1	8	Post-dose 8h	
With Target Dose	1	15	Pre-dose/0h[b]	
With Target Dose	1	15	Post-dose 2h	
With Target Dose	1	15	Post-dose 4h	
With Target Dose	1	21	Pre-dose/0h[b]	
With Target Dose	1	21	Between 2-4 hours post-dose	
With Target Dose	2-8	1	Pre-dose/0h[b]	
With Target Dose	During treatment after Cycle 6	Any day[c]	Anytime	

With Target Dose	Cycle X[d] of the new higher dose level or different dosing regimen (frequency)	8	Pre-dose/0h[b]	**After intra-patient dose escalation (see Section 6.9)**
	Cycle X[d] of the new higher dose level or different dosing regimen (frequency)	8	Between 2-4 hours post-dose	

All measurement times are relative to dose of DS-1594b unless otherwise specified
[a] Pre-dose assessment sets on Week 1 Day 1 of Lead-in Period should be taken prior to the next administration of DS-1594b at -45 mins (triplicate), -30 mins (triplicate) and -15 mins (triplicate)
[b] Pre-dose assessment should be taken prior to the next administration of DS-1594b and within 15 mins prior to PK collection
[c] ECG should be performed only if clinically indicated
[d] Cycle X will be the Cycle in which the new dose level or regimen (frequency) is tested as well as any subsequent cycles.

Table 24: ECG Collection During Phase 2 Dose Expansion

Cycle	Day	Time	Comment
Screening	-28 to -1	Anytime	
1	1	Pre-dose/0h[a]	
1	1	Post-dose 2h	
1	1	Post-dose 4h	
1	8	Pre-dose/0h[a]	
1	8	Post-dose 2h	
1	8	Post-dose 4h	
1	15	Pre-dose/0h[a]	
1	15	Between 2-4 hours post-dose	
2-6	1	Pre-dose/0h[a]	

Cycle	Day	Time	Comment
During treatment after Cycle 6	Any day[b]	Anytime	
End of Treatment	--	--	

All measurement times are relative to dose of DS-1594b unless otherwise specified.
[a] Pre-dose assessment should be taken prior to the next administration of DS-1594b and within 15 minutes prior to PK collection.
[b] ECG should be performed only if clinically indicated.

8.6. Correlative Studies (Mandatory for Phase 1)

Tumor tissue, blood samples and bone marrow aspirate/biopsy for correlative research will be collected. Correlative laboratory studies will be conducted under this clinical trial as described:

- **Peripheral blood** up to 40 mL (within 24 hours) will be collected for testing of biomarkers at Baseline (prior to day 1 dose of drug), Day 1 (±2) of each lead-in dosing level during the lead-in period (if applicable), on Cycle 1 Days 8 (± 2), 15 (± 2), 21(± 2), 28(± 2), on Cycle 2, Day 15(± 2), on Cycles 3 and 4, Day 1(± 2) and at end of treatment / progression (if possible). Additional timepoints may be collected as needed.
- **Bone marrow** samples will be collected for testing of biomarkers at baseline, at the end of the lead-in period (± 4 days) (optional and only in subjects receiving lead-in dosing ≥ 21 days), on Day 28 (± 4 days), at Day 28 (± 4 days) of Cycles 2, 3, 6, 9, and at end of treatment or progression (if possible). Additional timepoints may be collected as needed.
- All subjects are also routinely monitored for MRD by multiparametric flow cytometry and RT-PCR or NGS (in some cases, when applicable) at the time of bone marrow assessments. This will allow an analysis of response beyond hematologic criteria and correlation with long-term outcome.
- All subjects will be evaluated by a pre-therapy and on-treatment NGS molecular panels, FISH, routine karyotyping, immunophenotyping and by a leukemia fusion panel (as applicable), to investigate correlations of gene alterations with long-term outcome, and to identify molecular characteristics of residual or recurrent clones.
- Identification of clonal subsets in AML has almost entirely been done using genomics, in which cellular populations are inferred. In order to effectively characterize and ultimately treat these cells, we need tools to identify therapy resistant cell populations as they arise in subjects. Flow cytometry is a useful tool for tracking cells in subjects but traditional fluorescent cytometry is limited by panel size and scope. Cytometry by Time of Flight (CyTOF), is a variation of flow cytometry in which antibodies are labeled with heavy metal ion tags rather than fluorochromes. This allows for the combination of many more antibody specificities in a single cell with greater depth and breadth of phenotypic and functional cytometric profiling. CyTOF will enable us to identify individual treatment-resistant cell populations and

their signaling state in disease related to clinical outcomes. CyTOF will be performed on bone marrow samples and peripheral blood at screening, remission, longitudinal samples on study, and relapse (where possible).

- To enable the characterization of genetic heterogeneity in tumor cell populations, we will use a novel microfluidic approach that barcodes amplified genomic DNA from thousands of individual leukemia cells confined to droplets. The barcodes are then used to reassemble the genetic profiles of cells from next-generation sequencing data. By using this approach, we will sequence longitudinally collected AML tumor populations from subjects and genotype disease relevant loci across thousands of individual cells. Targeted single-cell sequencing is able to sensitively identify cells harboring pathogenic mutations during complete remission and uncover complex clonal evolution within AML tumors that are not observable with bulk sequencing. We anticipate that this approach will make feasible the routine analysis of AML heterogeneity, leading to improved stratification and therapy selection for the subjects. Single cell sequencing will be performed on bone marrow samples at screening, remission, longitudinal samples on study, and relapse (where possible).
- Additional analysis including but not limited to bulk RNA sequencing to include Menin dependent genes (MEIS1, PBX3, MEF2C, and others), single cell RNA sequencing, IHC for differentiation markers on bone marrow slides/blocks, plasma inhibitory activity (PIA), RT-MLP, and genomic break point sequencing may be performed on the subjects' bone marrow samples and peripheral blood at screening, remission, longitudinal samples on study, and relapse (where possible).

Specific correlative science studies to evaluate the effects of single-agent DS-1594 in patients with R/R AML in Cycle 1 (Phase I and II, cohorts A and B only):

AML is a hematologic malignancy of the bone marrow that commonly also presents with circulating AML blasts. Therefore, there is the unique opportunity to examine predictive genetic and gene-expression biomarkers of response and pharmacodynamic effects in vivo and ex vivo (in vitro) of the Menin Inhibitor (MI) DS1594b on the BMA and/or peripheral blood (PB)-derived AML blasts of the patients. Correlations of the DS1594b mRNA and protein signature in vivo in patients' AML cells will be made with response or resistance to DS1594b, which will also be correlated with the genetic mutation status of AML cells in patients treated on the **Phase II, Cohort A and B of this trial.** Such testing would be minimally invasive. PB drawn into green-top heparinized tubes will be utilized for evaluating genetic alterations or gene expression profile on AML cells. Such tests will also be done on BMA samples pre-treatment and when they are performed/available for assessment of response. The planned sample collection for these studies is outlined in the Table below. Every effort will be made to ensure that blood draws for correlative studies coincide with each other and with routine bloodwork. For PB a maximum of up to 40 mL (within 24 hours) can be collected for testing of all

biomarkers and will be observed. The correlative studies listed in this section are optional in that failure to collect the samples will not constitute a protocol deviation/violation.

As part of the standard workup in the leukemia department at MD Anderson, patients bone marrow aspirate (BMA) sample will undergo cytogenetic and NGS (NextGen Sequencing) evaluation on a customized 81 gene NGS panel of common leukemia related mutations. Additionally, BMA sample will also be evaluated by FISH and/or Array-CGH to detect MLL1r. these studies will be supervised in Hematopathology Department by Dr. Joseph Khoury (multiple-PI on this trial).

Because of the nature and potential role of the clonal heterogeneity of AML and its impact on treatment resistance and clinical outcome, in addition to NGS, we will determine, via high-throughput scDNA-Seq platform (Tapestri), supervised by Dr. Koichi Takahashi, the clonal architecture of AML with MLL1r or NPM1c+ (1-3). We have reported that scDNA-Seq allows precise characterization of clonal heterogeneity by rapid targeted genotyping of the AML-driver genes in approximately 7500 cells/sample with low allelic dropout (39,40). This illuminates the clonal architecture, including linear (in 55%) or branching model (45%) of clonal evolution of AML. These studies will also simultaneously involve immunophenotype analysis (also called DAB-Seq), determining cell surface protein expression of CD34, CD11b, CD38, CD123, CD90 and CD45RA (40,41).

BMA sample and/or PB -derived AML cells that de-identified and anonymized will be purified, and attempt will be made to expand the number of AML cells by taking a portion of the cells and injecting and engrafting them into immune depleted mice, where they grow in the bone marrow, spleen and blood of the mice (42-44). This will establish a patient derived xenograft (PDX), enabling future expansion of AML cells for in vitro and in vivo testing of novel anti-AML drugs and/or their combinations for their efficacy in immune depleted mice. For these studies, AML cells with MLL1r or NPM1c+ will be harvested from anonymized BMA and/or PB samples from patients who are enrolled on either the Phase 1 or Phase II (Cohort A and B) of the clinical trial. These studies will be conducted in the laboratory of Dr. Kapil Bhalla (multiple-PI).

Pre-treatment and/or on-treatment (on DS1594b) BMA and PB-derived AML cells will also be utilized for the following set of in vitro studies, methodology for these have been reported previously by us (42-44):

- ✓ Bulk ATAC-Sec, or single cell ATAC-Seq on BMA sample
- ✓ Bulk RNA-Seq, or single cell RNA-Seq on BMA sample
- ✓ RPPA
- ✓ CyTOF
- ✓ Confocal Microscopy

Correlative science studies to determine in vivo effects of DS1594b in BMA or PB-derived AML cells during Cycle 1 (Phase I and Phase II, Cohorts A and B)

	Day 0 (prior to starting treatment with DS1594b)	Day 5 (Four hours post morning dose)	Day 15* (or Day 21) * (only if AML is unresponsive or progressing)
Bone marrow aspirate (BMA) Sample for<br><br>NGS, sc-DNA-Seq, FCM, and epigenome/ transcriptome/proteome analysis via scATAC-Seq, scRNA-Seq, qPCR, CyTOF and RPPA	X		X
Peripheral blood (PB) (two 10 ml heparinized tubes) for CD34+ or mononuclear cells	X	X	X
ATAC-Seq analyses on nuclei to determine accessible chromatin profile	X	X	X
RNA-Seq and qPCR analyses on total RNA to determine mRNA expression for gene-sets and mRNA signature profile	X	X	X
RPPA on cell lysates to determine protein expression profile	X	X	X
Flow Cytometry/CyTOF on paraformaldehyde fixed cells	X	X	X
Confocal microscopy on cytospun paraformaldehyde-fixed cells	X	X	X

8.7. Outside Physician Participation During Treatment

- The first 28 days of DS-1594b must be administered at the site, either in-patient or out-patient. It is recommended (but not required) that subjects be in-patient for the initial 7-10 days of DS-1594b administration to monitor for signs and symptoms of differentiation syndrome. The first 7 days of azacitidine and first cycle of mini-HCVD infusions must be administered at the site. Subsequently, subjects will have the option of receiving azacitidine injections or mini-HCVD infusions at the site out-patient clinic or the local ambulatory treatment center/outside physician's office. We do not intend for the subjects to receive DS-1594b at any time at an outside physician's office. During the first 28 days of dosing in both Dose Escalation and Dose Expansion of Phase 1 all the laboratory evaluations will be done at the site and the subjects must stay locally within easy access of the site. Subsequently, the subject may have the laboratory work done at a local clinic and the results reported and filed by the site research nurse for the study. The laboratory work done at the local clinic will be forwarded to the investigator or PI of the study, who will sign off on the labs to verify that the results have been reviewed.

- Investigator communication with the outside physician is required prior to the subject returning to the local physician.
- This will be documented in the subject record.
- A letter to the local physician outlining the subject's participation in a clinical trial will request local physician agreement to supervise the subject's care.
- Protocol required evaluations outside of the site will be documented by telephone, fax or e-mail. Fax and/or e-mail will be dated and signed by the Investigator, indicating that they have reviewed it. Changes in drug dose and/or schedule must be discussed with and approved by the Investigator, or they're representative prior to initiation, and will be documented in the subject record.
- A copy of the informed consent, and treatment schema and evaluation during treatment will be provided to the local physician.
- Documentation to be provided by the local physician will include progress notes, reports of protocol required laboratory and diagnostic studies and documentation of any hospitalizations.
- The home physician will be requested to report to the Investigator all life-threatening events within 24 hours of documented occurrence.
- All protocol required follow-up visits will be performed at the site.
- Changes in drug dose and/or schedule must be discussed with and approved by the investigator, or they're representative prior to initiation, and will be documented in the subject record.
- Subjects with an objective response at completion of active study treatment will be followed for survival at the site every 3 to 6 months for up to 5 years after completion of active treatment and while still on study. If the subject is unable to return to the site, the duration visits may be conducted via telephone.
- Data regarding adverse events will be collected during the study. Protocol specific data will be entered into the electronic case report form (eCRF). The eCRF used for this protocol will be Prometheus. AEs will be recorded in the Case Report Form.
- Treatment may be discontinued for a variety of reasons, including subject withdrawal, investigator decision, and reasons specified by the protocol. Reasons for discontinuation of treatments are described in Section 8.9.1.

8.8. CRITERIA FOR RESPONSE:

8.8.1. Response Criteria for AML

Responses will be assessed by the criteria below, adapted from the International Working Group for AML and the European Leukemia Net Guidelines[23,24]. Responders are subjects who obtain a CR, CRh, CRi, or PR, with or without cytogenetic response, or a morphologic leukemia-free state.

Complete remission (CR)

- Peripheral blood counts: No circulating blasts.
- Neutrophil count: ≥1.0 x 10^9/L
- Platelet count ≥100 x 10^9/L.
- Bone marrow aspirate and biopsy: < 5% blasts.
- No Auer rods.
- No extramedullary leukemia

Complete remission with incomplete blood count recovery (CRi)

- Peripheral blood counts: No circulating blasts
- Neutrophil count: <1.0 x 10^9/L, OR
- Platelet count: <100 x 10^9/L
- Bone marrow aspirate and biopsy: <5% blasts
- No Auer rods
- No extramedullary leukemia

Complete Remission with Partial Hematological Recovery (CRh)

- Peripheral blood counts: No circulating blasts
- Neutrophil count: ≥ 0.5 x 10^9/L
- Platelet count: ≥ 50 x 10^9/L
- Bone marrow aspirate and biopsy: < 5% blasts
- No Auer rods
- No extramedullary leukemia

Partial remission (PR)

- Neutrophil count: ≥1.0 x 10^9/L
- Platelet count ≥100 x 10^9/L
- ≥ 50 % reduction in bone marrow blast from pretreatment baseline, but still ≥ 5%

Morphologic leukemia-free state (MLFS):

- Bone marrow < 5% myeloblasts,
- No Auer rods,
- No extramedullary leukemia,
- Neutrophil or platelet recovery is not required

8.8.2. Response Criteria for ALL

Complete Remission (CR)

- No circulating lymphoblasts or extramedullary disease
  - No lymphadenopathy, splenomegaly, skin/gum infiltration/testicular mass/CNS involvement
- Trilineage hematopoiesis (TLH) and <5% blasts
- Neutrophil count: $\geq$1.0 x 10^9/L
- Platelet count $\geq$100 x 10^9/L
- No recurrence for 4 weeks

Complete Remission with Partial Hematological Recovery (CRh)

- No circulating lymphoblasts or extramedullary disease
  - No lymphadenopathy, splenomegaly, skin/gum infiltration/testicular mass/CNS involvement
- Neutrophil count: $\geq$ 0.5 x 10^9/L
- Platelet count: $\geq$ 50 x 10^9/L
- Trilineage hematopoiesis (TLH) and <5% blasts
- No recurrence for 4 weeks

Complete Remission with Incomplete Hematologic Recovery (CRi)

- Meets all criteria for CR except platelet count or ANC

8.8.3. Transfusion Independence (TI)

TI is defined as the absence of red blood cell and platelet transfusions for a consecutive 56-day period during continued treatment.

TI will be assessed as a response achieved in the subgroup of subjects who were transfusion dependent (TD) at baseline (conversion from TD to TI with treatment) separately from the subgroup of subjects who were TI at baseline (maintenance of TI with treatment). Transfusion dependence at baseline is based on the receipt of any red blood cell or platelet transfusions within at least 28 days prior to the start of study treatment. Analyses of red blood cell TI and platelet TI will also be assessed separately.

8.8.4. Relapse Definition (AML and ALL)

Hematological relapse is defined as marrow blasts $\geq$ 5% by morphology, persistent reappearance of blasts in the peripheral blood by morphology, or the occurrence of extramedullary disease after achieving remission (CR, CRh, CRi, or MLFS).

When relapse is suspected on the basis of the follow-up physical examination or peripheral blood counts, additional testing may be performed to confirm the finding, but the date of relapse is set to the date of the first test that suggests relapse.

8.9. DISCONTINUATION OF TREATMENT

8.9.1. Discontinuation Criteria for Individual Subjects

Subject Withdrawal

Subjects may voluntarily withdraw consent to participate in the clinical study at any time and without giving any reason. For subjects who withdraw consent, the Investigator may search publicly available records (where permitted) to ascertain survival status. Their withdrawal will not jeopardize their relationship with their healthcare providers or affect their future care. Subjects may also choose to withdraw from study treatment but agree to remain in the study for follow-up procedures.

Investigator Discontinuation of Subject

The Investigator may exercise medical judgment to discontinue study treatment if clinically significant changes in clinical status or laboratory values are noted.

Criteria for Protocol-Defined Required Discontinuation of Treatment

The protocol requires discontinuation of study treatment for the following reasons:

- Lack of clinical benefit (defined as failure to achieve a PR or ≥ 50 % reduction in bone marrow blast from pretreatment baseline within 6 cycles of treatment)
- Subject requests discontinuation.
- Unacceptable toxicity that in the opinion of the investigator makes it unsafe to continue therapy.
- Clinically significant progressive disease.
- Investigator discretion.

Follow-Up at Treatment Discontinuation or Early Withdrawal

Subjects who discontinue treatment for any reason should complete end-of-treatment procedures when possible. End of treatment procedures will include a physical examination, CBC with differential and platelets, triplicate ECG, and full chemistry panel with ALT, AST, bilirubin (fractionated), alkaline phosphatase, potassium, magnesium, creatinine kinase (fractionated if >ULN), blood urea nitrogen (BUN), serum creatinine, troponin. A bone marrow aspiration and biopsy may be recommended only if non-response or progressive disease cannot be unequivocally diagnosed from peripheral blood. Although treatment will be discontinued at that time, all subjects who do not withdraw consent for follow-up, die, or become lost to follow-up, will remain on study for follow-up evaluations. Subject will be followed for toxicity for at least 30 days after the last protocol treatment. The 100-day follow-up visit (+ or – 5 days) will be scheduled as a clinic visits for clinical evaluation and physical examinations. If the subject cannot make it to the site for this visit, the required follow up treatment procedures may be done

with a local physician and the records forwarded to Investigator. The Investigator or delegate will contact the subject by telephone and get a verbal assessment of the subject's condition. The phone conversation will then be documented in the subject's charts.

8.9.2. Study Stopping Rules

The Principal Investigator and the Sponsor have the right to terminate this clinical study at any time. The principal investigator will be involved in any decisions regarding terminating the study, temporarily suspending enrollment, or stopping ongoing treatment with study treatment.

Reasons for terminating the clinical study or a study site's participation include, but are not limited to, the following:

- The incidence or severity of an adverse reaction related to treatment in this study or other studies indicates a potential health hazard to subjects
- Data recording is significantly inaccurate or incomplete
- Study site personnel are noncompliant with study procedures
- Pattern of noncompliance is observed

8.9.3. Protocol Violations and Deviations

Protocol violations are defined as significant departures from protocol-required processes or procedures that affect subject safety and benefit potential or confound assessments of safety or clinical activity. A protocol deviation is a departure from the protocol that does not meet the above criteria. Protocol violations or deviations may be grouped into the following classes:

- Enrollment criteria
- Study activities (missed evaluations or visits)
- Noncompliance with dose or schedule, including dose calculation, administration, interruption, reduction, or delay; or discontinuation criteria
- Investigational product handling, including storage and accountability
- Informed consent and ethical issues

8.10. ADVERSE EVENT MONITORING, RECORDING, AND REPORTING

8.10.1. Definitions and Procedures

Adverse Event (AE) is any untoward medical occurrence that may present during treatment with a pharmaceutical product, but which does not necessarily have a causal relationship with this treatment. **Adverse drug reaction** (ADR) is a response to a drug which is noxious and unintended, and which occurs at doses normally used in man for prophylaxis, diagnosis, or therapy of disease or for the modification of physiologic function.

Assessing causal connections between agents and disease is fundamental to the understanding of adverse drug reactions. In general, a drug may be considered a contributory cause of an adverse event if, had the drug not been administered, 1) the event would not have happened at all, 2) the event would have occurred later than it actually did, or 3) the event would have been less severe.

All subjects will be monitored for AEs during the study. AEs will be recorded in the subject's source documents from the first dose through 30 days after the last dose. All Grade 1-5 AEs will be captured within the Prometheus, irrespective of relatedness to study drug(s). Hematologic lab abnormalities will not be recorded or reported as adverse except for prolonged myelosuppression as defined by the NCI-CTCAE criteria specific for leukemia, e.g., marrow hypocellularity on day 42 or later (6 weeks) from start of therapy without evidence of leukemia (< 5% blasts), or that results in dose modifications, interruptions or meets the protocol definition of DLT or SAE.

Adverse Events Attribution

Attribution is the determination of whether an adverse event is related to a medical treatment or procedure.

Definite - the adverse event is clearly related to the investigational agent(s).

Probable - the adverse event is likely related to the investigational agent(s).

Possible - the adverse event may be related to the investigational agent(s).

Unlikely - The adverse event is doubtfully related to the investigational agent(s).

Unrelated - The adverse event is clearly NOT related to the investigational agent(s).

Adverse Events Severity

The severity of the adverse events (AEs) will be graded according to the U.S. Department of Health and Human Services, National Institutes of Health, National Cancer Institute, Common Terminology Criteria for Adverse Events (CTCAE), Version 5.0.

Events not included in the NCI CTCAE will be scored as follows:

- Grade 1: Mild: discomfort present with no disruption of daily activity, no treatment required beyond prophylaxis.
- Grade 2: Moderate: discomfort present with some disruption of daily activity, require treatment.
- Grade 3: Severe: discomfort that interrupts normal daily activity, not responding to first line treatment.
- Grade 4: Life Threatening: discomfort that represents immediate risk of death

The investigator (or physician designee) is responsible for verifying and providing source documentation for all adverse events, assigning the attribution and assessing the severity of the AE, the causal relationship between any events and the clinical study procedure, activities or device. Additionally, the Investigator is responsible for providing appropriate treatment for the event and for adequately following the event until resolution for all adverse events for subjects enrolled.

The Principal Investigator will sign and date the AE log per each subject at the completion of each course. Following signature, the AE log will be used as source documentation for the adverse events for attribution.

All participants will be registered in the Clinical Oncology Research System (CORe)

The Leukemia-specific Adverse Event Recording and Reporting Guidelines will be followed for the recording and reporting of adverse and serious adverse events.

Baseline events will be recorded in the medical history section of the case report form and will include the terminology event name, grade, and start date of the event. The medical history section of the case report form will serve as the source document for baseline events once signed and dated by the principal investigator.

Baseline events are any medical condition, symptom, or clinically significant lab abnormality present before the informed consent is signed

Hematologic laboratory abnormalities will not be recorded as baseline events for subjects with acute leukemia, myelodysplastic syndrome, chronic lymphocytic leukemia, or chronic myeloid leukemia in blast phase.

If exact start date is unknown, month and year or year may be used as the start date of the baseline event.

All grades of the adverse event will be captured per course or protocol defined visit date. For adverse events that increase or decrease in grade, the stop date should reflect the date that the event changed grade and a new start date should be added for the new grade.

Hematologic adverse events will not be recorded or reported for studies in subjects with acute leukemia, except for:

- Prolonged myelosuppression as defined by the NCI-CTCAE criteria specific for leukemia, e.g., marrow hypocellularity on day 42 or later (6 weeks) from start of therapy without evidence of leukemia (< 5% blasts), or that results in dose modifications, interruptions or meets the protocol definition of DLT or SAE.

Overdose is defined as the accidental or intentional administration of any dose of a product that is considered both excessive and medically important. All occurrences of overdose must be reported to the Sponsor within 24 hours of awareness. Overdose will be reported via eCRF.

An "excessive and medically important" overdose includes any overdose in which either an SAE, a non-serious AE, or no AE occurs and is considered by the Investigator as clinically relevant, i.e., poses an actual or potential risk to the subject.

Occupational exposures of DS-1594b must be reported to Daiichi Sankyo.

Serious adverse events will be reported according to institutional policy.

Protocol specific language regarding the recording and reporting of adverse and serious adverse events will be followed in the event of discordance between the protocol and Leukemia-specific adverse event recording and reporting guidelines.

8.10.2. Serious Adverse Events (SAEs)

Serious Adverse Event (SAE) Reporting Requirements for M D Anderson Sponsor Single Site IND Protocols

An adverse event or suspected adverse reaction is considered "serious" if, in the view of either the investigator or the sponsor, it results in any of the following outcomes:

- Death
- A life-threatening adverse event
- Inpatient hospitalization or prolongation of existing hospitalization
- A persistent or significant incapacity or substantial disruption of the ability to conduct normal life functions.
- A congenital anomaly/birth defect.

Important medical events that may not result in death, be life-threatening, or require hospitalization may be considered serious when, based upon appropriate medical judgment, they may jeopardize the patient or subject and may require medical or surgical intervention to prevent one of the outcomes listed in this definition. Examples of such medical events include allergic bronchospasm requiring intensive treatment in an emergency room or at home, blood dyscrasias or convulsions that do not result in inpatient hospitalization, or the development of drug dependency or drug abuse (21 CFR 312.32).

Important medical events as defined above, should be considered serious adverse events. Any important medical event can and should be reported as an SAE if deemed appropriate by the Principal Investigator or the IND Sponsor, IND Office.

All events occurring during the conduct of a protocol and meeting the definition of a SAE must be reported to the IRB in accordance with the timeframes and procedures outlined in "The University of Texas M. D. Anderson Cancer Center Institutional Review Board Policy on Reporting Adverse Events for Drugs and Devices".

Serious adverse events will be captured from the time of the first protocol-specific intervention, until 30 days after the last dose of drug, unless the participant withdraws consent.

Serious adverse events must be followed until clinical recovery is complete and laboratory tests have returned to baseline, progression of the event has stabilized, or there has been acceptable resolution of the event.

All SAEs, expected or unexpected/ initial or follow up, must be reported to the IND Office within 5 working days of knowledge of the event regardless of the attribution.

Death or life-threatening events that are unexpected, possibly, probably or definitely related to drug must be reported (initial or follow up) to the IND Office within 24 hours of knowledge of the event.

Additionally, any serious adverse events that occur after the 30 day time period that are related to the study treatment must be reported to the IND Office. This may include the development of a secondary malignancy.

The electronic SAE application (eSAE) will be utilized for safety reporting to the IND Office and MD Anderson IRB.

All events reported to the supporting company must also be reported to the IND Office

- Reporting to FDA:

Serious adverse events will be forwarded to FDA by the IND Sponsor according to 21 CFR 312.32.

It is the responsibility of the PI and the research team to ensure serious adverse events are reported according to the Code of Federal Regulations, Good Clinical Practices, the protocol guidelines, the sponsor's guidelines, and Institutional Review Board policy.

Adverse Events of Special Interest (AESI)

Additionally, the following events regardless of seriousness should be reported as outlined in Sections 6.2.3.1 and 6.2.3.2:

- QTc prolongation ≥ Grade 3, either serious or non-serious and whether or not causally related.
- Hepatic events meeting combination abnormalities [ALT or AST ≥3 × ULN with simultaneous TBL ≥2 × ULN] (potential Hy's Law case), both serious and non-serious and whether or not causally related.

8.10.3. Pregnancy

IND Sponsor must be notified of any female subject or partner of a male subject who becomes pregnant while receiving or within 3 months of discontinuing DS-1594b.

Although pregnancy is not technically an AE, all pregnancies must be followed to conclusion to determine their outcome. If a pregnancy is reported, the Investigator should inform the Sponsor within 24 hours of learning of the pregnancy.

This information is important for both drug safety and public health concerns. It is the responsibility of the Investigator, or designee, to report any pregnancy in a female subject or partner of a male subject. The Investigator should make every effort to follow the female subject or partner of a male subject (upon obtaining written consent from partner) until completion of the pregnancy and obtain complete pregnancy outcome information, including normal delivery and induced abortion. Any adverse pregnancy outcome, either serious or non-serious, should be reported in accordance with study procedures. If the outcome of the pregnancy meets the criteria for immediate classification as an SAE (i.e., post-partum complications, spontaneous or induced abortion, stillbirth, neonatal death, or congenital anomaly, including that in an aborted fetus), the Investigator should follow the procedures for reporting SAEs.

8.10.4. Investigator Communications with MDACC

Serious Adverse Events (SAEs) will be captured starting from the date of consent, irrespective of relatedness to study drug(s).

Serious adverse events will be captured from the time of the first protocol-specific intervention, until 30 days after the last dose of drug, unless the participant withdraws consent.

Serious adverse events must be followed until clinical recovery is complete and laboratory tests have returned to baseline, progression of the event has stabilized, or there has been acceptable resolution of the event.

All events reported to the supporting company must also be reported to the IND Office.

All events reported to DSI, must be reported to MDACC IND Office as well.

8.10.5. MDACC Reporting to Daiichi Sankyo, Inc.

All Serious Adverse Events, Adverse Events of Special Interest, overdoses and pregnancies must be reported by MDACC to the DSI Clinical Safety and Pharmacovigilance (CSPV) according to the terms outlined in the Alliance Pharmacovigilance Agreement.

DSI CSPV may query MDACC regarding cases in order to ensure appropriate documentation.

9. STATISTICAL CONSIDERATIONS

This is an open-label Phase 1/2 multi-arm study. The primary objective in Phase 1 is to determine the Maximum Tolerable Dose (MTD) and identify the Recommended Phase 2 Dose (RP2D) of DS-1594b.

Phase 2 will evaluate the safety and response rate within 3 months of therapy initiation with single agent or combinations of DS-1594b at the RP2D in the following 4 cohorts:

- Cohort A: R/R AML with MLL-rearrangement,
- Cohort B: R/R AML with NPM1 mutation,
- Cohort C: R/R AML treated with the combination of azacitidine and venetoclax with DS-1594b
- Cohort D: R/R ALL treated with the combination of miniHCVD with DS-1594b.

Up to 182 subjects will be enrolled in the study (approximately 66 subjects in Phase 1 (i.e., up to 42 for dose titration-BOIN design, 6 for food effect sub-studies, and up to 18 for refining RP2D at safe doses below or at the MTD and DDI sub-studies) and up to 26 subjects per cohort in Cohorts A-and B and up to 32 subjects per cohort in Cohorts C and D in Phase 2).

9.1. Phase 1

The primary objective of Phase 1 is to determine the MTD and RP2D of DS-1594b when administered as a single agent in subjects with R/R AML or R/R ALL, including subjects with or without MLLr or NPM1m using a Bayesian optimal interval (BOIN) design (Liu and Yuan, 2015; Yuan et al., 2016) to find the MTD. The details of the BOIN design to be used in the Phase 1 are outlined in Section 3.2.

According to the BOIN design, cohorts of at least 3 subjects will be treated at each dose level. If a dose level is found to be safe by the BOIN design, we will escalate to the next higher dose

level. Evaluation for above mentioned BOIN rule will be done for each dose level and dosing regimen (i.e., QD vs BID) separately based on the cumulative number of DLT evaluable subjects treated in that dose level. In case the multiple dosing regimens are initiated simultaneously and parallel enrollment is allowed in the multiple dosing regimens, the evaluation for BOIN rule for each dose level and dosing regimen would be made separately. Up to 9 additional subjects may be enrolled at each dose level that has already been deemed to be safe with the intent to gain additional information on safety and efficacy at different dose levels, to guide selection of optimal biologically effective RP2D. Although all R/R ALL and R/R AML subjects, irrespective of mutations, will be allowed during the Phase 1 dose-escalation it is suggested that at least 3 subjects with MLLr or NPM1m are included at each dose level that is anticipated to be clinically active, to allow for optimal evaluation of safety and efficacy in the presumed target population to guide selection of the optimal RP2D. As described in Section 3.2, we will use the same the elimination boundaries in Table 5 for the toxicity monitoring in the expansion cohort(s).

9.2. Phase 2

The primary endpoint of cohorts A-C is the achievement of CR + CRh within 3 months of therapy initiation in subjects with R/R AML with MLLr or NPM1m. The primary endpoint of cohort D is the achievement of CR + CRi within 3 months of therapy initiation in subjects with R/R ALL with MLLr. Up to 26 subjects will be enrolled per cohort in Cohorts A and B and up to 32 subjects will be enrolled per cohort in Cohorts C and D. Each cohort will be analyzed separately. Treatment will be considered encouraging if the CR/CRh rate (Cohorts A, B, and C) or CR/CRi rate (Cohort D) rate would reach at least 40%, an improvement over the historical rate by more than 15%. The study will monitor futility and toxicity using the Bayesian method of Thall, Simon and Estey[27].

9.3. Futility and Toxicity Monitoring

The historical data suggest a response rate of 25% in subjects with R/R AML or R/R ALL with NPM1m or MLLr using a variety of different therapies[4,10, 19, 22], and the target response rate is 40% with acceptable safety. The response rate is defined as CR + CRh rate within 3 months of therapy initiation in cohorts A, B and C and CR/CRi rate within 3 months of therapy initiation in Cohort D. The cohort will be stopped early if the data suggest that:

$$Pr(P_E <= 0.40 | data) > 0.95, \text{ or}$$

$$Pr(P_T > 0.2 | data) > 0.85.$$

where P_E is the response rate and P_T is the probability of DLT for experimental treatments, respectively. We assume P_E and P_T follow a priori ~ beta (0.5, 0.5). That is, if at any time during the study we determine that there is more than 95% chance that the response rate would be at less than 40%, the cohort will be stopped due to futility. Or if at any time during the study, we determine that there is more than 85% chance that the toxicity rate is more than 20%, the cohort will be stopped due to toxicity. DLT observations from IPDE will not be used in toxicity monitoring.

The futility and toxicity monitoring will be conducted separately for each of four cohorts, starting once the 10th subject in a cohort has been evaluated, and thereafter in groups of 5

subjects, except the last group. The design parameters are set the same for cohorts A-D, each with a maximum of 26 subjects per cohort, excluding the safety run-in period for the first 6 subjects in each of Cohorts C and D (Section 3.3).

The stopping boundaries for futility and toxicity based on these assumptions and monitoring conditions are provided in Table 25. The operating characteristics are summarized in Table 26 and in Table 27 for futility and toxicity monitoring, respectively. Table 28 presents the overall early stopping probabilities for futility and toxicity monitoring. Bayesian Toxicity Monitoring software and Bayesian Efficacy monitoring with Posterior Probability software on https://trialdesign.org were used for the design of toxicity and futility monitoring.

If the study arm is not stopped early and 26 subjects have been treated and evaluated in each cohort, assuming 11 of the 26 subjects achieve CR+CRh (Cohorts A, B, C) or CR+CRi (Cohort D), then the 95% credible interval for CR+CRh (Cohorts A-C) or CR+CRi (Cohort D) rate will be (25%, 61.3%).

Table 25: Stopping Boundaries for Toxicity and Futility Monitoring in Each of Cohorts A-D

	**Toxicity Stopping Boundaries**		**Futility Early Stopping Boundaries**	
Number of Subjects (inclusive)	Num DLTs (inclusive) are considered too toxic	Actions for toxicity monitoring	Num. Responses (inclusive) are considered futile	Actions for futility monitoring
10	4 - 10	Early stopping	0 - 1	Early stopping
15	5 - 15	Early stopping	0 - 2	Early stopping
20	6 - 20	Early stopping	0 - 4	Early stopping
26	8 - 26	Reach to Nmax	0 - 6	Reach to Nmax

9.3.1. IND Office Futility and Safety Monitoring

Safety oversight will be performed by the SRC, the meeting minutes will be submitted to the IND Office Medical Affairs & Safety group upon availability.

Table 26: Operating Characteristics for Futility Monitoring in each of Cohorts A-D

**True Response Rate**	**Early stop probability for futility**	**Average sample size**
0.1	0.9633	12.29
0.2	0.6713	17.72
0.3	0.2906	22.57
0.4	0.0811	25.00
0.5	0.0153	25.79
0.6	0.002	25.97

Table 27: Operating Characteristics for Toxicity Monitoring in each of Cohorts A-D

True DLT rate	Probability of Early Stop for toxicity	Average Sample size
0.1	0.0235	25.70
0.2	0.2509	22.92
0.3	0.636	17.84
0.4	0.8954	13.53
0.5	0.9834	11.22

Table 28: Overall early stopping probabilities for Futility and Toxicity Monitoring in each of Cohorts A-D

True DLT rate	True Response Rate	Overall probability of early stop
0.1	0.2	0.6790
0.1	0.4	0.1027
0.1	0.6	0.0255
0.2	0.2	0.7538
0.2	0.4	0.3117
0.2	0.6	0.2524
0.4	0.2	0.9656
0.4	0.4	0.9039
0.4	0.6	0.8956

Table 29 presents the estimation precision (e.g., 95% credible intervals) under various scenarios with different numbers of observed responses, given that a cohort is not stopped early and 26 subjects have been enrolled. For example:

- if 11 of the 26 subjects achieve the response (CR + CRh for Cohorts A-C and CR+Cri for Cohort D), the 95% credible interval of the response rate will be (25%, 61.3%).
- if 8 of the 26 subjects achieve the response, the 95% credible interval of the response rate will be (15.8%, 49.8%).

Table 29: 95% Credible Intervals Under Various Scenarios with Different Numbers of Responses

Number of responses	Empirical response rate	95% Credible interval
6	23.1%	(10.3%, 41.5%)
8	30.8%	(15.8%, 49.8%)
10	38.5%	(21.8%, 57.6%)
11	42.3%	(25.0%, 61.3%)
12	46.2%	(28.2%, 64.9%)
14	53.8%	(35.1%, 71.8%)
16	61.5%	(42.4%, 78.2%)
18	69.2%	(50.2%, 84.2%)

9.4. Statistical Analysis Plan

9.4.1. Analysis Sets

9.4.1.1. Efficacy Analysis Set

The efficacy analysis set is all subjects with MLLr or NPM1m who received at least one dose of DS-1594b. Each cohort will be analyzed separately for efficacy.

9.4.1.2. Safety Analysis Set

Safety analysis set is all subjects who received at least one dose of DS-1594b.

9.4.1.3. DLT Evaluable Set

The DLT Evaluable Set will include all subjects in the Safety Analysis Set from Dose Escalation who experienced a DLT during the DLT evaluation period, or who has not had a DLT and has received at least 75% of DS-1594b target doses and has completed the DLT evaluation period.

9.4.1.4. PK Analysis Set

The Pharmacokinetic Analysis Set (PAS) will include all subjects who receive at least one dose of DS-1594b and have at least one evaluable PK sample. The PAS will be used for summaries of PK data as well as for listing of derived PK parameters. The definition of PK evaluability for the DDI sub-study and food effect sub-study are described in Section 3.2.3.3 and Section 3.2.4.3, respectively.

9.4.2. Analysis Convention

General rule

Baseline is defined as the last non-missing value before the first dosing of study drug. The day of treatment would be considered as Day 1. Both the scheduled and unscheduled assessments will be used in the scope of analysis. For Phase 1, results will be presented by dose level and in total excluding subjects enrolled in the two sub-studies. The results from the DDI sub-study and food effect sub-study will be presented separately. For Phase 2, results will be presented by Cohort and in total, unless otherwise stated.

Visit window

For the summary at scheduled post-treatment visits, a continuous visit window would be used. For example, for visit scheduled at Day X, the visit window starts on day prior to $X_L = [(X+Y)/2]_+$ and ends on Day $X_U = [(X+Z)/2]_+$ where Y is the scheduled day of prior visit, Z is the scheduled day of next visit and $[x]_+$ is the least integer greater than or equal to x. The visit window for a given scheduled post-treatment visit can be different for different assessments depending on schedule assessments as not all the assessments are planned at each visit.

Multiple assessments

For multiple assessments within a particular visit window the assessment closest to the scheduled day of visit would be used. If there are multiple equi-distant assessments within a visit window, the assessment collected after the scheduled day of visit would be used. If there are multiple assessments, on a particular day, the worst value would be used.

9.4.3. Analysis of Subject Disposition, Demography and Baseline Characteristics, and Medical History

Subjects disposition (e.g., number of subjects screened, treated, completed study drug, completion of study, reason for discontinuation) will be summarized using counts and percentages. Subject demography and relevant baseline characteristics and medical history will be summarized descriptively for all treated subjects (i.e., the safety analysis set).

9.4.4. Analysis of Exposure of Study Drug

Study treatment exposure and treatment duration will be summarized using descriptive statistics for the Safety Analysis Set. In addition, the total number of cycles initiated will be summarized using descriptive statistics. Number and percentage of subjects with and without dose reductions or interruptions will also be summarized. In addition, cumulative dose and dose intensity will also be summarized.

The following definitions would be used:

Exposure (in days): last dose date – first dose date + 1

Treatment duration (in days): last dose date – first dose date + 30

Cumulative dose: Total amount dose actually taken

Dose intensity: Cumulative dose/ Exposure

9.4.5. Analysis of Prior and Concomitant Medications

Prior and concomitant medications will be coded using the World Health Organization drug dictionary (WHO-DD). Concomitant medications will be summarized by ATC2 class and preferred term for the Safety Analysis Set. Prior medications will be summarized for the Safety Analysis Set. Within each level of summarization, a subject will be counted once if he/she takes one or more medications.

Prior medications are defined as those with a start date prior to the date of first dose of study drug. Concomitant medications are defined as those with a start date greater than or equal to the date of first dose of study drug, or with a start date prior to the date of first dose of study drug and a stop date either after the date of first dose of study drug or marked as "ongoing" or "continuing". Medications taken prior to the first dose of study drug, but with a missing stop date or with a stop date either on or after the date of the first dose of study drug or marked as "ongoing" or "continuing" will also be considered concomitant medications for the summary. Medications started after the 40-day visit or after start of new anticancer therapy are not considered as concomitant medications. A listing of prior and concomitant medications by subject will also be provided.

9.4.6. Efficacy Analyses

The efficacy analyses will be carried out for both the Efficacy Analysis Set and the Safety Analysis Set. Subjects with prior treatment with other menin inhibitors will be analyzed separately for efficacy.

For the primary efficacy analysis, the primary efficacy outcome (CR + CRh rate for Cohorts A, B, C and CR+CRi rate for Cohort D) will be estimated along with the 95% credible interval for each cohort. If the study cohort is not stopped early, assuming 11 of the 26 subjects achieve the response (CR + CRh for Cohorts A, B, C and CR+CRi for Cohort D), the 95% credible interval for the response rate will be (25%, 61.3%). The same method will be used for other binary efficacy outcomes in Phase 1 and Phase 2, including rate of durable transfusion independence, the 4- and 8-week mortality rates, the rate of subjects able to proceed to HSCT, and the MRD rate at the first response and at the best response. The time to event outcomes, including DOR, time to first response, time to best response, median duration to HSCT. EFS and OS, will be estimated using the Kaplan-Meier method. For the exploratory analysis, logistic regression models and Cox proportional hazards models will be fitted to correlate clinical factors, expressions of immune markers with efficacy outcomes. OS time will be calculated from the date of the treatment start to the date of death or to the date of last follow-up if patients are alive at the time of data collection. Patients alive will be censored at the analysis. EFS will be

calculated from the date of start treatment to the date of disease relapse or date of death whichever occurs first, or to the date of last follow-up for patients who are alive and have no relapsed disease at the time of data collection. Patients who are alive and have no relapsed disease will be censored at the analysis.

9.4.7. Safety Analyses

Safety analyses will be performed in the safety population. AEs that occur or worsen relative to the pre-treatment state after the start of study treatment and within 30 days of the last dose of study drug (TEAEs) will be tabulated. AEs will be coded using the Medical Dictionary for Regulatory Activities (MedDRA) terminology and tabulated by body organ group using the MedDRA system organ class (SOC) and by AE term using preferred terms (PTs).

Safety evaluation will include DLTs, serious adverse events, treatment-emergent adverse events, physical examination findings (including ECOG performance status), vital signs measurements, standard clinical laboratory parameters, and ECG parameters (including QTcF).

Safety analyses in general will be descriptive and will be presented in tabular format with the appropriate summary statistics by dose level and in total for Phase 1, and by cohort and in total for Phase 2, unless otherwise stated. Only the treatment emergent assessments (i.e., assessments within the 30 days of last study drug dose) would be used in the analyses unless otherwise specified.

The summary of DLTs in the Dose Escalation parts will be performed based on the DLT Evaluable Set. All other safety summaries and analyses will be performed based on the Safety Analysis Set and subjects will be analyzed according to the actual treatment they received.

9.4.7.1. Dose-Limiting Toxicities

For Phase 1 (Dose Escalation), the number of DLTs identified among the DLT evaluable subjects in the DLT Evaluable Set will be listed and summarized for each dose cohort and in total by the worst NCI-CTCAE grade, system organ class (SOC), and preferred term (PT).

9.4.7.2. Adverse Events

Adverse events will be assigned grades based on NCICTCAE v5.0.

A TEAE is defined as those AEs with start or worsening date during the treatment period (from first dose date until 30 days after the last dose of study medication). It includes AEs having been absent at pre-treatment; or reemerges during treatment, having been present at baseline, but stopped prior to treatment; or worsens in severity after starting treatment relative to the pre-treatment state, when the AE is continuous.

9.4.7.2.1. Summary of Treatment-Emergent Adverse Events

The number and percentage of subjects with the following TEAEs will be summarized:

1. TEAEs by SOC, preferred term, and worst CTCAE grade;
2. Study-drug-related TEAEs by SOC, preferred term, and worst CTCAE grade;

3. Serious TEAEs by SOC, preferred term, and worst CTCAE grade;
4. Study-drug-related serious TEAEs by SOC, preferred term, and worst CTCAE grade;
5. TEAEs associated with drug discontinuation by SOC and preferred term
6. TEAEs associated with dose reduced by SOC and preferred term
7. TEAEs associated with drug interrupted by SOC and preferred term
8. TEAEs associated with death by SOC and preferred term

The number and percentage of subjects with AEs will be also summarized by PT only, if applicable. A by-subject TEAE data listing including, but not limited to, verbatim term, preferred term, SOC, CTCAE grade, and relationship to study drug will be provided.

Listings will be provided for deaths, SAEs, and AEs associated with discontinuation of study drug. A by-subject AE (including treatment-emergent) data listing including, but not limited to, verbatim term, preferred term, system organ class, NCI-CTCAE grade, and relationship to study drug will be provided.

9.4.7.2.2. Adverse Events of Special Interest

The adverse events of special interest (AESI) will be categorized by Standardized MedDRA Queries (SMQs) or grouping of MedDRA terms in this study and will include the following two categories: TdP/other ventricular arrhythmias and Hepatotoxicity. The search criteria for each AESI category are specified in Table 30.

Table 30: AESI Categories and SMQ Search Criteria

AESI Category	SMQ Search Criteria
TdP/other ventricular arrhythmias	All PTs from Torsade de pointes/QT prolongation SMQ (broad) plus PTs Fall, presyncope, agonal rhythm, arrhythmia, cardiac flutter, paroxysmal arrhythmia
Hepatotoxicity	All PTs from:<br>Cholestasis and jaundice of hepatic origin (SMQ) (narrow)<br>Hepatic failure, fibrosis and cirrhosis and other liver damage-related conditions (SMQ) (narrow)<br>Hepatitis, non-infectious (SMQ) (narrow)<br>Liver related investigations, signs and symptoms (SMQ) (narrow)

The preferred terms (PT) in each AESI category, as well as the AESI categories in the table may be updated. A complete list of AESI categories with PT terms will be reported in the CSR.

The following AESI summaries will be provided:

- TEAEs by AESI category and PT (any TEAEs, Grade ≥3, fatal, associated with study drug discontinuation)
- Time to onset of earliest TEAEs by AESI category

9.4.7.2.3. Clinical Laboratory Evaluations

Descriptive statistics will be provided for selected clinical laboratory test results (hematology and blood chemistry) and changes from baseline by scheduled time of evaluation, including the End-of-Treatment Visit, maximum post-treatment value, and minimum post-treatment value. Both scheduled and unscheduled post-treatment values will be considered for the summaries of the maximum and minimum post-treatment values.

Abnormal laboratory results will be graded according to NCI-CTCAE Version v5.0, if applicable. A shift table, presenting the 2-way frequency tabulation for baseline and the worst post-treatment value according to the NCI-CTCAE grade, will be provided for selected clinical laboratory tests (ALT, AST, Total Bilirubin, ALP, and Creatinine). Both scheduled and unscheduled post-treatment values during the treatment period will be considered.

The number and percentage of subjects with the following potentially clinically relevant abnormal liver function test will be summarized by dose cohort and in subtotal for each part:

- ALT ≥3 x ULN, ≥5 x ULN, ≥10 x ULN, and ≥20 x ULN
- AST ≥3 x ULN, ≥5 x ULN, ≥10 x ULN, and ≥20 x ULN
- Total bilirubin ≥2 x ULN
- Potential Hy's Law cases: ALT or AST >3 x ULN, total bilirubin ≥2 x ULN, and ALP <2 x ULN

Mean absolute neutrophil count and platelet count over time by dose cohort for Part 1 as well as individual absolute neutrophil count and platelet count over time for Part 1A subjects will be plotted.

All clinical laboratory data will be listed, and values deemed clinically significance will be flagged.

9.4.7.3. Vital Signs

Descriptive statistics will be provided for the vital signs measurements by scheduled time of evaluation (including the EOT visit), as well as for the change from baseline. In addition, the change from baseline will be presented for the maximum and minimum post-treatment values.

9.4.7.4. ECG

Electrocardiogram parameters (PR, RR, QRS, QT, QT_cB, and QT_cF) will be summarized using descriptive statistics for actual values and for changes from baseline by scheduled time of evaluation, including the EOT Visit and the maximum post-treatment value. Both scheduled and unscheduled post-treatment values will be considered for summaries of the maximum

post-treatment values. The corrected QT intervals using Bazett's and Fridericia's formula will be calculated as follows: $QT_cB = QT/(RR)^{1/2}$ and $QT_cF = QT/(RR)^{1/3}$.

The incidence of notable ECG changes in maximum absolute QT, QT_cF, and QT_cB over all post-treatment evaluations will be summarized using the following intervals:

- New > 450 and ≤ 480 ms;
- New > 480 and ≤ 500 ms;
- New > 500 ms.

The incidence of notable ECG changes in QT, QT_cF, and QT_cB maximum changes from baseline over all post-treatment evaluations will be summarized with the following intervals:

1. 30 and ≤ 60 ms;
2. > 60 ms;

All triplicate ECG measurements at a particular timepoint will be averaged prior to analysis and summarization.

9.4.8. Dosing and Extent of Exposure

The following items of DS-1594b administration will be summarized.

1. Treatment duration (day)
   = Last dose date – First dose date + 1
2. Study duration (day)
   = Date of the Follow-up visit – First dose date + 1
3. Total amount of DS-1594b taken (mg)
4. Dose intensity (mg)
   = Total amount of doses taken / (Treatment duration)
5. Relative dose intensity (%)
   = Actual Dose intensity / Planned dose intensity * 100

In addition, the total number of cycles initiated will be summarized using descriptive statistics.

In Phase 2, DS-1594b dosing status will be summarized to show the number and percentage of subjects with and without dose reductions or interruptions. For subjects with dose reductions or interruptions, the reasons will be provided. A listing of dose escalation subjects with dose reductions or interruptions will be provided.

For the combination-treatment cohorts C & D in Phase 2, the exposure of the combination drugs azacitidine and venetoclax, and mini-HCVD will be summarized separately.

All study drug administration data will be listed by subject by study Phase.

9.4.9. PK Analyses

DS-1594b PK parameters will be determined using non-compartmental method(s) and fitting of the actual values to a population PK model. The single-dose dose and multiple dose PK parameters listed in Section 8.4 will be estimated and reported, as appropriate.

Descriptive statistics (n, mean, SD, CV%, median, minimum, and maximum) will be performed on all PK parameters by cohort and study day. Geometric means and geometric CV% will be presented for Cmax, AUClast, AUCinf, AUCtau, Ctrough, Cavg, CL/F, CLss/F, Vz/F and AR. Medians and ranges will be presented for Tmax, Tlast, t1/2, and t1/2,ar.

Descriptive statistics (n, mean, SD, median, geometric mean, CV%, geometric CV%, minimum, and maximum) will be presented for plasma concentrations by cohort.

A graphical presentation of the plasma concentration profiles will also be provided by cohort using the arithmetic mean (+/- SD) and geometric mean values at each scheduled time point for full PK collection profile on Cycle 1 Day 1, Cycle 1 Day 8, Cycle 1 Day 18 (DDI sub-study and food effect sub-study) and Cycle 2 Day 10 (DDI sub-study). The trough concentration -time profile will also be plotted by cohort.

Concentration values below the lower limit of quantitation (LLOQ) will be displayed in listings as zero with a flag and handled as zero in any calculations of summary statistics but handled as missing for the calculation of the geometric means and their CV%. Any missing PK parameter data will not to be imputed.

9.4.9.1. Food Effect Sub-Study

The effect of meal on the steady-state PK of DS-1594b will be evaluated in the food effect sub-study when:

- DS-1594b is administered orally twice daily or daily with a standard meal from Cycle 1 Day 1 to Cycle 1 Day 8.
- DS-1594b is administered orally twice daily or daily under fasting condition from Cycle 1 Day 9 to Cycle 1 Day 18.

The analysis will consider the primary PK parameters AUCtau and Cmax of DS-1594b on Cycle 1 Day 8 and Cycle 1 Day 18. A mixed-effects model will be used with treatment period (fed vs. fasting) as a fixed effect and subject as a random effect on log-transformed PK parameter data, and the results will be presented at original scale after back-transformation. The geometric mean of DS-1594b PK parameters (as estimated by the mixed effect model) will be calculated and the geometric mean ratio of DS-1594b PK parameters (Cycle 1 Day 18 relative to Cycle 1 Day 8) will be reported with 90% CIs.

9.4.9.2. DDI Sub-Study

The effect of multiple doses of strong CYP3A inhibitor on the steady-state PK of DS-1594b will be evaluated in the DDI sub-study when:

- DS-1594b is administered orally twice daily or daily alone from Cycle 1 Day 1 to Cycle 1 Day 8.

- DS-1594b is administered oral twice daily or daily with strong CYP3A inhibitor (posaconazole or vericonazole) from Cycle 1 Day 9 to Cycle 1 Day 18 (or Cycle 2 Day 1 to Cycle 2 Day 10).

The analysis will consider the primary PK parameters AUCtau and Cmax of DS-1594b on Cycle 1 Day 8 and Cycle 1 Day 18 (or Cycle 2 Day 10). A mixed-effects model will be used with treatment period (with CYP3A inhibitor vs. without CYP3A inhibitor) as a fixed effect and subject as a random effect on log-transformed PK parameter data, and the results will be presented at original scale after back-transformation. The geometric mean of DS-1594b PK parameters (as estimated by the mixed effect model) will be calculated and the geometric mean ratio of DS-1594b PK parameters (Cycle 1 Day 18 (or Cycle 2 Day 10) relative to Cycle 1 Day 8) will be reported with 90% CIs.

9.4.10. PK/PD Analyses

Additional exploratory analyses may be conducted to identify possible relationships of appropriate observed or population PK-derived PK parameters with clinical efficacy, safety variables, biomarkers, or dosing change as appropriate.

To assess the effect of DS-1594b on the QT interval, QTc changes from baseline will be plotted against the time-matched PK concentration, for each QTc parameter. In addition, a linear mixed effects model may be used to analyze changes from baseline QTc versus DS-1594b PK concentrations, including covariates such as baseline QTc, age, gender, race. Details will be described in a separate modeling plan.

10. APPENDICES

Table 31: List of Prohibited QT Prolongation Drugs

**Prohibited medications causing QTc prolongation**			
**Antiarrhythmic:**	**Anticancer:**	**Antibiotic:**	**Antianginal:**
amiodarone	arsenic trioxide	azithromycin	bepridil
disopyramide	vandetanib	clarithromycin*	
dofetilide	oxaliplatin	erythromycin*	**Antipsychotic:**
flecainide	**Antihistamine:**	moxifloxacin	chlorpromazine
ibutilide	astemizole*	sparfloxacin	haloperidol*
procainamide	terfenadine*	levofloxacin	mesoridazine
quinidine*		ciprofloxacin	pimozide
sotalol	**Antimalarial:**	grepafloxacin	thioridazine
dronedarone*	chloroquine	**Antinausea:**	sulpiride
	halofantrine	domperidone	levomepromazine
**Antilipemic:**	hydroxychloroquine	droperidol	**Opiate agonist:**
probucol		ondansetron	levomethadyl
**Antidepressant:**		**GI stimulant:**	methadone
citalopram	**Anti-infective:**	cisapride*	cocaine
escitalopram	pentamidine		**Other:**
			anagrelide
			sevoflurane
			cilostazol
			donepazil
			papaverine
			propofol

Note: *CYP3A substrate
Source: The most updated list is available on website https://www.crediblemeds.org/healthcare-providers/drug-list

Table 32: Prohibited Medications That Are Strong Inhibitors or Inducers of CYP3A, or CYP3A/P-Gp and CYP2C19 Substrates with Narrow Therapeutic Indices

**CYP3A4/5 substrates and/or P-gp substrates with narrow therapeutic index**			
astemizole*	diergotamine	pimozide*	alfentanil
cisapride*	ergotamine	quinidine*	terfenadine*
cyclosporine	fentanyl	tacrolimus	sirolimus
digoxin	paclitaxel		
**CYP2C19 substrates with narrow therapeutic index**			
(S)-mephenytoin			
**Strong CYP3A inhibitors**			
**Macrolide antibiotics:**	**Antivirals:**	**Antifungals:**	**Others:**
clarithromycin**	indinavir	itraconazole**	conivaptan
telithromycin	lopinavir/ritonavir	ketoconazole	
troleandomycin	nelfinavir	posaconazole	mibefradil
	ritonavir**	voriconazole	nefazodone
	indinavir/ritonavir**		
	saquinavir		
	saquinavir/ritonavir**		
	telaprevir**		
	tipranavir/ritonavir**		
	danoprevir/ritonavir**		
	elvitegravir/ritonavir**		
	boceprevir		
	cobicistat		
**Strong CYP3A/5 inducers**			
avasimibe	carbamazepine	phenobarbital	phenytoin
rifabutin	rifampin	St. John's wort	mitotane

* Compounds known to increase QTc interval that are also primarily metabolized by CYP3A4/5.
** Compounds known to have P-gp inhibitory effect
Reference:
University of Washington's Drug Interaction Database
http://www.fda.gov/Drugs/DevelopmentApprovalProcess/DevelopmentResources/DrugInteractionsLabeling/ucm093664.htm#4

Table 33: List of medications to be used with caution

**Moderate CYP3A4/5 inhibitors***			
netupitant	darunavir	grapefruit juice	crizotinib
atazanavir/ritonavir	darunavir/ritonavir	aprepitant	tofisopam
amprenavir	diltiazem	casopitant	lomitapide
atazanavir	verapamil	cimetidine	
	imatinib	nilotinib	
**Moderate CYP3A4/5 inducers***			
bosentan	efavirenz	etravirine	modafinil
nafcillin	lersivirine	talviraline	tipranavir
	lopinavir		
**P-gp inhibitors (not strong CYP3A inhibitors)**			
carvedilol	lapatinib	propafenone	ranolazine
verapamil			
**BCRP substrates**			
atorvastatin	daunorubicin	doxorubicin	imatinib
methotrexate	mitoxantrone	pitavastatin	rosuvastatin
SN-38 (irinotecan)	ethinyl estradiol	simvastatin	sulfasalazine
sofosbuvir	topotecan		
**OCT1/2 substrates**			
metformin	phenformin	6-beta-hydroxycortisol	amantadine
carboplatin	cisplatin	histamine	lamivudine
linagliptin	oxaliplatin	oxybutynin	picoplatin
pramsorafenib	tropisetron	trospium	varenicline
umeclidinium			
**P-gp substrates**			
aliskiren	ambrisentan	atorvastatin	azithromycin
cerivastatin	colchicine	cyclosporine	dabigatran
docetaxel	domperidone	doxorubicin	fexofenadine
lapatinib	linezolid	loperamide	maraviroc
nevirapine	proguanil	ritonavir	ranolazine
simvastatin	sofosbuvir	ticagrelor	voclosporin

**Gastric reducing agents (proton pump inhibitors)**			
esomeprazole	lansoprazole	omeprazole	pantoprazole
rabeprazole			

*Subjects enrolled in DDI and food effect sub-studies will have moderate CYP3A inhibitors and inducers prohibited (see Appendix Table 34).
References:
University of Washington's Drug Interaction Database
http://www.fda.gov/Drugs/DevelopmentApprovalProcess/DevelopmentResources/DrugInteractionsLabeling/ucm093664.htm#4

Table 34: Additional Prohibited Medications for Subjects Enrolled in DDI and Food Effect Sub-Studies

**Moderate CYP3A4/5 inhibitors**			
netupitant	darunavir	grapefruit juice	crizotinib
atazanavir/ritonavir	darunavir/ritonavir	aprepitant	tofisopam
amprenavir	diltiazem	casopitant	lomitapide
atazanavir	verapamil	cimetidine	
fluconazole	imatinib	nilotinib	
**Moderate CYP3A4/5 inducers**			
bosentan	efavirenz	etravirine	modafinil
nafcillin	lersivirine	talviraline	tipranavir
	lopinavir		
**Gastric reducing agents (proton pump inhibitors)**			
esomeprazole	lansoprazole	omeprazole	pantoprazole
rabeprazole			
References:<br>University of Washington’s Drug Interaction Database<br>http://www.fda.gov/Drugs/DevelopmentApprovalProcess/DevelopmentResources/DrugInteractionsLabeling/ucm093664.htm#4			

Table 35: Phase 1 Lead-in Period Schedule of Events (QD Dosing)

Phase 1 Lead-in Period Schedule of Events													
**Procedure**	**SCR**	**Lead-in Week 1**			**Lead-in Week 2**	**Lead-in Week 3**		**Lead-in Week 4**					
**Day**	**-14**	**1**	**2**	**5**	**1**	**1**	**7**	**1**	**7**	**EOT**	**30D FU**	**LTFU**	
**Window (Days)**							**±4**		**±4**	**±3**	**±7**	**±14**	**Comment**
**Informed Consent**	X												Prior to study procedures
**Bone Marrow Aspirate/Biopsy**	X						X*		X*	X			If lead-in period is ≥ 21 days, an optional bone marrow aspiration/biopsy and/or periferal blood sample may be collected at the end of the lead-in period (± 4 days) for the purpose of correlative studies
**Eligibility**	X												
**Demographics, Medical History, Disease History**	X												WHO classification, cytogenetics, mutations, prior therapy
**Vital Signs**	X	X	X	X	X	X		X		X			Blood pressure, pulse rate, respiratory rate, and temperature taken ~5 min prior to ECG measurements
**ECG (in triplicate) by 12-lead or Holter**	X	X	X	X	X	X		X		X			Triplicate ECGs ≥1 minute apart, supine/semi-recumbent. Prior to PK<br>See Section 8.5 and Table 23 for timing of ECGs for Cohorts with Lead-in Period (Table 23 contains ECG assessment for both Lead-in Period and Target Dose Treatment Period).
**Height**	X												Screening procedures should be done 3 days before first dose.
**Weight**	X	X			X	X		X		X			
**BSA**	X												Calculate by Mosteller formula
**SpO2**	X	X			X	X		X		X			
**Physical Exam**	X	X			X	X		X		X			
**ECOG PS**	X	X			X	X		X		X			
**ECHO/MUGA**	X												≤30 days prior to first dose
**SAEs**	X	continuously											

Phase 1 Lead-in Period Schedule of Events													
**Procedure**	**SCR**	**Lead-in Week 1**			**Lead-in Week 2**	**Lead-in Week 3**		**Lead-in Week 4**					
**Day**	**-14**	**1**	**2**	**5**	**1**	**1**	**7**	**1**	**7**	**EOT**	**30D FU**	**LTFU**	
**Window (Days)**							**±4**		**±4**	**±3**	**±7**	**±14**	**Comment**
**AEs**		continuously											
**Prior Medications, Non-Drug Therapies, and Radiotherapy**	X												
**Concomitant Medications, Non-Drug Therapies, and Radiotherapy**		continuously											
**Hematology, INR, Chemistry**	X	X	≥3 times weekly							X			Screening procedures should be done 3 days before first dose. CBC with Differential, PLT, Serum Creatinine, Electrolytes, Glucose, Uric acid, calcium, magnesium, BUN
**Ferritin, LDH, CRP, Fibrinogen, PT/PTT, and Cytokine panel (IL-6, IFN-γ, and TNF-α)**	X	X	≥2 times weekly							X			Monitor after Cycle 1, if clinically indicated.
**Hepatic Labs**	X	X	≥3 times weekly							X			AST, ALT, Alk phos, Total Bilirubin (fractionated)
**Creatinine Kinase**	X	X	≥1 time weekly							X			Fractionated if >ULN
**Troponin**	X												Monitor on treatment, if clinically indicated
**Urinalysis**	X	X	monthly							X			Screening procedures should be done 3 days before first dose.
**Pregnancy Test**	X	X	monthly							X	X		For women of childbearing potential, document the results of a negative urine or serum pregnancy test within 72 hours before first dose and monthly while on treatment.
**Plasma alpha-MSH**	X									X			* Once every 3 cycles beginning at Cycle 3
**Fasting morning ACTH, aldosterone, renin, and cortisol**	X									X			* Once every 3 cycles beginning at Cycle 3
**HIV Ab Test**	X												If required by local regulations; ≤28 days of first dose
**HBsAg, HC Ab**	X												≤28 days of first dose

**Phase 1 Lead-in Period Schedule of Events**													
**Procedure**	**SCR**	**Lead-in Week 1**			**Lead-in Week 2**	**Lead-in Week 3**		**Lead-in Week 4**			**30D**		
**Day**	**-14**	**1**	**2**	**5**	**1**	**1**	**7**	**1**	**7**	**EOT**	**FU**	**LTFU**	
**Window (Days)**							**±4**		**±4**	**±3**	**±7**	**±14**	**Comment**
**PK**		X	X	X	X	X		X					See Table 17 (QD dosing with lead-in period for timing of PK sampling (Table 17 contains PK assessment for both Lead-in Period and Target Dose Treatment Period).
**Assess Response and Relapse**										X			*Day 28 (±4) of each cycle.
**Biomarker/ PD Blood Sample**	X	X								X			
**Dispense DS-1594b**		X			X	X		X					Continuous Dosing Every 12h (+/- 4h); Administer morning dose in clinic on PK days
**Administer morning dose of DS-1594b at site**		X	X	X	X	X		X					*For daily dosing only
**Collect Returned DS-1594b**<br>**Assess Compliance**					X	X		X		X			
**Survival FU, Subsequent Antileukemic Treatments & Outcomes**											X	X	Every 3 months in LTFU

Note: After completing the lead-in dosing, subjects will begin Cycle 1, Day 1 (Table 36).

Table 36: Phase 1 Dosing at the Target Dose Level

Phase 1 Dose Escalation Schedule of Events																									
**Procedure**	**SCR**	**C1**									**C2**					**C3**			**C4**		**Cx**		**30D**	**LT**	
**Day**	**-14**	**1**	**2**	**5**	**8**	**9**	**15**	**16**	**21**	**28**	**1**	**8**	**15**	**21**	**28**	**1**	**15**	**28**	**1**	**15**	**1**	**EOT**	**FU**	**FU**	**Comment**
**Window**				**±1D**	**±1D**	**±1D**	**±1D**	**±1D**	**±1D**	**±4D**	**±4D**	**±1D**	**±1D**	**±1 D**	**±4D**	**±4D**	**±1D**	**±4D**	**±4D**	**±1D**	**±4D**	**±3D**	**+7D**	**±14D**	
**Informed Consent**	X																								Prior to study procedures
**Bone Marrow Aspirate/Biopsy**	X									X					X			X			X*	X			*Day 28 (±4) of Cycles 1, 2, 3, 6, 9, and EOT
**Eligibility**	X																								
**Demographics, Medical History, Disease History**	X																								WHO classification, cytogenetics, mutations, prior therapy
**Vital Signs**	X	X	X	X	X		X				X					X	X		X		X	X			Blood pressure, pulse rate, respiratory rate, and temperature taken ~5 min prior to ECG measurements
**ECG (in triplicate) by 12-lead or Holter**	X	X	X	X	X	X*	X		X		X					X			X		X**	X			*For daily dosing only<br>**Cycles 5 to 8 only.<br>Triplicate ECGs ≥1 minute apart, supine/semi-recumbent. Prior to PK<br>Section 8.5 and Table 22 for timing of ECGs (additional

Phase 1 Dose Escalation Schedule of Events

Procedure	SCR	C1									C2					C3			C4		Cx				
Day	-14	1	2	5	8	9	15	16	21	28	1	8	15	21	28	1	15	28	1	15	1	EOT	30D FU	LT FU	Comment
Window				±1D	±1D	±1D	±1D	±1D	±1D	±4D	±4D	±1D	±1D	±1 D	±4D	±4D	±1D	±4D	±4D	±1D	±4D	±3D	+7D	±14D	
																									ECGs required for DDI sub-study). For cohorts with lead-in Period, please refer to Table 23.
**Height**	X																								Screening procedures should be done 3 days before first dose.
**Weight**	X	X									X					X			X		X	X			
**BSA**	X																								Calculate by Mosteller formula
**SpO2**	X	X									X					X			X		X	X			
**Physical Exam**	X	X									X					X			X		X	X			
**ECOG PS**	X	X									X					X			X		X	X			
**ECHO/MUGA**	X																								≤30 days prior to first dose
**SAEs**	X	Continuously																							
**AEs**		Continuously																							
**Prior Medications, Non-Drug Therapies, and Radiotherapy**	X																								
**Concomitant Medications, Non-**		Continuously																							

Phase 1 Dose Escalation Schedule of Events

Procedure	SCR	C1									C2					C3			C4		Cx	EOT	30D FU	LT FU	Comment
Day	-14	1	2	5	8	9	15	16	21	28	1	8	15	21	28	1	15	28	1	15	1	EOT	30D FU	LT FU	Comment
Window				±1D	±1D	±1D	±1D	±1D	±1D	±4D	±4D	±1D	±1D	±1D	±4D	±4D	±1D	±4D	±4D	±1D	±4D	±3D	+7D	±14D	
**Drug Therapies, and Radiotherapy**																									
**Hematology, INR, Chemistry**	X	X	≥ 3 times weekly								≥ 2 times weekly								1-2 times weekly			X			Screening procedures should be done 3 days before first dose.<br>CBC with Differential, PLT, Serum Creatinine, Electrolytes, Glucose, Uric acid, calcium, magnesium, BUN<br>After C6, less frequent at guidance of PI
**Ferritin, LDH, CRP, Fibrinogen, PT/PTT, and Cytokine panel (IL-6, IFN-γ, and TNF-α)**	X	X	≥ 2 times weekly																			X			Monitor after Cycle 1, if clinically indicated
**Hepatic Labs**	X	X	≥ 3 times weekly								≥ 2 times weekly								1-2 times weekly			X			AST, ALT, Alk phos, Total Bilirubin (fractionated)
**Creatinine Kinase**	X	X	Weekly																≥ 1 per cycle			X			Fractionated if >ULN
**Troponin**	X																								Monitor on treatment, if

Phase 1 Dose Escalation Schedule of Events																									
**Procedure**	**SCR**	**C1**									**C2**					**C3**			**C4**		**Cx**		**30D**	**LT**	
**Day**	**-14**	**1**	**2**	**5**	**8**	**9**	**15**	**16**	**21**	**28**	**1**	**8**	**15**	**21**	**28**	**1**	**15**	**28**	**1**	**15**	**1**	**EOT**	**FU**	**FU**	**Comment**
**Window**				**±1D**	**±1D**	**±1D**	**±1D**	**±1D**	**±1D**	**±4D**	**±4D**	**±1D**	**±1D**	**±1D**	**±4D**	**±4D**	**±1D**	**±4D**	**±4D**	**±1D**	**±4D**	**±3D**	**+7D**	**±14D**	
																									clinically indicated
**Urinalysis**	X	X	≥ 1 per cycle																			X			Screening procedures should be done 3 days before first dose.
**Pregnancy Test**	X	≥ 1 per cycle																				X	X		For women of childbearing potential, document the results of a negative urine or serum pregnancy test within 72 hours before first dose and monthly while on treatment.
**Plasma alpha-MSH**	X									X						X*					X*	X			* Once every 3 cycles beginning at Cycle 3
**Fasting morning ACTH, aldosterone, renin, and cortisol**	X									X						X*					X*	X			* Once every 3 cycles beginning at Cycle 3
**HIV Ab Test**	X																								If required by local regulations; ≤28 days of first dose

Phase 1 Dose Escalation Schedule of Events																									
**Procedure**	**SCR**	**C1**									**C2**					**C3**			**C4**		**Cx**	**EOT**	**30D FU**	**LT FU**	**Comment**
**Day**	**-14**	**1**	**2**	**5**	**8**	**9**	**15**	**16**	**21**	**28**	**1**	**8**	**15**	**21**	**28**	**1**	**15**	**28**	**1**	**15**	**1**				
**Window**				**±1D**	**±1D**	**±1D**	**±1D**	**±1D**	**±1D**	**±4D**	**±4D**	**±1D**	**±1D**	**±1 D**	**±4D**	**±4D**	**±1D**	**±4D**	**±4D**	**±1D**	**±4D**	**±3D**	**+7D**	**±14D**	
**HBsAg, HC Ab**	X																								≤28 days of first dose
**PK**		X	X	X	X	X*	X	X*	X		X					X			X		X**				*For daily dosing only<br>**Cycles 5 to 8 only.<br>See Table 15 (BID dosing) and Table 16 (QD dosing) for timing of sampling, Table 21 for food-effect sub-study, and Table 19 and Table 20 for DDI sub-study. Visit windows not applicable for PK sub-studies.<br>For cohorts with Lead-in Period, please refer to Table 17.
**Assess Response and Relapse**										X					X			X			X*	X			*Day 28 (±4) of each cycle.
**Biomarker/ PD Blood Sample**	X	X			X		X		X	X			X			X			X			X			
**Dispense DS-1594b**		X									X				X			X		X	X				Continuous Dosing Every 12h (+/- 4h); Administer

Phase 1 Dose Escalation Schedule of Events																									
**Procedure**	**SCR**	**C1**									**C2**					**C3**			**C4**		**Cx**		**30D FU**	**LT FU**	
**Day**	**-14**	**1**	**2**	**5**	**8**	**9**	**15**	**16**	**21**	**28**	**1**	**8**	**15**	**21**	**28**	**1**	**15**	**28**	**1**	**15**	**1**	**EOT**			**Comment**
**Window**				**±1D**	**±1D**	**±1D**	**±1D**	**±1D**	**±1D**	**±4D**	**±4D**	**±1D**	**±1D**	**±1D**	**±4D**	**±4D**	**±1D**	**±4D**	**±4D**	**±1D**	**±4D**	**±3D**	**+7D**	**±14D**	
																									morning dose in clinic on PK days
**Administer morning dose of DS-1594b at site**		X	X	X	X	X*	X				X					X			X		X**				*For daily dosing only<br>** Cycles 5 and 6 only.<br>Additional instruction for sub-studies:<br>For DDI sub-study, administer morning dose on-site on PK days (C1D18, C1D19, C2D10, C2D11). See Table 19 and Table 20. For food effect sub-study, administer morning dose on-site on PK days (C1D18, C1D19); see Table 21 .
**Collect Returned DS-1594b**<br>**Assess Compliance**											X					X			X		X	X			
**Survival FU, Subsequent**																							X	X	Every 3 months in LTFU

Phase 1 Dose Escalation Schedule of Events

Procedure	SCR	C1									C2					C3			C4		Cx	EOT	30D FU	LT FU	Comment
Day	-14	1	2	5	8	9	15	16	21	28	1	8	15	21	28	1	15	28	1	15	1				
Window				±1D	±1D	±1D	±1D	±1D	±1D	±4D	±4D	±1D	±1D	±1 D	±4D	±4D	±1D	±4D	±4D	±1D	±4D	±3D	+7D	±14D	
**Antileukemic Treatments & Outcomes**																									

Note: Screening (SCR) and Cycle 1, Day 1 pre-dose assessments in this table only apply to subjects enrolled in cohorts without lead-in dosing.

Table 37: Phase 2 Cohorts A & B Schedule of Events

Phase 2 Cohorts A & B																			
**Procedure**	**SCR**	**C1**						**C2**		**C3**		**C4**		**Cx**		**EOT**	**30D FU**	**LT FU**	**Comment**
**Day**	**-14**	**1**	**2**	**8**	**9**	**15**	**28**	**1**	**28**	**1**	**28**	**1**	**28**	**1**	**28**				
**Window**			**±1D**	**±1D**	**±1D**	**±1D**	**±4D**	**±4D**	**±4D**	**±4D**	**±4D**	**±4D**	**±4D**	**±4D**	**±4D**	**±3D**	**+7D**	**±14D**	
**Informed Consent**	X																		Prior to study procedures
**Bone Marrow Aspirate/Biopsy**	X						X		X		X				X*	X			*Day 28 (±4) of Cycles 1, 2, 3, 6, 9, and EOT
**Confirm NPM1-m or MLL-r**	X																		
**Eligibility Assessment**	X																		
**Demographics, Medical & Disease History**	X																		WHO classification, cytogenetics, mutations, prior therapy
**Vital Signs**	X	X		X		X		X		X		X		X		X			Blood pressure, pulse rate, respiratory rate, and temperature taken ~5 min prior to ECG measurements
**ECG (in triplicate) by 12-lead or Holter**	X	X		X		X		X		X		X		X*		X			*Cycles 5 and 6 only.<br>Triplicate ECGs ≥1 minute apart, supine/semi-recumbent. Prior to PK. See Section 8.5 and Table 24 for timing of ECGs.
**Height**	X																		Screening procedures should be done 3 days before C1D1.
**Weight**	X	X						X		X		X		X		X			
**BSA**	X																		Calculate by Mosteller formula
**SpO2**	X	X						X		X		X		X		X			
**Physical Exam**	X	X						X		X		X		X		X			

Phase 2 Cohorts A & B																			
**Procedure**	**SCR**	**C1**						**C2**		**C3**		**C4**		**Cx**		**EOT**	**30D FU**	**LT FU**	**Comment**
**Day**	**-14**	**1**	**2**	**8**	**9**	**15**	**28**	**1**	**28**	**1**	**28**	**1**	**28**	**1**	**28**				
**Window**			**±1D**	**±1D**	**±1D**	**±1D**	**±4D**	**±4D**	**±4D**	**±4D**	**±4D**	**±4D**	**±4D**	**±4D**	**±4D**	**±3D**	**+7D**	**±14D**	
**ECOG PS**	X	X						X		X		X		X		X			
**ECHO/MUGA**	X																		≤30 days prior to first dose
**SAEs**	X	Continuously																	
**AEs**		Continuously																	
**Prior Medications, Non-Drug Therapies, and Radiotherapy**	X																		
**Concomitant Medications, Non-Drug Therapies, and Radiotherapy**	X	Continuously																	
**Hematology, Coagulation, Chemistry**	X	X	≥ 3 times weekly					≥ 2 times weekly				1-2 times weekly				X			Screening procedures should be done 3 days before C1D1.<br>CBC with Differential, PLT, Serum Creatinine, Electrolytes, Glucose, Uric acid, calcium, magnesium, BUN<br>After C6, less frequent at guidance of PI
**Hepatic Labs**	X	X	≥ 3 times weekly					≥ 2 times weekly				1-2 times weekly				X			AST, ALT, Alk phos, Total Bilirubin (fractionated)
**Creatinine Kinase**	X	X	Weekly									≥ 1 per cycle				X			Fractionated if >ULN
**Troponin**	X																		Monitor on treatment, if clinically indicated
**Urinalysis**	X	X	≥ 1 per cycle													X			Screening procedures should be done 3 days before C1D1.
**Pregnancy Test**	X	≥ 1 per cycle														X	X		For women of childbearing potential, document the results

Phase 2 Cohorts A & B

Procedure	SCR	C1						C2		C3		C4		Cx		EOT	30D FU	LT FU	Comment
Day	-14	1	2	8	9	15	28	1	28	1	28	1	28	1	28				
Window			±1D	±1D	±1D	±1D	±4D	±4D	±4D	±4D	±4D	±4D	±4D	±4D	±4D	±3D	+7D	±14D	
																			of a negative urine or serum pregnancy test within 72 hours before C1D1 and monthly while on treatment.
**Plasma alpha-MSH**	X						X			X*				X*		X			* Once every 3 cycles beginning at Cycle 3
**Fasting morning ACTH, aldosterone, renin, and cortisol**	X						X			X*				X*		X			* Once every 3 cycles beginning at Cycle 3
**HIV Ab Test**	X																		If required by local regulations; ≤28 days of first dose
**HBsAg, HC Ab**	X																		≤28 days of first dose
**PK**		X	X*	X	X*	X		X		X		X		X**					*For daily dosing only<br>**Cycles 5 and 6 only<br>See Table 18 for sampling times
**Assess Response and Relapse**							X		X		X		X		X*	X			*Day 28 (±4) of each cycle.
**Biomarker/ PD Blood Sample**	X	X		X		X	X			X		X				X			
**Dispense DS-1594b**		X						X		X		X		X					Continuous Dosing Every 12h (+/- 4h); Administer morning dose in clinic on PK days
**Administer morning dose of DS-1594b at site**		X		X		X		X		X		X		X*					*Cycles 5 and 6 only
**Collect Returned DS-1594b, Assess Compliance**								X		X		X		X		X			

Phase 2 Cohorts A & B																			
**Procedure**	**SCR**	**C1**						**C2**		**C3**		**C4**		**Cx**		**EOT**	**30D FU**	**LT FU**	**Comment**
**Day**	**-14**	**1**	**2**	**8**	**9**	**15**	**28**	**1**	**28**	**1**	**28**	**1**	**28**	**1**	**28**				
**Window**			**±1D**	**±1D**	**±1D**	**±1D**	**±4D**	**±4D**	**±4D**	**±4D**	**±4D**	**±4D**	**±4D**	**±4D**	**±4D**	**±3D**	**+7D**	**±14D**	
**Survival FU, Subsequent Anti-leukemic Treatments & Outcomes**																	X	X	Every 3 months in LTFU

Table 38: Phase 2 Cohort C Schedule of Events

Phase 2 Cohort C																						
**Procedure**	**SCR**	**C1**							**C2 - C3**			**C4**			**Cx**			**EOT**	**30D FU**	**LT FU**	**Comment**
**Day**	**-14**	**1**	**2-7**	**8**	**9**	**15**	**21**	**28**	**1**	**2-7**	**28**	**1**	**2-7**	**28**	**1**	**2-7**	**28**				
**Window**			**±1D**	**±1D**	**±1D**	**±1D**	**±4D**	**±4D**	**±4D**		**±4D**	**±4D**		**±4D**	**±4D**		**±4D**	**±3D**	**+7D**	**±14D**	
**Informed Consent**	X																				Prior to study procedures
**Bone Marrow Aspirate/Biopsy**	X						X	X			X						X*	X			Adjust venetoclax administration per Section 5.4 based on Cycle 1 Day 21 bone marrow evaluation<br>*Day 28 (±4) of Cycles 1, 2, 3, 6, 9, and EOT
**Confirm NPM1-m or MLL-r**	X																				
**Eligibility Assessment**	X																				
**Demographics, Medical History, Disease History**	X																				WHO classification, cytogenetics, mutations, prior therapy
**Vital Signs**	X	X		X		X			X			X			X			X			Blood pressure, pulse rate, respiratory rate, and temperature taken ~5 min prior to ECG measurements
**ECG (in triplicate) by 12-lead or Holter**	X	X		X		X			X			X			X*			X			*Cycles 5 and 6 only<br>Triplicate ECGs ≥1 minute apart, supine/semi-recumbent. Prior to PK<br>Section 8.5 and Table 24 for timing of ECGs.
**Height**	X																				Screening procedures should be done 3 days before C1D1.
**Weight**	X	X							X			X			X			X			
**BSA**	X																				Calculate by Mosteller formula

Phase 2 Cohort C

Procedure	SCR	C1							C2 - C3			C4			Cx			EOT	30D FU	LT FU	Comment
**Day**	**-14**	**1**	**2-7**	**8**	**9**	**15**	**21**	**28**	**1**	**2-7**	**28**	**1**	**2-7**	**28**	**1**	**2-7**	**28**				
**Window**			**±1D**	**±1D**	**±1D**	**±1D**	**±4D**	**±4D**	**±4D**		**±4D**	**±4D**		**±4D**	**±4D**		**±4D**	**±3D**	**+7D**	**±14D**	
**SpO2**	X	X							X			X			X			X			
**Physical Exam**	X	X							X			X			X			X			
**ECOG PS**	X	X							X			X			X			X			
**ECHO/MUGA**	X																				≤30 days prior to first dose
**SAEs**	X	Continuously																			
**AEs**		Continuously																			
**Prior Medications, Non-Drug Therapies, and Radiotherapy**	X																				
**Concomitant Medications, Non-Drug Therapies, and Radiotherapy**	X	Continuously																			
**Hematology, Coagulation, Chemistry**	X	X	≥ 3 times weekly						≥ 2 times weekly			1-2 times weekly						X			Screening procedures should be done 3 days before C1D1.<br>CBC with Differential, PLT, Serum Creatinine, Electrolytes, Glucose, Uric acid, calcium, magnesium, BUN<br>After C6, less frequent at guidance of PI
**Hepatic Labs**	X	X	≥ 3 times weekly						≥ 2 times weekly			1-2 times weekly						X			AST, ALT, Alk phos, Total Bilirubin (fractionated)
**Creatinine Kinase**	X	X	Weekly									≥ 1 per cycle						X			Fractionated if >ULN
**Troponin**	X																				Monitor on treatment, if clinically indicated

Phase 2 Cohort C

Procedure	SCR	C1							C2 - C3			C4			Cx			EOT	30D FU	LT FU	Comment
**Day**	**-14**	**1**	**2-7**	**8**	**9**	**15**	**21**	**28**	**1**	**2-7**	**28**	**1**	**2-7**	**28**	**1**	**2-7**	**28**				
**Window**			**±1D**	**±1D**	**±1D**	**±1D**	**±4D**	**±4D**	**±4D**		**±4D**	**±4D**		**±4D**	**±4D**		**±4D**	**±3D**	**+7D**	**±14D**	
**Urinalysis**	X	X	≥ 1 per cycle															X			Screening procedures should be done 3 days before C1D1.
**Pregnancy Test**	X	≥ 1 per cycle																X	X		For women of childbearing potential, document the results of a negative urine or serum pregnancy test within 72 hours before C1D1 and monthly while on treatment.
**Plasma alpha-MSH**	X							X	X*						X*			X			* Once every 3 cycles beginning at Cycle 3 (Not required in Cycle 2)
**Fasting morning ACTH, aldosterone, renin, and cortisol**	X							X	X*						X*			X			* Once every 3 cycles beginning at Cycle 3 (Not required in Cycle 2)
**HIV Ab Test**	X																				If required by local regulations; ≤28 days of first dose
**HBsAg, HC Ab**	X																				≤28 days of first dose
**PK**		X	X*	X	X*	X			X			X			X**						*For daily dosing only<br>**Cycles 5 and 6 only.<br>See Table 18 for timing of sampling
**Assess Response and Relapse**								X			X			X			X*	X			*Day 28 (±4) of each cycle.
**Biomarker/ PD Blood Sample**	X	X		X		X		X				X						X			
**Dispense DS-1594b**		X							X			X			X						Continuous oral dosing every 12h (+/- 4h); Administer morning dose in clinic on PK days

Phase 2 Cohort C																				
**Procedure**	**SCR**	**C1**							**C2 - C3**			**C4**			**Cx**			**EOT**	**30D FU**	**LT FU**	**Comment**
**Day**	**-14**	**1**	**2-7**	**8**	**9**	**15**	**21**	**28**	**1**	**2-7**	**28**	**1**	**2-7**	**28**	**1**	**2-7**	**28**				
**Window**			**±1D**	**±1D**	**±1D**	**±1D**	**±4D**	**±4D**	**±4D**		**±4D**	**±4D**		**±4D**	**±4D**		**±4D**	**±3D**	**+7D**	**±14D**	
**Dispense venetoclax**		X							X			X			X						Continuous oral dosing daily; schedule adjustments per Section 5.4
**Administer azacitidine**		Daily*							X*			X*	X*		X*	X*					*Administer azacitidine SQ or IV in clinic on Day 1-7 of each cycle; schedule per Section 5.4
**Administer morning dose of DS-1594b at site**		X		X		X			X			X			X*						*Cycles 5 and 6 only
**Collect Returned DS-1594b**<br>**Assess Compliance**									X			X			X			X			
**Survival FU, Subsequent Antileukemic Treatments & Outcomes**																			X	X	Every 3 months in LTFU

Table 39: Phase 2 Cohort D Schedule of Events

Phase 2 Cohorts D																									
**Procedure**	**SCR**	**C1**								**C2**					**C3**			**C4**		**Cx**	**Maintenance C1-C24**	**EOT**	**30D FU**	**LT FU**	**Comment**
**Day**	**-14**	**1**	**2**	**5**	**8**	**9**	**15**	**21**	**28**	**1**	**8**	**15**	**21**	**28**	**1**	**15**	**28**	**1**	**15**	**1**	**1**				
**Window**										**±4D**					**±4D**			**±4D**		**±4D**		**±3D**	**+7D**	**±14D**	
**Informed Consent**	X																								Prior to study procedures
**Bone Marrow Aspirate/Biopsy**	X								X					X			X			X*		X			*Day 28 (±4) of Cycles 1, 2, 3, 6, 9, and EOT
**Eligibility**	X																								
**Demographics, Medical History, Disease History**	X																								WHO diag., cytogenetics, mutations, prior therapy
**Vital Signs**	X	X			X		X			X					X	X		X		X	X	X			Blood pressure, pulse rate, respiratory rate, and temperature taken ~5 min prior to ECG measurements
**ECG (in triplicate) by 12-lead or Holter**	X	X			X		X			X					X			X		X*	X	X			*Cycles 5 and 6 only. Triplicate ECGs obtained at least 1 minute apart, in a supine/semi-recumbent position. Obtain prior to PK Section 8.5 and Table 24 for timing of ECGs.

Phase 2 Cohorts D

Procedure	SCR	C1								C2					C3			C4		Cx	Maintenance C1-C24	EOT	30D FU	LT FU	Comment
**Day**	**-14**	**1**	**2**	**5**	**8**	**9**	**15**	**21**	**28**	**1**	**8**	**15**	**21**	**28**	**1**	**15**	**28**	**1**	**15**	**1**	**1**				
**Window**										**±4D**					**±4D**			**±4D**		**±4D**		**±3D**	**+7D**	**±14D**	
**Height**	X																								Screening procedures should be done 3 days before C1D1.
**Weight**	X	X								X					X			X		X	X	X			
**BSA**	X																								Calculate by Mosteller formula
**SpO2**	X	X								X					X			X		X	X	X			
**Physical Exam**	X	X								X					X			X		X	X	X			
**ECOG PS**	X	X								X					X			X		X	X	X			
**ECHO/MUGA**	X																								≤30 days prior to first dose
**SAEs**	X	Continuously																							
**Non SAEs**	X	Continuously																							
**Prior Meds, Non-Drug Therapies, and Radiotherapy**	X																								
**Concomitant Medications, Non-Drug Therapies, and Radiotherapy**		Continuously																							
**Hematology, Chemistry Tests; Coagulation**	X	X	≥ 3 times weekly							≥ 2 times weekly								1-2 times weekly			X	X			Screening procedures should be done 3 days before C1D1.

Phase 2 Cohorts D

Procedure	SCR	C1								C2					C3			C4		Cx	Maintenance C1-C24	EOT	30D FU	LT FU	Comment
**Day**	**-14**	**1**	**2**	**5**	**8**	**9**	**15**	**21**	**28**	**1**	**8**	**15**	**21**	**28**	**1**	**15**	**28**	**1**	**15**	**1**	**1**				
**Window**										**±4D**					**±4D**			**±4D**		**±4D**		**±3D**	**+7D**	**±14D**	
																									CBC with Differential, PLT, Serum Creatinine, Electrolytes, Glucose, Uric acid, calcium, magnesium, BUN<br>After C6, less frequent at guidance of PI
**Hepatic Labs**	X	X	≥ 3 times weekly							≥ 2 times weekly								1-2 times weekly			≥ 1 per cycle	X			AST, ALT, Alk phos, total Bilirubin (fractionated)
**Creatinine Kinase**	X	X	Weekly															≥ 1 per cycle			≥ 1 per cycle	X			Fractionated if >ULN
**Troponin**	X																								Monitor on treatment, if clinically indicated
**Urinalysis**	X	X	≥ 1 per cycle																		≥ 1 per cycle	X			Screening procedures should be done 3 days before C1D1.

Phase 2 Cohorts D																									
**Procedure**	**SCR**	**C1**								**C2**					**C3**			**C4**		**Cx**	**Maintenance C1-C24**	**EOT**	**30D FU**	**LT FU**	**Comment**
**Day**	**-14**	**1**	**2**	**5**	**8**	**9**	**15**	**21**	**28**	**1**	**8**	**15**	**21**	**28**	**1**	**15**	**28**	**1**	**15**	**1**	**1**				
**Window**										**±4D**					**±4D**			**±4D**		**±4D**		**±3D**	**+7D**	**±14D**	
**Pregnancy Test**	X	≥ 1 per cycle																			≥ 1 per cycle	X			For women of childbearing potential, document the results of a negative urine or serum pregnancy test within 72 hours before C1D1 and monthly while on treatment.
**Plasma alpha-MSH**	X								X						X*					X*	X*	X			* Once every 3 cycles beginning at Cycle 3
**Fasting morning ACTH, aldosterone, renin, and cortisol**	X								X						X*					X*	X*	X			* Once every 3 cycles beginning at Cycle 3
**HIV Ab Test**	X																								As required by local regulations, to be done within 28 days prior to Enrollment.
**HBsAg, HC Ab**	X																								To be done within 28 days prior to Enrollment.
**PK**		X	X*		X	X*	X			X					X			X		X**					*For daily dosing only

Phase 2 Cohorts D																									
**Procedure**	**SCR**	**C1**								**C2**					**C3**			**C4**		**Cx**	**Maintenance C1-C24**	**EOT**	**30D FU**	**LT FU**	**Comment**
**Day**	**-14**	**1**	**2**	**5**	**8**	**9**	**15**	**21**	**28**	**1**	**8**	**15**	**21**	**28**	**1**	**15**	**28**	**1**	**15**	**1**	**1**				
**Window**										**±4D**					**±4D**			**±4D**		**±4D**		**±3D**	**+7D**	**±14D**	
																									**Cycles 5 and 6 only.<br>See Table 18 for timing of sampling
**Assess Response and Relapse**									X						X			X		X	X	X			
**Biomarker/ PD Blood Sample**	X	X			X		X	X	X			X			X			X				X			TBD after the phase I
**Dispense DS-1594b**		X								X					X			X		X	X				Continuous Dosing Every 12h (+/- 4h); Administer morning dose in clinic on PK days
**Administer morning dose of DS-1594b at site**		X			X		X			X					X			X		X*					*Not required after Cycle 6
**Collect Returned DS-1594b, Assess Compliance**										X					X			X		X	X	X			
**Survival FU, Subsequent Antileukemic Treatments & Outcomes**																							X	X	Every 3 months in LTFU

11. REFERENCES

1. Winer ES and Stone RM. Novel therapy in acute myeloid leukemia (AML): moving toward targeted approaches. Ther Adv Hematol. 2019;10:1-18.
2. Papaemmanuil E, Gerstung M, Bullinger L, Gaidzik VI, Paschka P, Roberts ND, et al. Genomic classification and prognosis in acute myeloid leukemia. N Engl J Med. 2016;374:2209-21.
3. Metzeler KH, Herold T, Thurley MR, Amler S, Sauerland MC, Gorlich D, et al. Spectrum and prognostic relevance of driver gene mutations in acute myeloid leukemia. Blood. 2016;128:686-98.
4. Estey EH. Treatment of relapsed and refractory acute myelogenous leukemia. Leukemia 2000;14:476-9.
5. Ravandi F, Cortes J, Faderl S, et al. Characteristics and outcome of subjects with acute myeloid leukemia refractory to 1 cycle of high-dose cytarabine-based induction chemotherapy. Blood 2010;116:5818-23.
6. Stanisic S, Kalaycio M. Treatment of refractory and relapsed acute myelogenous leukemia. Expert Rev Anticancer Ther 2002;2:287-95.
7. Ravandi F, Cortes J, Faderl S, et al. Characteristics and outcome of subjects with acute myeloid leukemia refractory to 1 cycle of high-dose cytarabine-based induction chemotherapy. Blood 2010;116:5818-23.
8. Paul S, Kantarjian H, Jabbour EJ. Adult acute lymphoblastic leukemia. Mayo Clin Proc 2016;91:1645-66
9. Pui CH, Robinson LL, Look AT. Acute lymphoblastic leukemia. Lancet. 2008;371:1030-43.
10. Dimartino JF and Cleary ML. Mll rearrangements in haematological malignancies: lessons from clinical and biological studies. Br J Haematol. 1999;106:614-26.
11. Marschalek R. Mechanisms of leukemogenesis by MLL fusion proteins. Br J Haematol. 2011;152:141-54.
12. Meyer C, Burmeister T, Groger D, et al. The MLL recombinome of acute leukemias in 2017. Leukemia. 2018;32: 273–84.
13. Kuhn MW, Song E, Feng Z, Sinha A, Chen CW, Deshpande AJ et al. Targeting chromatin regulators inhibits leukemogenic gene expression in NPM1 mutant leukemia. Cancer Discov. 2016;6:1166-81.
14. Thiede C, Koch S, Creutzig E, et al. Prevalence and prognostic impact of NPM1 mutations in 1485 adult subjects with acute myeloid leukemia (AML). Blood. 2006;107:4011-20.

15. Dunlap JB, Leonard J, Rosenberg M, et al. The combination of NPM1, DNMT3A, and IDH1/2 mutations leads to inferior overall survival in AML. Am J Hematol. 2019; 94:913-20.

16. Grembecka J, Belcher AM, Hartley T and Cierpicki T. Molecular basis of the mixed lineage leukemia-menin interaction: implications for targeting mixed lineage leukemias. J Biol Chem. 2010;285:40690-8.

17. Yokoyama A, Somervaille TC, Smith KS, et al. The menin tumor suppressor protein is an essential oncogenic cofactor for MLL-associated leukemogenesis. Cell. 2005;123:207-18.

18. Borkin D et al. Pharmacologic inhibition of the Menin-MLL interaction blocks progression of MLL leukemia in vivo. Cancer Cell. 2015;27:589-602.

19. Slany RK. When epigenetics kills: MLL fusion proteins in leukemia. Hematol Oncol. 2005;23:1-9.

20. Tomizawa D, Koh K, Sato T, et al. Outcome of risk-based therapy for infant acute lymphoblastic leukemia with or without an MLL gene rearrangement, with emphasis on late effects: a final report of two consecutive studies, MLL96 and MLL98, of the Japan Infant Leukemia Study Group. Leukemia. 2007;21:2258-63.

21. Jabbour E et al, JAMA Onc 2018 Feb 1;4(2):230-4.

22. Jabbour E et al, Cancer 2018 Oct 15;124(20):4044-55.

23. Cheson B et al, J Clin Oncol. 2003 Dec 15;21(24):4642-9.

24. Dohner H et al, Blood. 2017 Jan 26;129(4):424-47

25. Arber DA, Orazi A, Hasserjian R, et al. The 2016 revision to the World Health Organization classification of myeloid neoplasms and acute leukemia. Blood. 2016;127(20):2391-2405.

26. Swerdlow SH, Campo E, Pileri SA, et al. The 2016 revision of the World Health Organization classification of lymphoid neoplasms.

27. Thall PF, Simon R, Etsey EH. Bayesian sequential monitoring designs for single-arm clinical trials with multiple outcomes. Stat Med 1995;14:357-9.

28. Perl AE, Martinelli G, Cortes JE, et al. Gilteritinib or Chemotherapy for Relapsed or Refractory FLT3-Mutated AML. N Engl J Med. 2019;381(18):1728-40.

29. DiNardo CD, Stein EM, de Botton S, et al. Durable Remissions with Ivosidenib in IDH1-Mutated Relapsed or Refractory AML. N Engl J Med. 2018;378(25):2386-98.

30. Stein EM, DiNardo CD, Pollyea DA, et al. Enasidenib in mutant IDH2 relapsed or refractory acute myeloid leukemia. Blood. 2017;130(6):722-31.

31. Daver N, Wei AH, Pollyea DA, et al. New directions for emerging therapies in acute myeloid leukemia: the next chapter. Blood Cancer J. 2020;10(10):107.

32. Liu M, Wang SJ, Ji Y. The i3+3 design for phase I clinical trials. J Biopharm Stat. 2020 Mar;30(2):294-304.

33. Zhou H, Murray TA, Pan H, Yuan Y. Comparative review of novel model-assisted designs for phase I clinical trials. Stat Med. 2018 Jun 30; 37(14):2208-22.

34. Liu S and Yuan Y. Bayesian optimal interval designs for phase I clinical trials. Journal of the Royal Statistical Society. Series C (Applied Statistics). 2015; 64(3): 507-23.

35. Yuan Y, Hess KR, Hilsenbeck SG, Gilbert MR. Bayesian Optimal Interval Design: A Simple and Well-Performing Design for Phase I Oncology Trials. Clin Cancer Res. 2016; 22(17): 4291-301.

36. Montesinos P, Bergua JM, Vellenga E, Rayón C, Parody R, de la Serna J, et al. Differentiation syndrome in patients with acute promyelocytic leukemia treated with all-trans retinoic acid and anthracycline chemotherapy: characteristics, outcome, and prognostic factors. Blood. 2009 Jan 22;113(4):775-83.

37. Norsworthy KJ, Mulkey F, Scott EC, Ward AF, Przepiorka D, Charlab R, et al. Differentiation Syndrome with Ivosidenib and Enasidenib Treatment in Patients with Relapsed or Refractory IDH-Mutated AML: A U.S. Food and Drug Administration Systematic Analysis. Clin Cancer Res. 2020 Aug 15;26(16):4280-8.

38. Stahl M, Tallman MS. Differentiation syndrome in acute promyelocytic leukaemia. Br J Haematol. 2019 Oct;187(2):157-62.

39. Pellegrino M, Sciambi A, Treusch S, Gokhale K, Jacob J, Chen TX, et al. High-throughput single-cell DNA sequencing of AML tumors with droplet microfluidics. Genome Research. 2018;28: 1345-1352. PMID: 30087104

40. Morita K, Wang F, Jahn K, Kuipers J, Yan Y, Mathews J, et al. Clonal evolution of acute myeloid leukemia revealed by high-throughput single-cell genomics. Nat Commun. 2020;11: 5327. PMID: 33087716

41. Miles LA, Bowman RL, Merlinsky TR, Csete IS, Ooi AT, Durruthy-Durruthy R, et al. Single-cell mutation analysis of clonal evolution in myeloid malignancies. Nature. 2020; 587: 477-482. PMID: 33116311

42. Fiskus W, Mill CP, Nabet B, Perera D, Birdwell C, Manshouri T, et al. Superior efficacy of co-targeting GFI1/KDM1A and BRD4 against AML and post-MPN secondary AML cells. Blood cancer journal. 2021;11(5):98.

43. Fiskus W, Cai T, DiNardo CD, Kornblau SM, Borthakur G, Kadia TM, et al. Superior efficacy of cotreatment with BET protein inhibitor and BCL2 or MCL1 inhibitor against AML blast progenitor cells. Blood cancer journal. 2019;9(2):4.

44. Saenz DT, Fiskus W, Manshouri T, Mill CP, Qian Y, Raina K, et al. Targeting nuclear beta-catenin as therapy for post-myeloproliferative neoplasm secondary AML. Leukemia. 2019;33(6):1373-86.
