# Supplementary material for: A phase 1/2 study of DS-1594 menin inhibitor in relapsed/refractory acute leukemias
Source: J Hematol Oncol. 2025 Nov 27;18:108. doi: 10.1186/s13045-025-01757-4 (PMC12661886; doi:10.1186/s13045-025-01757-4)
Supplement: Supplementary file 2 — Supplementary Material 2 [file 13045_2025_1757_MOESM2_ESM.docx]

**A Phase 1/2 Study of DS-1594 Menin Inhibitor in Relapsed/Refractory Acute Leukemias**

Jayastu Senapati^1^, Marina Konopleva^1^, Ghayas C. Issa^1^, Elias Jabbour^1^, Tapan Kadia^1^, Courtney DiNardo^1^, Gautam Borthakur^1^, Naveen Pemmaraju^1^, Nicholas J. Short^1^, Musa Yilmaz^1^, Indraneel Deshmukh^1^, Joie Alvarez^1^, Sanam Loghavi^2^, Guilin Tang^2^ ,Hussein A. Abbas^1^, Michael Andreeff^1^, Kapil Bhalla^1^, Narasimha M. Midde^3^, Nabil Said^3^, Amy Noyalis^3^, Derek E.Mires^3^, Jing Ning^4^, Lianchun Xiao^4^, Farhad Ravandi^1^, Guillermo Garcia-Manero^1^, Hagop M. Kantarjian^1^, Naval G. Daver^1^

^1^Department of Leukemia, The University of Texas MD Anderson Cancer Center, Houston

^2^Department of Hematopathology, The University of Texas MD Anderson Cancer Center, Houston

^3^Daiichi Sankyo, Inc, Basking Ridge, New Jersey

^4^Department of Biostatistics, The University of Texas MD Anderson Cancer Center, Houston

**Running title:** DS-1594 in relapsed/refractory acute leukemias

**Keywords:** Acute Myeloid leukemia, Menin inhibitor, *KMT2A*, *NPM1*, Differentiation Syndrome

Pages: 5

Table: 2

Figures: 3

**Tables and figures in this document are arranged in the sequence they are described in the main manuscript**

**Figure S1.** Phase 1 and 2 study design with phase 1 dose escalation schema


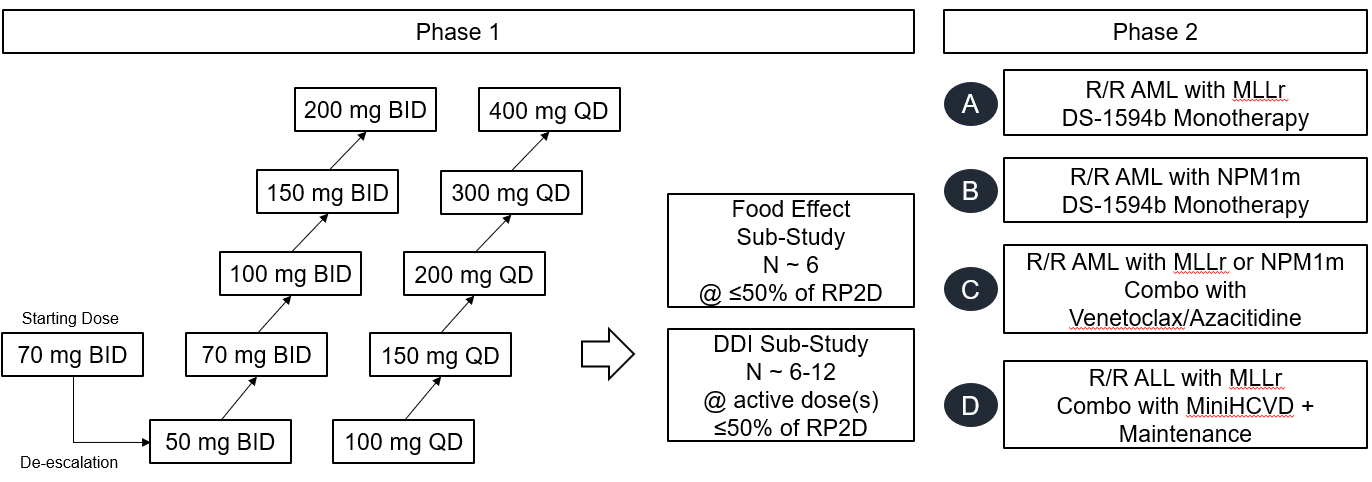


**Abbreviations**: BID, twice daily; QD, once daily; R/R, relapsed/refractory; AML, acute myeloid leukemia; r, rearrangement; combo, combination

**Figure S2.** Lead in dosing strategy starting with 20 mg QD


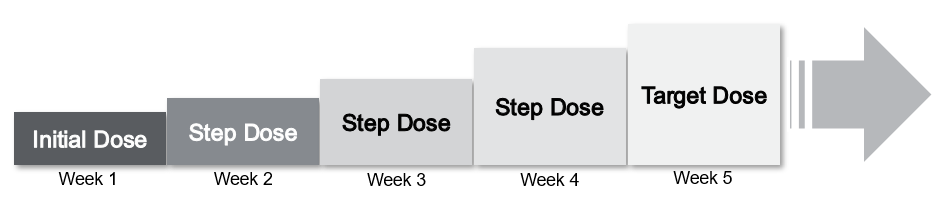


**Table S1.** Lead in dosing strategy*

| Cohort | Initial Dose (mg)  [Lead-in Period] | Step 2 (mg)  [Lead-in Period] | Step 3 (mg)  [Lead-in Period] | Step 4 (mg)  [Lead-in Period] | Target Dose (mg)  [Treatment Cycle 1 Day 1] | DLT period  (lead-in period +28-days at target dose) |
| --- | --- | --- | --- | --- | --- | --- |
| 4 | 20 | - | - | - | 50 | 35 |
| 5 | 20 | 50 | - | - | 100 | 42 |
| 6 | 20 | 50 | 100 | - | 150 | 49 |
| 7 | 20 | 50 | 100 | 150 | 200 | 56 |
| 8 | 20 | 50 | 100 | 200 | 300 | 56 |
| 9 | 20 | 50 | 100 | 200 | 400 | 56 |

*Detailed in study protocol, Section 3

**Table S2:** Characteristics of patients who developed differentiation syndrome (DS) during treatment on the clinical trial

| Patient | Dose cohort | Diagnosis | Genetics | Prior MI | DS Grade | Time of DS | DS therapy | DS Manifestation | Outcomes | Best response | Total cycles |
| --- | --- | --- | --- | --- | --- | --- | --- | --- | --- | --- | --- |
| 19/F | 1  (70 mg BID) | AML, 9^th^ line of therapy | *KMT2A*-r, *FLT3*-TKD, *WT1* | N | 1 | C1 | Drug held,  Steroids,  Hydroxyurea | Leukocytosis  Bone pains | DS Improved  Study drug restarted | 25% blast reduction (after C2) | 4  PNA |
| 78/M | 1  (70 mg BID) | AML, 3^rd^ line of therapy | *ASXL1, RUNX1, TET2* | N | 4 | C1 | Drug held  Steroids  Hydroxyurea | Shortness of breath, Hypoxia | DS improved  Study drug discontinued (DLT) | No response | 1 |
| 82/M | 2  (100 mg QD) | AML, 4^th^ line of therapy | *ASXL1, RUNX1, NOTCH1* | N | 3* | C1 | Drug held and dose reduced to 50 mg/day | Leukocytosis | Leukemia progression  Study drug discontinued, started on chemotherapy | Not evaluable | 1 |
| 21/M | 2  (50 mg BID) | AML, 3^rd^ line of therapy | *KMT2A*-r, *NRAS, TP53* | N | 2* | C1 (after 3 doses of the drug) | Drug held, Steroids, Hydroxyurea | Leukocytosis | DS improved  CNS bleed  Study drug discontinued | Not evaluable | 1 |
| 25/M | 2  (100 mg QD) | AML, 8^th^ line of therapy | *KMT2A*-r, *NRAS,* | Y | 2 | C1 | Drug held,  Steroids | Back pain | DS Improved  Study drug restarted | No response | 2 |

*These patients had only leukocytosis and possible DS, though leukemia progression was also a possibility

**Abbreviations**: MI, menin inhibitor; DS, differentiation syndrome; BID, twice daily; C, cycles; DLT, dose limiting toxicity; CNS, central nervous system; QD, once daily

**Figure S3.** Mean±SEM DS-1594 Concentration-Time Profile by Phase 1 dose Cohort

**S3A-E.** Linear Scale; **S3F-J.** Logarithmic scale

| **A-E: Linear Scale** | **F-J: Logarithmic Scale** |
| --- | --- |
| A: Cohort= 20 mg QD | F: Cohort= 20 mg QD |
| 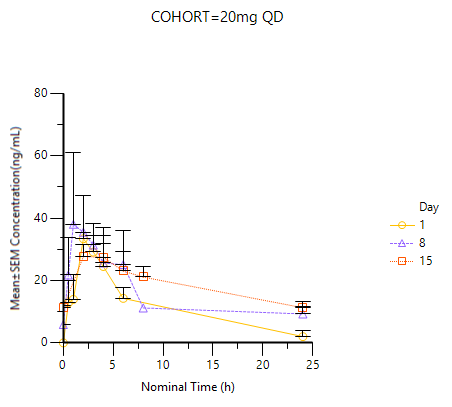 | 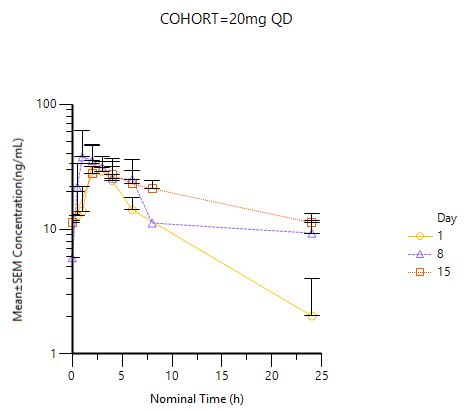 |
| B: Cohort= 50 mg BID | G: Cohort= 50 mg BID |
| 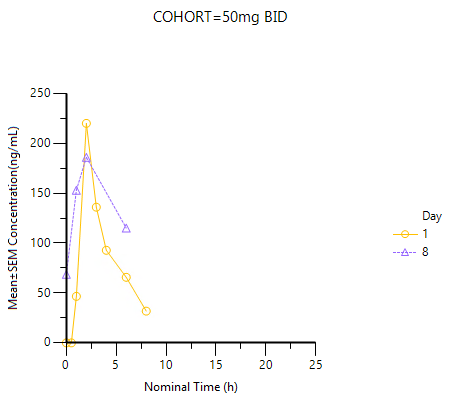 | 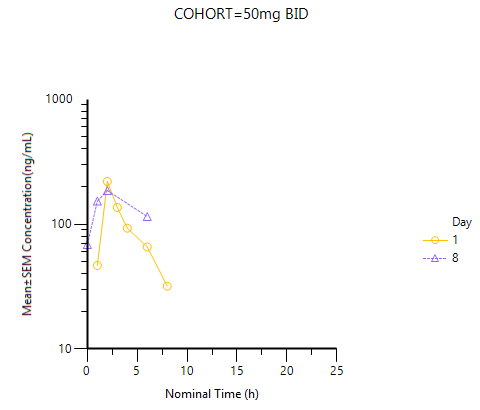 |
| C: Cohort= 50 mg QD | H: Cohort= 50 mg QD |
| 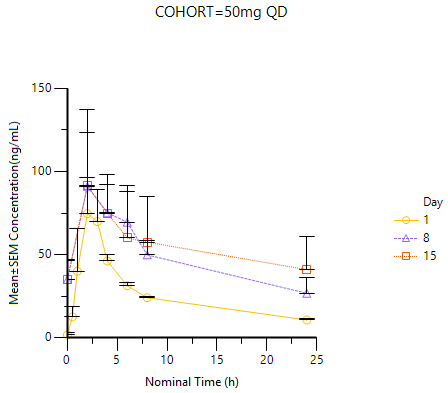 | 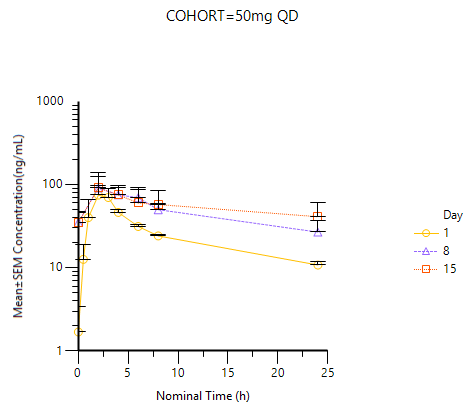 |
| D: Cohort= 70 mg BID | I: Cohort= 70 mg BID |
| 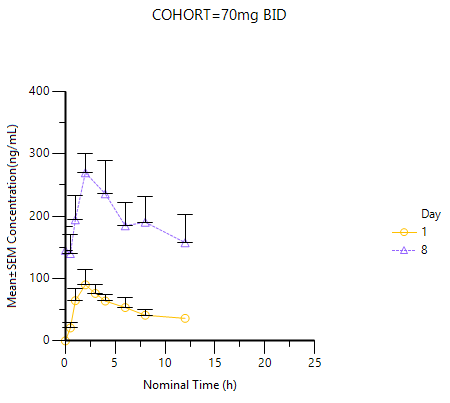 | 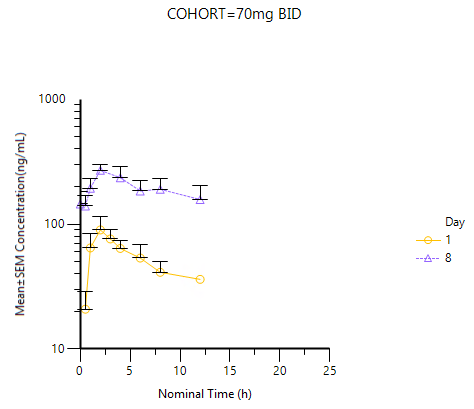 |
| E: Cohort= 100 mg QD | J: Cohort= 100 mg QD |
| 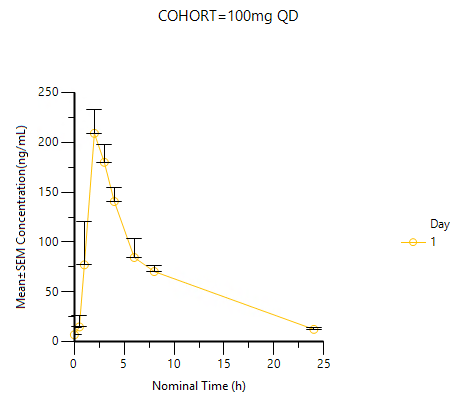 | 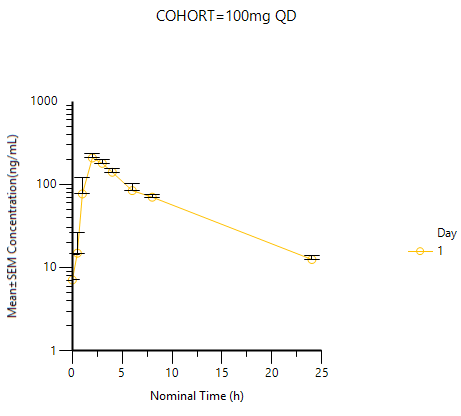 |
